# COVID-19 forecasts using Internet search information in the United States

**DOI:** 10.1038/s41598-022-15478-y

**Published:** 2022-07-07

**Authors:** Simin Ma, Shihao Yang

**Affiliations:** grid.213917.f 0000 0001 2097 4943 H. Milton Stewart School of Industrial and Systems Engineering, Georgia Institute of Technology, 755 Ferst Dr NW, Atlanta, GA 30332-0205 USA

**Keywords:** Infectious diseases, Statistics

## Abstract

As the COVID-19 ravaging through the globe, accurate forecasts of the disease spread are crucial for situational awareness, resource allocation, and public health decision-making. Alternative to the traditional disease surveillance data collected by the United States (US) Centers for Disease Control and Prevention (CDC), big data from Internet such as online search volumes also contain valuable information for tracking infectious disease dynamics such as influenza epidemic. In this study, we develop a statistical model using Internet search volume of relevant queries to track and predict COVID-19 pandemic in the United States. Inspired by the strong association between COVID-19 death trend and symptom-related search queries such as “loss of taste”, we combine search volume information with COVID-19 time series information for US national level forecasts, while leveraging the cross-state cross-resolution spatial temporal framework, pooling information from search volume and COVID-19 reports across regions for state level predictions. Lastly, we aggregate the state-level frameworks in an ensemble fashion to produce the final state-level 4-week forecasts. Our method outperforms the baseline time-series model, while performing reasonably against other publicly available benchmark models for both national and state level forecast.

## Introduction

COVID-19, an acute respiratory syndrome disease caused by novel coronavirus SARS-CoV-2, has spread to more than 200 countries worldwide, leading to more than 445 million confirmed cases and 6.04 million deaths as of Mar 5, 2022^1^. Understanding how the disease spread dynamics progress over time is much needed, given the fluid situation and the potential rapid growth of COVID-19 infections. The implementation of efficient intervention policy and the allocation of emergency resources all depend on the accurate forecasts of the disease situation^2^. Currently, machine learning methods^3–5^ and compartmental models^6–10^ are the most popular and prevailing approaches for the publicly-available COVID-19 spread forecasts, according to the weekly forecast reports compiled by the Centers for Disease Control and Prevention (CDC)^11^. On the other hand, statistical models utilizing internet search behaviors for COVID-19 predictions have not attracted much attention.

In the last decade, numerous studies have shown that Internet-based big data could be a valuable complementary data source to monitor the prevalence of infectious diseases and provide near real-time disease estimations^12–15^, alternative to the traditional surveillance approach. For example, Yang et al.^12^ provided a robust way to estimate real-time influenza situation in the United States using Google search data; Ning et al.^16^ and Yang et al.^17^ further extended the method for the regional-level and the state-level influenza estimation using the Google search data at the finer resolution; Yang et al.^18^ demonstrated the success of search data to track dengue fever in five tropical countries. Other types of internet-based data include cloud-based electronic health records^19^ and social media messages^20^. Currently, during the ongoing COVID-19 pandemic, a large amount of pandemic-related online searches is generated, indicating actual infections and general concerns, which might contain useful information to estimate and predict the disease spread.

However, building COVID-19 prediction model with online search data is undoubtedly challenging. First of all, COVID-19 pandemic is a novel disease outbreak with rapid development^1^, which creates difficulties in identifying relevant keyword queries of the search data. Even with the expert-curated queries, the online search frequency data can contain a high level of noise with many unusual spikes, due to the general searches driven by non-disease factors such as media coverage or public concern^21^. Besides, the ground truth data compiled by CDC could also be noisy with frequent retrospective revisions of daily/weekly cases accounting for the mistakes in data collection and reporting^11^.

### Related literature

The correlation between search engine data (such as Google Trends^22^, Baidu^23^, Twitter, and YouTube searches^24^) and the COVID-19 situation has been well documented for multiple countries^21,25,26^, including specific studies in China^27,28^, Europe^29,30^, India^31^, Iran^30,32^, U.S.^30,33–35^, and Spain^36^. However, these articles mostly focus on the pure correlation exercise, including correlation computation^25,26,35,36^, rank analysis^34^, and cross correlation for time delay between search peaks and COVID-19 cases/deaths^27,28,31^. None of them examines the importance among the search queries, considers the spatial–temporal structure of the data, or attempts to make weeks-ahead predictions. The relevant search queries in existing literature include general COVID-19 terms such as “coronavirus” or “COVID”^27^, public safety precautions such as “handwashing”^28^, symptom-related queries such as “loss of smell”^30,35^. However, most of the existing articles focus on a handful of query terms under a specific class, without a large-scale data-driven query identification process.

While most of existing articles argue for the potential of online search data for COVID-19 forecasts^24,25,27–31,33,35^, only a few actually build the prediction models^25,26,32,34^. Specifically, Mavragani and Gkillas^34^ demonstrates the search data predictive power via quantile regression in U.S. states. Prasanth et al.^26^ uses the search data from selected queries on a long-short term memory (LSTM) framework for U.S, U.K and India. Ayyoubzadeh et al.^32^ takes it further by combining linear regression with LSTM model to provide short-term COVID-19 cases forecast in Iran. Lampos et al.^21^ conducts a prediction study in several European countries, using transfer-learning and Gaussian processes.

However, none of the articles above fully utilizes the predictive power of internet search data by accounting for the spatial–temporal structure, including the time series information of COVID-19 or internet searches in near-by regions/areas. Other qualitative analysis, ad-hoc correlation exercise, or off-the-shelf model application are even further away from a real impact. The only internet-search-based model, to the best of our knowledge, that accounts for spatial structure is ARGONet^23^, which uses a clustering and ${L}_{1}$-penalized data augmentation technique for 2-day ahead COVID-19 cases forecast in China. So far, none of the existing Internet-search-based methods provides robust weeks-ahead forecasts for different geographical areas in the United States that account for spatial correlations.

### Our contribution

In this paper, we propose a simple framework with Google search queries for United States national and state level 4-week-ahead COVID-19 deaths forecasts. In particular, we identify relevant queries through a large-scale cross-correlation exercise, and de-noise the search frequency data from unusual spikes using inter-quantile-range method. We detect strong correlation between lagged Google search data and COVID-19 deaths with symptom-related terms, and select important Google search queries among the large-batch of related terms to increase the robustness and interpretability of our forecasts. We then utilize the detected predictive power and combine the selected Google search data and lagged COVID-19 time series information to produce national level forecasts. We further incorporate a multi-resolution spatial temporal framework for state-level forecasts, leveraging cross-state, cross-region COVID-19 and Google search information, accounting for the correlations in infections beyond geographical proximity^37^. Unlike the clustering technique in ARGONet^23^, our state level method exhibits stronger internal spatial structure and better model interpretability. Lastly, we incorporate a winner-takes-all mechanism to generate more coherent final United States national and state level predictions. Numerical comparisons show that our method performs competitively with other publicly available COVID-19 forecasts. The success of our method demonstrates that previously-developed models for influenza predictions^12,17^ using online search data can be re-purposed for accurate and robust forecasts of COVID-19, further emphasizing the general applicability of our method and the power of big data disease detection.

## Methods

### Data acquisition and pre-processing

This paper focuses on the 50 states of the United States, plus Washington D.C. We use confirmed cases, confirmed death and Google search query frequencies as inputs.

#### COVID-19 reporting data

We use reported COVID-19 confirmed cases and death of United States from New York Times (NYT)^38^ as features in our model. This data is collected from January 21, 2020, to Mar 5, 2022.

When comparing against other Centers for Disease Control and Prevention (CDC) official predictions, we use COVID-19 confirmed cases and death from JHU CSSE COVID-19 dataset^39^ as the ground-truth. This data is curated dataset used by the CDC at their official website, collected from January 22, 2020, to Mar 5, 2022. We do not use JHU COVID-19 dataset as input features in our model because JHU COVID-19 dataset retrospectively corrects past confirmed cases and death due to reporting error, and federal and state policy changes, while NYT dataset does not revise past data, which gives more realistic forecasts.

#### Google search data

The online search data used in this paper is obtained from Google Trends^22^, where one can obtain the search frequencies of a term of interest in a specific region and time frame by typing in the search query on the website. With Google Trends API, we can obtain a daily time series of the search frequencies for the term of interest, including all searches that contain all its words (un-normalized). The search term’s frequencies time series from Google Trends is based on a sampling approach, which looks at the search query representative of all raw Google searches frequencies^22^.

This paper uses 256 top searched COVID-19 related Google search queries, including common searched COVID related terms, COVID related symptoms, COVID pandemic policies implemented, COVID related resource allocated, etc. Example terms include “Coronavirus”, “COVID 19”, “COVID Vaccine”, “loss of taste”, “loss of smell”, “cough”, and “fever”. We obtain all the Google search queries’ daily frequencies for national and state level. Regional level Google search queries are obtained by simply summing up the state level Google search query volumes that are in the region. Note that some of the search terms might seem identical, e.g. “coronavirus vaccine” and “covid 19 vaccine”, but we treat them separately in our model due to linguistic heterogeneity, as terms with similar meaning but differently phrased are embedded with different search frequencies by the public due to different linguistic preferences.

Google Trends also truncates data to 0 if the search volume for the query is too low. Consequently, for a given query and state, the zeros in Google Trends data indicate missing data due to low volume of searches for the specified query and state, which is very common in practice. We account for the high level of sparsity in the state level data by borrowing information from regional level to “enrich” state-level sparsity through a weighted average of state-level search frequency (2/3 weight) and regional-level search frequency (1/3 weight)^17^.

#### Inter-quantile range (IQR) filter for Google search data

Instability and sudden spikes/drops in Google search volume data can due to natural noises in Google Trends’ sampling approach. Meanwhile, sparsity might still exist in some states’ search queries after regional enrichment of state-level Google search data. Such instability and sparsity severely reduce the prediction accuracy at the national, regional, and state level. Therefore, we introduce an Inter-Quantile Range (IQR) based data filtering mechanism to reduce the noise in the data.

We first drop the Google search terms that have frequencies lower than the median number of all other Google search queries frequencies. Then, we identify the outliers of a Google search query. The large-valued outliers are those above 99.9 percent quantile and three standard deviations above past-week rolling average. The small-value outliers are those below 1 percent quantile. We overwrite the outlier values to be the past three-day average.

The reason behind different data processing approaches for large and small valued outliers lies in the hypothesis that a sudden increase of search volume is more probable to be true than a drop in search frequency of a Google search query, which is possibly due to inadequate search intensity. For instance, sudden increases in search frequencies of COVID-19 related terms occur when COVID-19 first hit the U.S. in mid-March 2020, while decreases/sparsity in search query volumes are resulted from low search volume and missing data, especially in state-level search queries. Therefore, the IQR filter is “looser” on small-valued outliers and “stricter” on large-valued outliers. As a result, we believe that a large-valued outlier is indeed an “unreasonable” spike if they are significantly larger than search frequencies from the other days in the same week.

This IQR inspired filtering mechanism can further account for the sparsity in Google search queries as well as removing unusual spikes, which improves our model’s forecast accuracy.

#### Optimal lag

**Figure 1:**
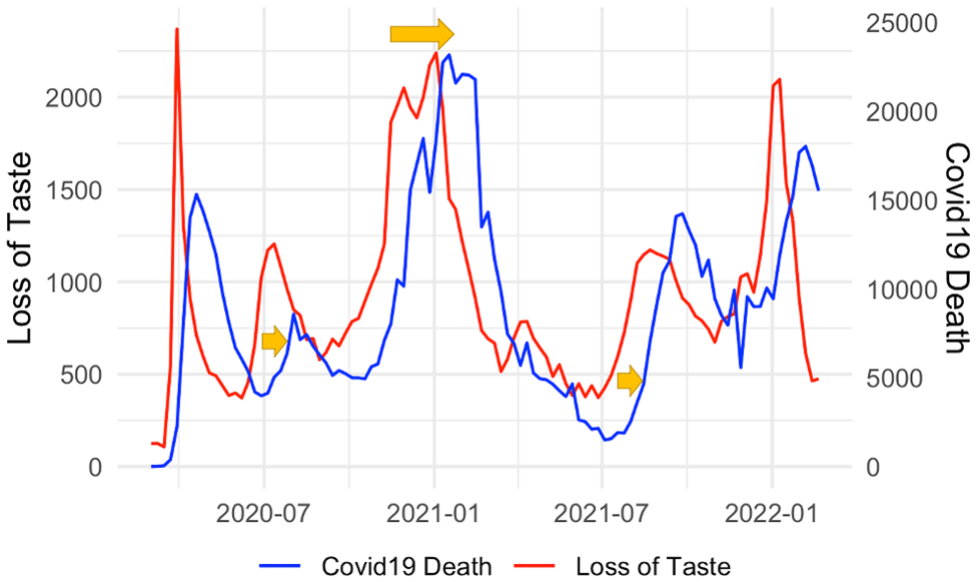
Google search query “loss of taste” and COVID-19 weekly incremental death illustration of delay in peak between Google search query search frequencies (Loss of Taste in red) and COVID-19 national level weekly incremental death (blue). Y-axis are adjusted accordingly.

It is typical to see the peak of COVID-19 search volume ahead of the peak in reported cases or deaths, see Fig. 1 for an illustration for query “loss of taste”. One hypothesis is that the early-stage infected people could search for COVID-19 related information online before their arrival at a clinic or tested positive.

As such, using delayed Google search frequencies for forecast is essential for our predictions. One simple way is to enumerate all possible delaying lags of Google search frequencies as exogenous feature variables. Yet, this will significantly increase the number of exogenous variables and impact prediction accuracy which could result in over-fitting. Thus, we derive the optimal lag for each Google search query and only consider those optimal lags in forecasting model.

We use the period from April 1st, 2020, to June 30th, 2020, to find the optimal lags. We will use the period after July 1st, 2020, for comparing forecast accuracy. Because media-driven or information-seeking searches are very common at the beginning of the pandemic^40,41^, we exclude period prior to April 1st, 2020, in the analysis, so that the query terms identified are more likely to be driven by actual infection. For each query, we fit a linear regression of COVID-19 daily death against lagged Google search frequency, considering a range of lags (4 to 35 days). We select the lagged Google search frequency that has the lowest mean square error (MSE) as the optimal lag for that query. Table S3 (in Supplementary Materials) shows all the optimal lags for the selected important Google search terms, ranked by their optimal lags. For national, regional and state level Google search information, we consider the same optimal lag throughout this study.

#### Highly correlated 23 terms

Though we removed low frequency queries and sparsity in the remaining queries through the IQR filter, some remaining queries might still exhibit high variability and do not obtain a clear trend comparing to COVID-19 death. To further obtain most useful terms for predicting COVID-19 death and eventually reduce our model complexity, we computed Pearson correlation coefficient between each of the optimal lagged search term and COVID-19 daily death during the period from April 1st, 2020, to June 30th, 2020, where the detail derivation of optimal delay is shown in previous section. All the Google search query terms and their Pearson correlation coefficient against COVID-19 daily death are listed in Table S2, in which only positive Pearson correlation terms are displayed. We select the 23 terms that have Pearson correlations above 0.5 as “important terms” and only use them in our forecast model, shown in Table S1.

### Forecasting models

**Figure 2:**
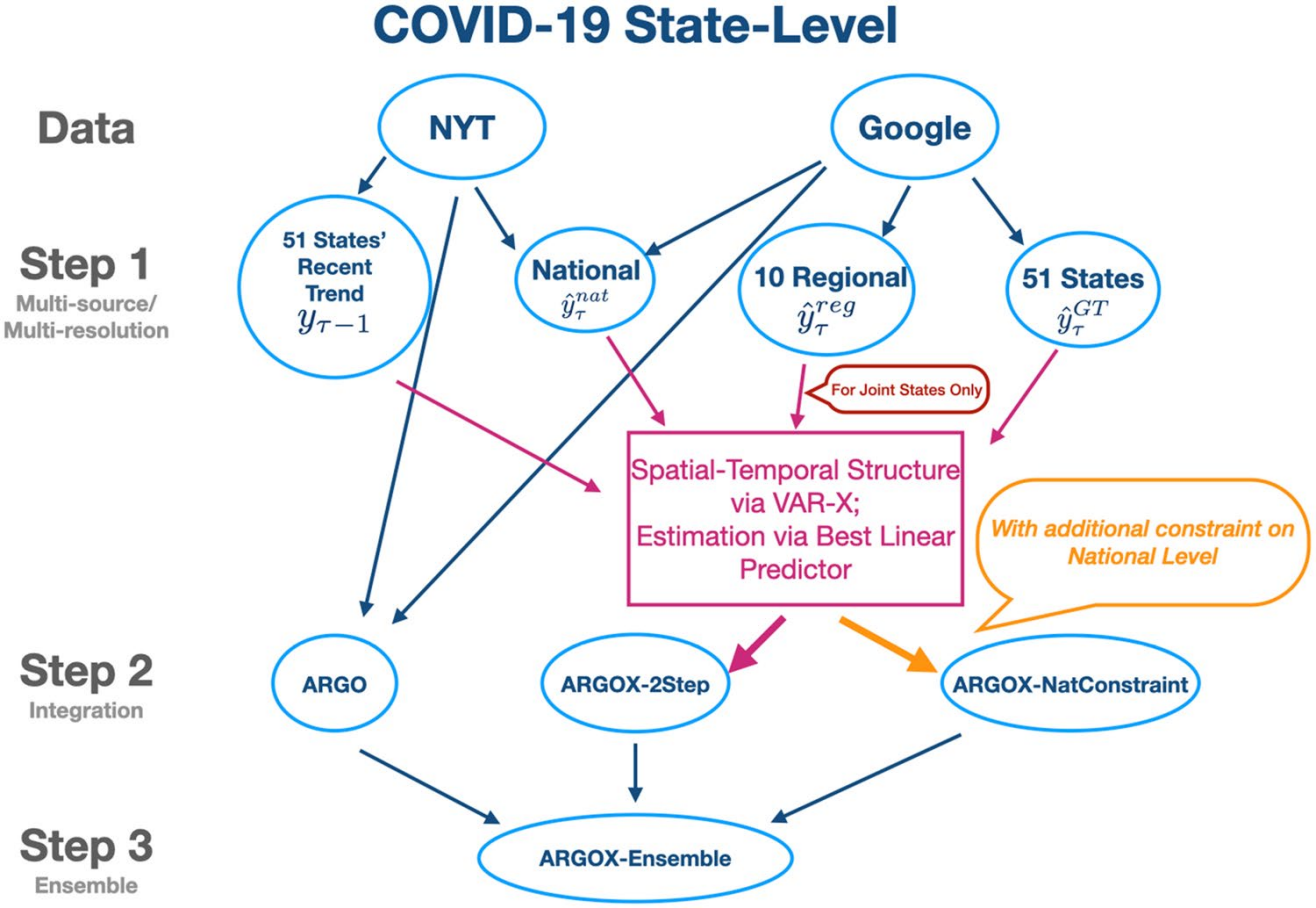
COVID-19 State-Level forecasting methods illustration in flowchart, operated in top-to-bottom fashion. Level 1 shows input data sources being NYT (COVID-19 cases/deaths) and Google search information. Level 2 (step 1) shows different estimations for state-level deaths at week $\uptau$. Level 3 (step 2) shows 3 proposed state-level death forecasting methods, each incorporates different information in level 1 and level 2 as inputs. Level 4 (step 3) shows the final state-level forecasting model, ARGOX-Ensemble, which combines the 3 methods in step 2 via winner-takes-all ensemble. For details please see the Methods section, including the VAR-X (vector-autoregressive with exogenous variables) structure.

In this section, we present our proposed national and state-level COVID-19 deaths’ forecasting models in detail. In particular, the national-level forecasts operate in one step, utilizing relevant Google search queries and COVID-19 cases/death information in a *L*_1_ regularized linear model (presented in “ARGO inspired prediction” section below), while the state-level forecasts operate in three steps by further incorporating cross-state, cross-regional spatial–temporal information in an ensemble framework. A high-level illustration of the state-level forecasting framework is shown in Fig. 2, while the following subsections will present the framework in a step-by-step breakdown fashion.

#### ARGO inspired prediction

Let ${X}_{i,t,m}$ be the Google Trends data of search term $i$ day $t$ of area $m$; ${y}_{t,m}$ be the New York Times COVID-19 death increment at day $t$ of area $m$; ${c}_{t,m}$ be the New York Times COVID-19 confirmed case increment at day $t$ of area $m$; where the area $m$ can refer to the entire nation, one specific HHS region (such as New England), or one specific state (such as Georgia). Let ${O}_{k}$ be the optimal lag for the $k$th Google search term, which is the same for all area $m$. Let ${\mathbb{I}}_{\{t,r\}}$ be the weekday $r$ indicator for $t$ (i.e., ${\mathbb{I}}_{\{t,1\}}$ indicates day $t$ being Monday, and ${\mathbb{I}}_{\{t,6\}}$ indicates day _*t*_ being Saturday), which accounts for the weekday seasonality in COVID-19 incremental death time series.

Inspired by ARGO method^12^, with information available as of time $T$, to estimate ${y}_{T+l,m}$ for $l>0$, the incremental COVID-19 death on day $\left(T+l\right)$ of area $m$, an ${L}_{1}$ regularized linear estimator is used:

1 $${\widehat{y}}_{T+l,m}={\widehat{\mu }}_{y,m}+{\sum }_{i=0}^{I}{\widehat{\alpha }}_{i,m}{y}_{T-i,m}+\sum_{j\in \mathcal{J}}{\widehat{\beta }}_{j,m}{c}_{T+l-j,m}+\sum_{k=1}^{K}{\widehat{\delta }}_{k,m}{X}_{k,T+l-{\widehat{O}}_{k},m}+{\sum }_{r=1}^{6}{\widehat{\gamma }}_{r,m}{\mathbb{I}}_{\{T+l,r\}}$$

where we use lagged death, lagged confirmed cases and optimal lagged Google search terms for death prediction.For $l$th day ahead prediction at area $m$, the coefficients $\{{\mu }_{y,m},\boldsymbol{\alpha }=\left({\alpha }_{1,m},\dots ,{\alpha }_{I,m}\right),{\varvec{\beta}}=\left({\beta }_{1,m},\dots ,{\beta }_{\left|\mathcal{J}\right|,m}\right),{\varvec{\delta}}=\left({\delta }_{1,m},\dots ,{\delta }_{K,m}\right),{\varvec{\gamma}}=\left({\gamma }_{1,m},\dots ,{\gamma }_{6,m}\right)\}$ are obtained via

2 $$\underset{{\mu}_{\text{y,m}}, \boldsymbol{\alpha},\boldsymbol{\beta}, \boldsymbol{\delta}, \boldsymbol{\gamma}, \boldsymbol{\lambda}}{\mathrm{argmax}} \sum_{t=T-M-l+1}^{T-l}{\left({y}_{t+l,m}-{\mu }_{y,m}-{\sum }_{i=0}^{6}{\alpha }_{i,m}{y}_{t-i,m}-\sum_{j\in \mathcal{J}}{\beta }_{j,m}{c}_{t+l-j,m}-\sum_{k=1}^{23}{\delta }_{k,m}{X}_{k,t+l-{\widehat{O}}_{k,m}}-\sum_{r=1}^{6}{\gamma }_{r,m}{\mathbb{I}}_{\{t+l,r\}}\right)}^{2}+{\lambda }_{\alpha }{\left||\boldsymbol{\alpha }\right||}_{1}+{\lambda }_{\beta }|{\left|{\varvec{\beta}}\right||}_{1}+{\lambda }_{\delta }|{\left|{\varvec{\delta}}\right||}_{1}+{\lambda }_{\gamma }|{\left|{\varvec{\gamma}}\right||}_{1}$$

We set $M=56$, i.e. 56 days as training period; $I=6$ considering consecutive 1 week lagged death; $\mathcal{J}=\mathrm{max}\left(\{\mathrm{7,14,21,28}\},l\right)$ considering weekly lagged confirmed cases; $K=23$ highly correlated Google search terms; ${\widehat{O}}_{k}=\mathrm{max}\left({O}_{k},l\right)$ be the adjusted optimal lag of $k$th Google search term subject to $l$th day ahead prediction. We set hyperparameters ${\varvec{\lambda}}=\left({\lambda }_{\alpha },{\lambda }_{\beta },{\lambda }_{\delta },{\lambda }_{\gamma }\right)$ through cross-validation. For simplicity, we constrain ${\lambda }_{\alpha }={\lambda }_{\beta }={\lambda }_{\delta }={\lambda }_{\gamma }$*.*

To further impose smoothness into our predictions, we use the three-day moving average of the coefficients for predicting day $\left(\mathrm{T}+\mathrm{l}\right)$, which slightly boosts our prediction accuracy. Using the above formulation, we forecast future 4 weeks of daily incremental COVID-19 death of area *m*, i.e. $\{{\widehat{y}}_{T+1,m},\dots ,{\widehat{y}}_{T+28,m}\}$, and aggregate them into weekly prediction. In other words, ${\widehat{y}}_{T+1:T+7,m}={\sum }_{i=1}^{7}{\widehat{y}}_{T+i,m}$ is first week, ${\widehat{y}}_{T+8:T+14,m}={\sum }_{i=8}^{14}{\widehat{y}}_{T+i,m}$ is the second week, ${\widehat{y}}_{T+15:T+21,m}={\sum }_{i=15}^{21}{\widehat{y}}_{T+i,m}$ is the third week, and ${\widehat{y}}_{T+22:T+28,m}={\sum }_{i=22}^{28}{\widehat{y}}_{T+i,m}$ is the fourth week ARGO incremental death prediction. We denote this method as “ARGO inspired prediction”. This method will also serve as one of the three ensembled methods in state-level forecasts, denoted as “ARGO” in step 3 in Fig. 2.

#### ARGOX inspired state level prediction

In the previous section, our prediction is based on Google search terms and past COVID-19 cases and death information. The predictions perform competitively on national level, but fall short in regional and state level due to the spatial correlation induced by geographical proximity, transportation connectivity and region-state-wise related spread. Thus, we incorporate ARGOX^17^, a unified spatial–temporal statistical framework that combines multi-resolution, multi-source information while maintaining coherency with regional and national COVID-19 death. ARGOX^17^ operates in two steps: the first step extracts state level internet search information via LASSO, and the second step enhances the estimates using cross-region, cross-resolution spatial temporal framework (see Fig. 2).

In step 1 (Fig. 2), our raw estimates for the 51 state-level weekly COVID-19 incremental death ${{\varvec{y}}}_{\tau }=\left({y}_{\tau ,1},\dots ,{y}_{\tau ,51}\right)$ are ${\widehat{{\varvec{y}}}}_{\tau }^{GT}={\left({\widehat{y}}_{\tau ,1}^{GT},\dots ,{\widehat{y}}_{\tau ,51}^{GT}\right)}^{\intercal }$, ${\widehat{{\varvec{y}}}}_{\tau }^{reg}={\left({\widehat{y}}_{\tau ,{r}_{1}}^{reg},\dots ,{\widehat{y}}_{\tau ,{r}_{51}}^{reg}\right)}^{\intercal }$, and ${\widehat{{\varvec{y}}}}_{\tau }^{nat}={\left({\widehat{y}}_{\tau ,1}^{nat},\dots ,{\widehat{y}}_{\tau ,51}^{nat}\right)}^{\intercal }$, where ${r}_{m}$ is the region number for state $m$. Here, we denote $GT$ and $reg$ to be state/regional estimates from Eq. 4 with internet search information only (see Supplementary Materials), and $nat$ to be national estimates from Eq. (1) above. We denote the state-level death increment at week $\uptau$ as ${{\varvec{Z}}}_{\tau }=\Delta {{\varvec{y}}}_{\tau }={{\varvec{y}}}_{\tau }-{{\varvec{y}}}_{\tau -1}$ and it has four predictors: (i) ${{\varvec{Z}}}_{\tau -1}=\Delta {{\varvec{y}}}_{\tau -1}$*,* (ii) ${\widehat{{\varvec{y}}}}_{\tau }^{GT}-{{\varvec{y}}}_{\tau -1}$*,* (iii) ${\widehat{{\varvec{y}}}}_{\tau }^{reg}-{{\varvec{y}}}_{\tau -1}$*,* and (iv) ${\widehat{{\varvec{y}}}}_{\tau }^{nat}-{{\varvec{y}}}_{\uptau -1}$. Let ${W}_{\uptau }$ denote the collection of these four vectors ${{\varvec{W}}}_{\tau }={\left({{\varvec{Z}}}_{\tau -1}^{\intercal },{\left({\widehat{{\varvec{y}}}}_{\tau }^{GT}-{{\varvec{y}}}_{\tau -1}\right)}^{\intercal },{\left({\widehat{{\varvec{y}}}}_{\tau }^{reg}-{{\varvec{y}}}_{\tau -1}\right)}^{\intercal },{\left({\widehat{{\varvec{y}}}}_{\tau }^{nat}-{{\varvec{y}}}_{\tau -1}\right)}^{\intercal }\right)}^{\intercal }$. Effectively, we are predicting ${{\varvec{Z}}}_{\tau }$ from ${{\varvec{W}}}_{\tau }$ using a VAR-X structure (vector autoregressive with exogenous variables), in which the previous week’s state-level death increment ${{\varvec{Z}}}_{\tau -1}$ is the lagged 1 autoregressive term, and the rest are three exogenous variables incorporating Google search information at different geographical resolutions (shown in the intermediate red box in Fig. 2).

The second step takes a dichotomous approach for the joint states and alone states, identified through geographical separations and multiple correlations on COVID-19 growth trends. We incorporate the cross-state, cross-source correlations for the joint model of connected states. In particular, we use penalized best-linear-predictor^17^ to estimate the ${{\varvec{Z}}}_{\tau }$ from ${{\varvec{W}}}_{\tau }$, and obtain the COVID-19 incremental death predictions for all states in week $\uptau$ jointly. On the other hand, we conduct stand-alone modeling for the isolated states. Specifically, we construct the predictors in for the alone state $m$ as ${W}_{\tau ,m}={\left({Z}_{\tau -1,m}^{\intercal },{\left({\widehat{y}}_{\tau ,m}^{GT}-{y}_{\tau -1,m}\right)}^{\intercal },{\left({\widehat{y}}_{\tau ,m}^{nat}-{y}_{\tau -1,m}\right)}^{\intercal }\right)}^{\intercal }$ by dropping the regional-level estimates in the VAR-X structure due to their isolation in COVID-19 deaths trend. Again, we use penalized best-linear-predictor^17^ to estimate the coefficients and predict ${Z}_{\tau ,m}$ from ${W}_{\tau ,m}$, and obtain the incremental death predictions for week $\uptau$ separately for each alone state.

All detailed derivations for step 1 and step 2 are shown in Supplementary Materials. We denote this method as “ARGOX-2Step”.

#### State level forecasting with national constraint

Although ARGOX is a consistent framework in cascading fashion, the exact predictions from ARGOX are not consistent through aggregation from bottom up. There exists inconsistency between the aggregation of state-level ARGOX prediction and national level prediction ARGO prediction, illustrated in Supplementary Materials (Figure S3, S4), which shows that the sum of ARGOX 51 states’ COVID-19 incremental death predictions does not equal the national level ARGO prediction. Furthermore, our ARGO inspired national level predictions are more accurate for the national level ground-truth than the sum of ARGOX 51 states’ predictions. Therefore, we propose a constrained second step to ARGOX inspired state level prediction, by restricting the sum of state-level ARGOX predictions to be close to the ARGO inspired national prediction.

After the first step in the previous section, instead of separating the 51 US states into “joint” and “alone” states and estimating state-level COVID-19 deaths separately, we treat them as a whole (except HI and VT) as we are constraining the sum of all states’ death estimations. We separate out HI and VT, since these two states have the lowest incremental COVID-19 deaths, and such sparsity causes instability when deriving the covariance matrices. We estimate HI and VI COVID-19 weekly incremental death using the ARGO inspired state-level estimates through Eq. (1).

We obtain the same four raw estimates for 49 state-level weekly COVID-19 incremental deaths (except HI and VT) as in the prior section, and collect the four raw estimates into the VAR-X structure vector, ${{\varvec{W}}}_{\tau }$.

We denote ${\widehat{y}}_{\tau ,nat}^{*\mathrm{ARGO}}$ as the week $\uptau$ national level ARGO-inspired COVID-19 incremental death estimation excluding HI and VT. To predict the week $\tau$ state-level COVID-19 death increment, we solve the constrained optimization problem below that minimizes the variance between ground-truth and our predictor, subject to the constraint that the sum of our predictor ought to be close to national level COVID-19 incremental death estimations.

$$\underset{{\varvec{A}}}{\mathrm{min }}{\text{Tr}}\left({\text{Var}}\left({{\varvec{Z}}}_{\uptau }-{\varvec{A}}{{\varvec{W}}}_{\uptau }\right)\right)$$

3 $$\text{s.t.} {1}^{\intercal }\left({\mu }_{Z}+{\varvec{A}}{{\varvec{W}}}_{\uptau }\right)={\widehat{y}}_{\tau ,nat}^{*\mathrm{ARGO}}-{1}^{\intercal }{{\varvec{y}}}_{\uptau -1}$$

where $1={\left(1,\dots ,1\right)}^{\intercal }$ is a length 49 vector of 1 s, and ${\mu }_{Z}$ is the mean of ${{\varvec{Z}}}_{{\varvec{\uptau}}}$. In particular if the above optimization problem is unconstrained, the solution will be the best-linear predictor (no ridge-penalty) in ARGOX^17^. The detail derivation of the optimization problem in Eq. (3) as well as the final closed-form solution can be found in the (Supplementary Materials). We denote this method as “ARGOX-NatConstraint”.

#### Winner-takes-all state level ensemble forecast

To further boost the state-level COVID-19 death prediction accuracy, we incorporate an ensemble framework that combines our previous estimations and selects the best predictor for each week (see step 3 in Fig. 2). For all 51 U.S. states, we denote the ARGO-inspired state-level prediction for week $\uptau$ as “ARGO”, ARGOX-inspired joint-alone state prediction as “ARGOX-2step”, and ARGOX-inspired national constrained prediction as “ARGOX-NatConstraint”. For a training period of 15 weeks, we evaluate each predictor with mean squared error (MSE) and select the one with lowest MSE as the ensemble predictor for week $\left(\tau +1\right)$, $(\tau +2)$, $(\tau +3)$, and $(\tau +4)$. Such winner-takes-all approach has been previously shown to be effective for influenza estimation^14^.

## Results

### Evaluation metrics

We use three metrics to evaluate the accuracy of an estimate of COVID-19 death against the actual COVID-19 Death published by John Hopkins University (JHU): the root mean squared error (RMSE), the mean absolute error (MAE), and the Pearson correlation (Correlation). RMSE between an estimate $\widehat{{y}_{t}}$ and the true value ${y}_{t}$ over period $t=1,\dots , T$ is $\sqrt{\frac{1}{T}{\sum }_{t=1}^{T}{\left(\widehat{{y}_{t}}-{y}_{t}\right)}^{2}}$. MAE between an estimate $\widehat{{y}_{t}}$ and the true value ${y}_{t}$ over period $t=1,\dots , T$ is $\frac{1}{T}{\sum }_{t=1}^{T}\left|\widehat{{y}_{t}}-{y}_{t}\right|$. Correlation is the Pearson correlation coefficient between $\widehat{y}=\left(\widehat{{y}_{1}},\dots ,\widehat{{y}_{T}}\right)$ and ${\varvec{y}}=\left({y}_{1},\dots ,{y}_{T}\right)$. We first conduct retrospective evaluation against variations of our methods, and then compare against CDC published official forecasts^8,11,42–45^.

### Comparison among our methods

#### National level

We focus on United State’s 51 states/districts (including Washington DC) for all comparisons in this section. In this section, we conduct sensitivity analysis by comparing our own methods on national level and state level for 1 to 4 weeks ahead COVID-19 incremental death prediction.

For national level, we compare with four other simplified models: (1) persistence (Naive), (2) ARGO inspired prediction, (3) AR-only prediction, and (4) GT-only prediction. The Naive (persistence) predictions use current week’s incremental death counts from New York Times (NYT) as next 1 to 4 weeks’ estimation, since NYT does not retrospectively correct past data. AR-only prediction uses the model setup in section “ARGO inspired prediction” but only with lagged death and cases information, whereas GT-only prediction is using Google search information only. For fair comparisons, both AR-only and GT-only predictions include weekday indicators and use 56 days training period. Daily COVID-19 incremental deaths are estimated using the three methods above and aggregated into weekly incremental deaths for the time period of July 4, 2020, to Mar 5th, 2022. The period before July 4, 2020, is excluded for comparison analysis, as the optimal Google search terms’ lags are selected using that period. The ground-truth is COVID-19 weekly incremental death from JHU COVID-19 dataset.

**Figure 3:**
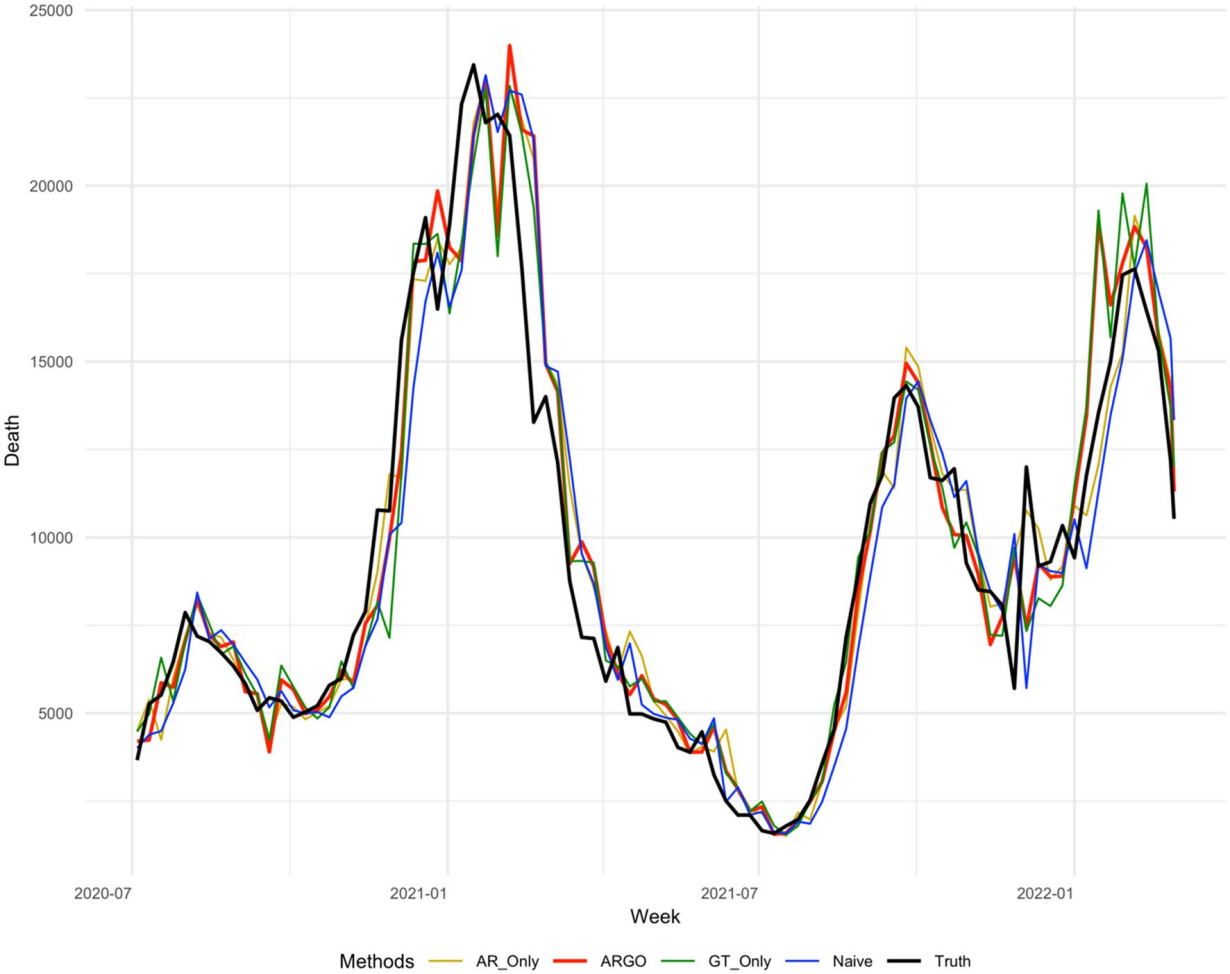
1 week ahead national level COVID-19 weekly incremental death predictions’ comparisons weekly from 2020-07-04 to 2022-03-05. The method included are AR-Only, ARGO, GT-Only, Naive (persistence), truth. ARGO estimations (thick red), contrasting with the true COVID-19 death from JHU dataset (thick black) as well as the estimates from AR-Only (gold), GT-Only (Green), and Naive (blue).

**Figure 4:**
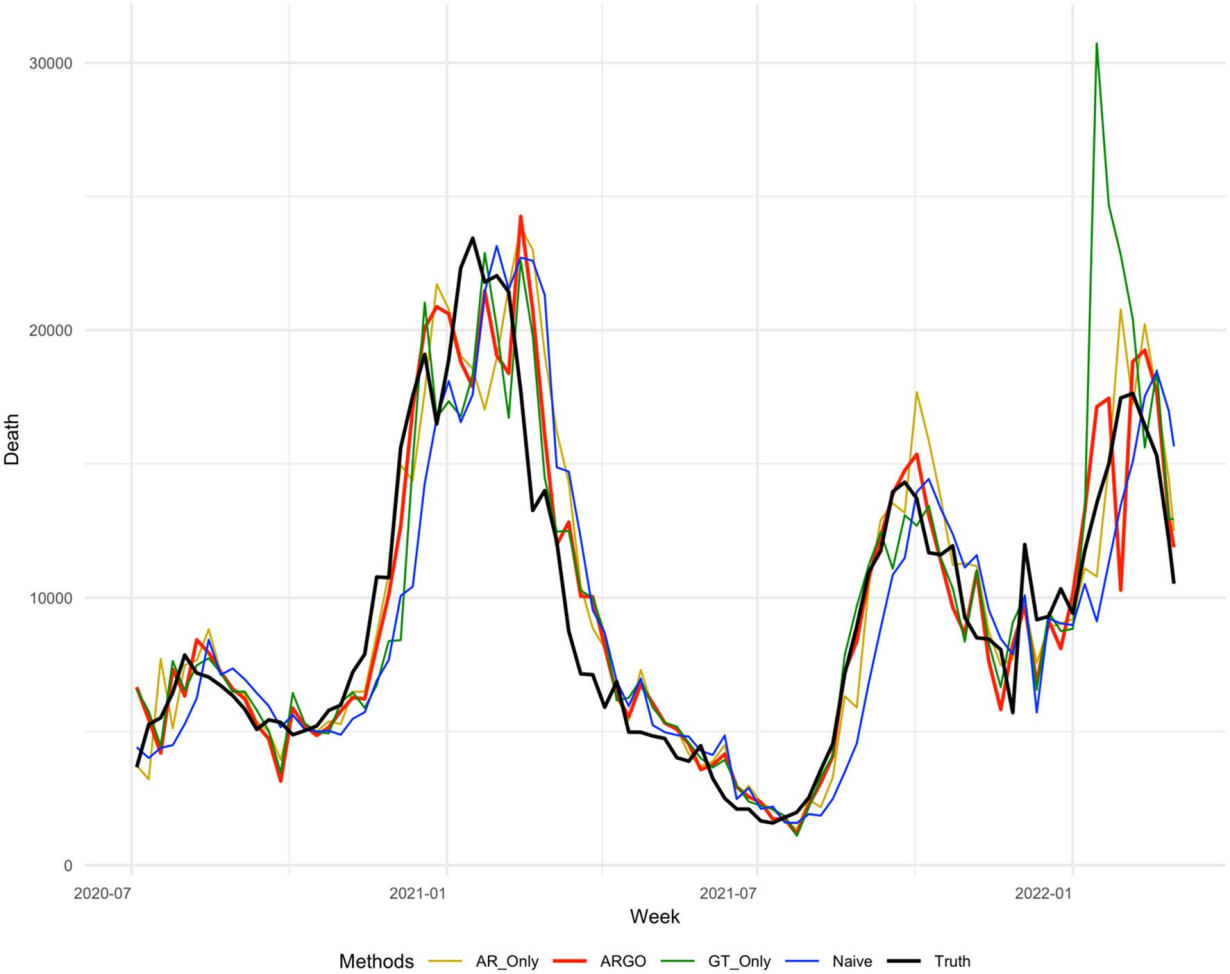
2 weeks ahead national level COVID-19 weekly incremental death predictions’ comparisons weekly from 2020-07-04 to 2022-03-05. The method included are AR-Only (gold), ARGO (thick red), GT-Only (Green), Naive (blue), truth (thick black).

**Figure 5:**
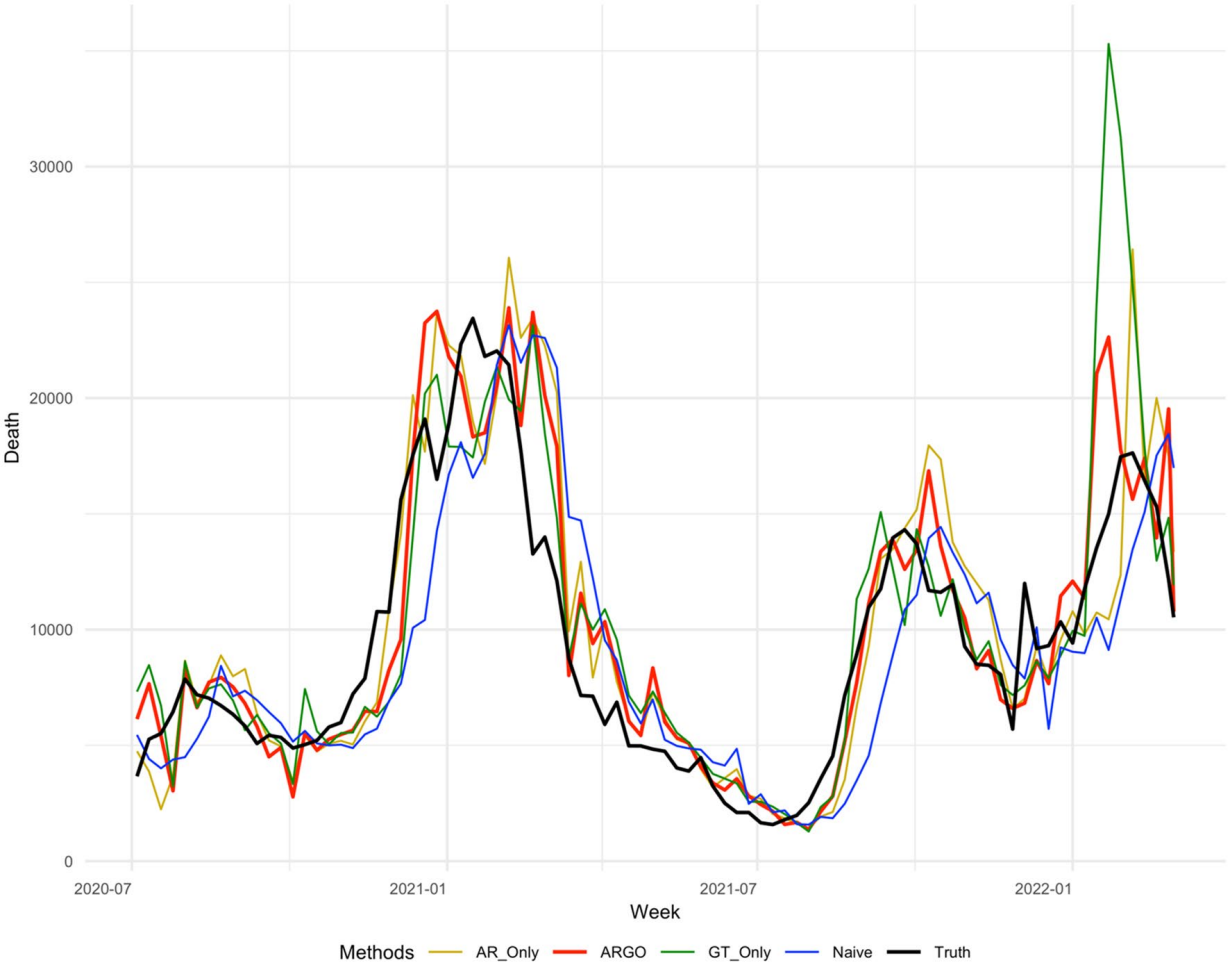
3 weeks ahead national level COVID-19 weekly incremental death predictions’ comparisons weekly from 2020-07-04 to 2022-03-05. The method included are AR-Only (gold), ARGO (thick red), GT-Only (Green), Naive (blue), truth (thick black).

**Figure 6:**
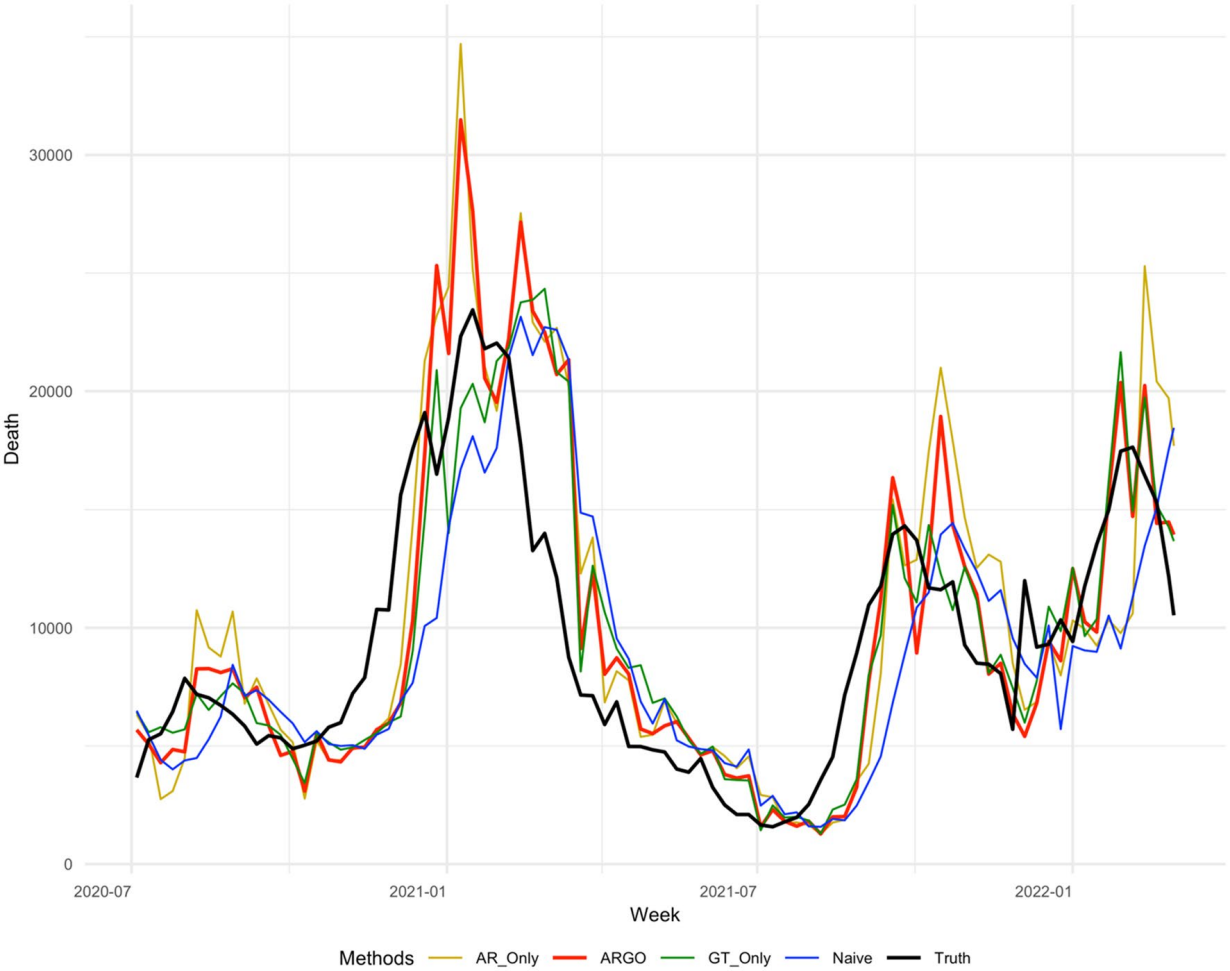
4 weeks ahead national level COVID-19 weekly incremental death predictions’ comparisons weekly from 2020-07-04 to 2022-03-05. The method included are AR-Only (gold), ARGO (thick red), GT-Only (Green), Naive (blue), truth (thick black).

Table 1 summarizes the accuracy metrics for all estimation methods for the period from July 4, 2020, to Mar 5th, 2022 on national level, which shows that ARGO’s estimates outperform all other simplified models, in every accuracy metric for the whole time period. Figures 3, 4, 5, 6 displays the estimates against the observed COVID-19 1–4 weeks ahead incremental deaths.

**Table 1:** National level 1 to 4 weeks ahead COVID-19 incremental prediction comparisons in 3 error metrics.

Methods	1 Week ahead	2 Weeks ahead	3 Weeks ahead	4 Weeks ahead
**RMSE**				
AR Only	1797.77	2392.77	3215.94	4612.05
ARGO	*1779.70*	*2182.81*	*2905.77*	*3610.10*
GT Only	1845.55	3040.13	3868.83	3663.73
Naïve	2069.72	2901.27	3773.09	4577.69
**MAE**				
AR Only	1177.34	1699.50	2351.12	3599.44
ARGO	*1167.59*	*1553.64*	*1997.67*	*2609.60*
GT Only	1336.93	1871.51	2394.29	2636.63
Naive	1461.17	2144.36	2945.75	3601.64
**Correlation**				
AR Only	0.95	0.91	0.87	0.79
ARGO	*0.95*	*0.92*	*0.89*	*0.81*
GT Only	0.94	0.86	0.80	0.73
Naive	0.93	0.86	0.77	0.65

The best performances for each metric in each study period are italicized. All comparisons are based on the original scale of COVID-19 national incremental death. On average, ARGO is able to achieve around 22% RMSE, 27% MAE reduction, and 9% correlation improvement, compared to the Naïve predictions.

ARGO outperforms simplified models in terms of RMSE, MAE, and Pearson correlation throughout 1 to 4 weeks ahead predictions. AR-Only predictions, on the other hand, have higher MSE comparing against Naive predictions for 4 weeks ahead, suggesting the importance of Google search terms in the method. COVID-19 death’s trend is highly correlated with the optimal lagged important terms we selected, which boosts the model’s accuracy. However, using only Google search information isn’t good enough as well, as GT-only predictions are barely beating Naive predictions for 1 to 4 weeks ahead considering MAE and fall behind if considering RMSE, indicating autoregressive information (lagged COVID-19 cases and death) can help predictions using solely Google search terms by correcting its trend to not overshoot or underestimate, as shown in the period between December 2020 to March 2021, and between December 2021 to March 2022 in Figs. 3, 4, 5, 6. Delaying behavior exists in all methods for 1 to 4 weeks ahead predictions, due to the lagged latest information to train for predictions, especially when forecast horizon extends to 3 and 4 weeks ahead. Yet, utilizing people’s search behavior to foresee future trends, ARGO is able to overcome such delay effect in almost all the weeks for 1 week ahead predictions and majority of the weeks in 2020 for 2 to 4 weeks ahead predictions. Also, ARGO is the only method that captures the three COVID-19 death peaks in January 2021, September 2022, and January 2022, while remains its accuracy in the latest Omicron-Variant surges, for all 1 to 4 weeks ahead predictions. The integration of time series information and Google search terms leads to a trend-capturing estimation curve without undesired spikes in 1 to 3 weeks ahead forecasts, and robust recovering of spikes in 4 weeks ahead forecasts comparing to other benchmark methods. The results presented above demonstrate ARGO’s accuracy and robustness. The optimal lagged 23 important Google search terms appear to be key factors in the enhanced accuracy of ARGO, as well as the past week’s COVID-19 deaths, as shown in Supplementary Materials (Figure S5, S6), which reflect a strong temporal autocorrelation after the feature selection of ${L}_{1}$ penalty.

“ARGO inspired prediction” accounts for the weekday seasonality in COVID-19 cases and deaths reporting by using a weekday indicator, which might have incapability when there exist extreme irregularities in the COVID-19 reporting. Thus, we conduct a sensitivity analysis for our “ARGO-Inspired” model towards weekday seasonality effect by comparing it against the naive method (uses last-week's prediction as current week's) and “ARGO-Smooth”, which incorporates 7-day smoothed COVID-19 cases, deaths, and important Google search queries' frequencies as inputs. The comparison is conducted over future 1–4 weeks ahead national level COVID-19 incremental death predictions, and is presented in the Supplementary Materials. Figure S1 displays the forecasting trends of the three comparing methods while Table S4 shows the forecasting performances in different error metrics. “ARGO-Inspired Predictions” is able to account for sudden changes in COVID-19 death trends better than “ARGO-Smooth”, indicating smoothing the outputs leads to more robust forecasts than smoothing the inputs.

We also conduct an additional sensitivity study where we include new hospital admissions (hospitalizations) as one additional predictor towards COVID-19 deaths. Specifically, we evaluate four models for national COVID-19 deaths predictions: (1) persistence (Naive), (2) GT + AR + Hosp prediction, (3) GT + AR prediction, (4) AR + Hosp prediction, where we denote the selected Google search queries as GT, COVID-19 cases/deaths as AR, and hospitalizations as Hosp. Here, hospitalization is obtained from U.S. Department of Health and Human Services (HHS)^46^ for the period from 2020-11-06 to 2022-03-05. The comparison is conducted over future 1–4 weeks ahead national level COVID-19 deaths predictions, and is presented in Supplementary Materials. Table S5 shows the forecasting performance, and Figure S2 displays all methods' forecasting trends. In summary, the combination of AR + Hosp is less accurate than GT + AR + Hosp or GT + AR for 1–4 weeks ahead COVID-19 death predictions, indicating the additional predictive power of Google search on top of hospitalizations. The hospitalizations information is also a short-term signal, and the predictive power deteriorates much faster than Google search as the prediction horizon goes to 4 weeks into the future, possibly due the short lag between hospital admission and death. Nevertheless, hospitalizations can serve as an additional feature to Google search queries and COVID-19 cases/deaths information, and further assist ARGO-Inspired (GT + AR) predictions. However, due to state-level data availability, we do not include hospitalization in our main analysis.

#### State level

For state level, we compare persistence (Naive) predictions, ARGO prediction, ARGOX-2step prediction, ARGOX-NatConstraint and Winner-takes-all Ensemble. ARGO predictions for each state are obtained from a ${L}_{1}$ penalized regression, utilizing the lagged state-level COVID-19 cases, deaths and optimal lagged Google search terms. ARGOX-2step combines cross-state cross-region information to obtain model outputs as predictions. ARGOX-NatConstraint, revised upon ARGOX-2step, constrains the sum of state-level prediction to align with national-level prediction while pooling cross-resolution cross-temporal information. ARGO daily predictions use 56 days as training period and are aggregated into 1 to 4 weeks ahead weekly incremental death predictions. ARGOX-2step, ARGOX-NatConstraint, and winner-takes-all ensemble method all use 15 consecutive and overlapping weeks as training period. We conduct retrospective estimation of the 1 to 4 weeks ahead 51 U.S. state level COVID-19 incremental deaths, for the period of July 4th, 2020, to Mar 5th, 2022. To evaluate the accuracy of our estimation, we conduct sensitivity analysis among our methods by comparing their estimations with the actual COVID-19 weekly incremental death released by JHU dataset in multiple error metrics, including RMSE, MAE, and the Pearson correlation.

**Figure 7:**
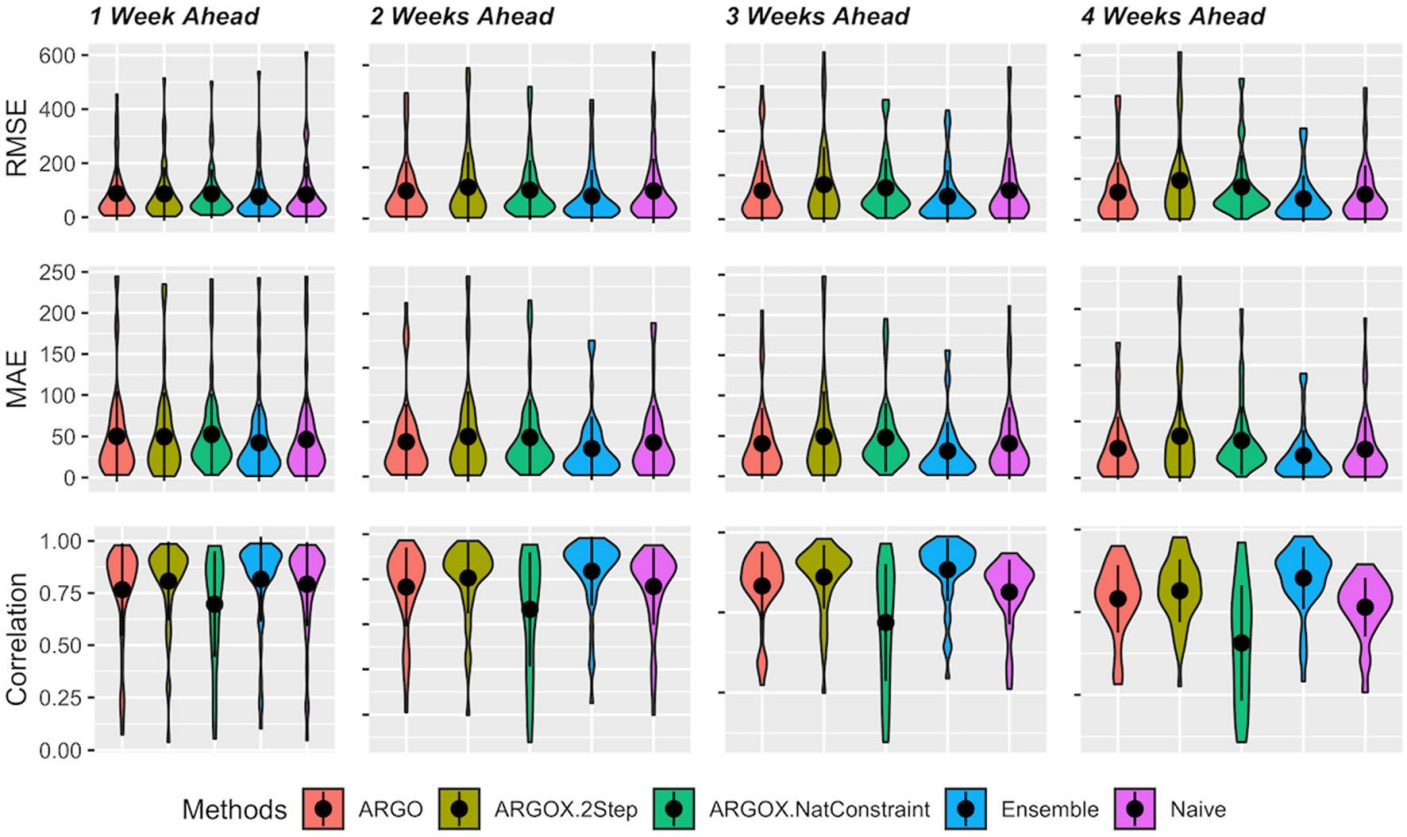
The distribution of values for each metric for each model, over the 51 states for 1 to 4 weeks (from left to right) ahead predictions during the period from 2020-07-04 to 2022-03-05. The embedded black dot and vertical line indicate mean and 1 standard deviation range. Average of each error metric across 51 states are reported in Table 2.

Table 2 summarizes the overall results of ARGO prediction, ARGOX-2step prediction, ARGOX-NatConstraint, Winner-takes-all Ensemble and Naive, averaging over the 51 states for the whole period of July 4th, 2020, to Mar 5th, 2022. Our winner-takes-all ensemble method gives the leading performance uniformly in all metrics as shown in, which achieves around 18% error reduction in RMSE, around 20% error reduction in MAE and around 8% increase in Pearson correlation compared to the best alternative in the whole period on average across 1 to 4 weeks ahead predictions. Among all the methods we compare in this section, winner-takes-all ensemble is the only method that uniformly outperforms the naive predictions. The robustness and accuracy are further illustrated in Fig. 7, which shows the 51 state’s RMSE, MSE and Pearson correlation in the violin charts for 1 to 4 weeks ahead predictions. The winner-takes-all ensemble approach outperforms all other approaches in the three metrics, in terms of mean and standard deviation range over all 51 states.

**Table 2:** Comparison of different methods for state-level COVID-19 1 to 4 weeks ahead incremental death in 51 U.S. states.

Methods	1 Week ahead	2 Weeks ahead	3 Weeks ahead	4 Weeks ahead
**RMSE**				
ARGO	99.57	122.37	139.27	182.02
ARGOX-2Step	95.49	126.77	164.85	254.32
ARGOX-NatConstraint	96.15	122.34	156.97	214.04
Ensemble	*85.03*	*97.31*	*108.15*	*135.33*
Naive	95.04	113.61	139.97	169.61
**MAE**				
ARGO	59.19	73.52	86.59	113.27
ARGOX-2Step	54.94	77.70	99.92	155.50
ARGOX-NatConstraint	57.82	78.28	100.65	141.42
Ensemble	*47.75*	*57.71*	*63.50*	*83.95*
Naive	51.65	68.30	86.54	110.24
**Correlation**				
ARGO	0.73	0.66	0.58	0.48
ARGOX-2Step	0.78	0.74	0.66	0.56
ARGOX-NatConstraint	0.68	0.55	0.42	0.34
Ensemble	*0.79*	*0.76*	*0.73*	*0.68*
Naive	0.75	0.69	0.59	0.49

The averaged RMSE, MAE, and correlation are reported and best performed method is italicized.

Detailed numerical results for each state are reported in Table S7–S57 and Figure S16-S66, in Supplementary Materials, where the ensemble approach demonstrates its accuracy in majority of the states. Notice that JHU ground-truth data exists jumps and spikes in some states due to retrospective edition. Such noisy ground-truth data is a key challenge to forecasting tasks. Our ensemble framework, on the other hand, reveals its robustness over geographical variability and extracts a strong combination from all other ARGO, ARGOX approaches.

To show detail break down of methods contributing to the ensemble approach, we display the ensemble approach’s selection proportion among ARGO, ARGOX-2step and ARGOX-NatConstraint 1 to 4 weeks ahead predictions for all 51 U.S. states from 2020-07-04 to 2022-03-05 (Table 3). Throughout the period for all 51 states, the ensemble approach selects state-level ARGO predictions the most (around 40%), and ARGOX-NatConstraint the least (around 25%) throughout 1 to 4 weeks ahead estimations. This is evident as Table 2 indicates state-level ARGO performs competitively against naive estimations for all 1 to 4 weeks ahead predictions on average across all the states for almost all three error metrics. Yet, state-level ARGO cannot beat naive predictions uniformly across all the states, as shown in Table S7–S57 and Figure S16–S66. On the other hand, though ARGOX-NatConstraint and ARGOX-2Step seem to perform poorly against naive predictions as shown in Table 2, they contribute to the ensemble approach and boost its accuracy drastically. Detailed ensemble approach state-level selections for 1 to 4 weeks ahead estimations are displayed in Figure S7 and S8. It seems that state-level ARGO, ARGOX-NatConstraint and ARGOX-2Step all have different strength and weakness depending on the particular state and time. Through adaptive selections of the best performer for each state, the winner-takes-all ensemble approach takes the best part of each of the three models, and thus achieves most robust prediction performance.

**Table 3:** Ensemble approach total selections across all 51 U.S. states for 1 to 4 weeks ahead predictions.

Methods	1 Week ahead	2 Weeks ahead	3 Weeks ahead	4 Weeks ahead
ARGO	0.39	0.40	0.42	0.44
ARGOX-2Step	0.35	0.35	0.32	0.31
ARGOX-NatConstraint	0.25	0.25	0.26	0.25

In addition to the weekly estimates, ARGOX Ensemble also gives confidence intervals, by simply taking the confidence intervals of selected method. Table S6 shows the coverage of the confidence intervals for all 51 states. The nominal 95% confidence interval has an actual 88.2% coverage on average for 1 week ahead predictions, suggesting that our confidence intervals reasonably measure the accuracy of our weekly estimates, albeit with over-confidence.

### Comparison with other publicly available methods

We obtain all available CDC published forecasts from teams making projections of COVID-19 cumulative and incident deaths for comparison^8,11,42–45^. CDC official predictions are a compilation of predictions from all teams that submit their weekly predictions every Monday since January 15th, 2020, contributed by different research groups or individuals. Note that all prediction models have their strengths in different date ranges and are providing robust and consistent predictions. We use both ARGOX Ensemble method and persistence (Naive) method for the comparison period from July 4th, 2020, to Mar 5th 2022 (29 prediction submission records). We only consider top 6 CDC published teams for prediction comparison, after filtering out teams having missing values in their reporting over the period and states we are considering, among over 100 teams submitted to CDC. Again, the ground-truth is COVID-19 weekly incremental death from JHU COVID-19 dataset, and naive approach uses this week’s death published by NYT as 1 to 4 weeks ahead predictions. We summarize the national level comparison results in Table 4 and the state level comparison results in Table 5, where we compared RMSE, MAE and Correlation of 1 to 4 weeks ahead national and state level COVID-19 death predictions. We rank the teams according to the average of each error metric we used. We further show the distribution of state level comparison results across all three error metrics in violin charts (Figure S9), where mean and standard deviations of each methods are displayed. Detailed state-by-state error metric are shown in Supplementary Materials (Figure S10–S15) as well.

**Table 4:** Comparison among different models’ 1 to 4 weeks ahead U.S. national level weekly incremental death predictions (from 2020-07-04 to 2022-03-05).

	1 Week ahead	2 Weeks ahead	3 Weeks ahead	4 Weeks ahead	Average
**RMSE**					
COVIDhub-ensemble^11^	1481.02	1811.27	2169.19	2538.15	1999.90
ARGOX Ensemble	1779.70 (#2)	2182.81 (#2)	2905.77 (#3)	3610.10 (#3)	2619.27 (#2)
LANL-GrowthRate^44^	1793.61	2326.31	2865.39	3976.25	2740.39
UA-EpiCovDA^45^	2184.63	2782.58	3268.05	3488.83	2931.02
MOBS-GLEAM COVID^43^	1975.80	2674.87	3318.10	3760.91	2932.42
epiforecasts-ensemble^8^	2266.51	2524.18	3082.81	4103.13	2994.16
Naive	2079.87	2901.27	3773.09	4577.69	3330.45
UMass-MechBayes^42^	1872.74	2885.64	4530.79	6965.55	4063.68
**MAE**					
COVIDhub-ensemble^11^	1048.83	1243.07	1517.51	1870.36	1419.94
ARGOX Ensemble	1167.59 (#2)	1553.64 (#2)	1997.67 (#2)	2609.60 (#2)	1831.62 (#2)
UMass-MechBayes^42^	1238.31	1633.54	2258.05	2968.95	2024.71
epiforecasts-ensemble^8^	1500.26	1725.81	2147.07	2871.49	2061.16
UA-EpiCovDA^45^	1533.67	1874.41	2243.16	2768.40	2104.91
LANL-GrowthRate^44^	1366.72	1855.02	2257.24	3071.18	2137.54
MOBS-GLEAM COVID^43^	1487.51	1954.44	2480.83	2797.97	2180.18
Naive	1461.17	2144.36	2945.75	3601.64	2537.75
**Correlation**					
COVIDhub-ensemble^11^	0.97	0.95	0.93	0.90	0.94
LANL-GrowthRate^44^	0.96	0.93	0.90	0.83	0.91
ARGOX Ensemble	0.95 (#3)	0.92 (#3)	0.89 (#3)	0.81 (#5)	0.89 (#3)
MOBS-GLEAM COVID^43^	0.95	0.91	0.86	0.82	0.89
UA-EpiCovDA^45^	0.94	0.90	0.85	0.84	0.88
epiforecasts-ensemble^8^	0.92	0.90	0.85	0.77	0.86
UMass-MechBayes^42^	0.94	0.88	0.79	0.71	0.83
Naive	0.93	0.87	0.77	0.66	0.81

The RMSE, MAE, Pearson correlation and their averages are reported. Methods are sorted based on their average. Our ARGOX-Ensemble’s rankings for each error metric are included in parenthesis.

**Table 5:** Comparison among different models’ 1 to 4 weeks ahead U.S. states level weekly incremental death predictions (from 2020-07-04 to 2022-03-05).

	1 Week ahead	2 Weeks ahead	3 Weeks ahead	4 Weeks ahead	Average
**RMSE**					
COVIDhub-ensemble^11^	73.81	83.10	93.28	105.43	88.90
ARGOX Ensemble	85.03 (#3)	97.31 (#2)	108.15 (#2)	135.33 (#4)	106.45 (#2)
MOBS-GLEAM COVID^43^	87.03	100.94	116.14	131.46	108.89
UA-EpiCovDA^45^	92.79	112.12	125.85	133.31	116.02
LANL-GrowthRate^44^	90.13	109.50	124.57	140.97	116.29
UMass-MechBayes^42^	79.95	100.25	129.12	172.84	120.54
Naive	95.04	113.61	139.97	169.61	128.10
epiforecasts-ensemble^8^	119.50	162.32	171.39	198.47	162.92
**MAE**					
COVIDhub-ensemble^11^	41.12	47.73	54.93	62.83	51.65
ARGOX Ensemble	47.74 (#3)	57.71 (#3)	63.50 (#2)	83.95 (#3)	63.22 (#2)
UMass-MechBayes^42^	44.66	55.95	68.88	85.64	63.78
MOBS-GLEAM COVID^43^	51.60	61.42	71.95	82.28	66.81
LANL-GrowthRate^44^	53.06	65.19	75.92	88.42	70.65
UA-EpiCovDA^45^	54.75	68.63	77.35	84.58	71.33
epiforecasts-ensemble^8^	58.02	71.39	82.30	97.42	77.28
Naive	51.65	68.30	86.54	110.24	77.97
**Correlation**					
COVIDhub-ensemble^11^	0.82	0.79	0.76	0.72	0.77
ARGOX Ensemble	0.79 (#3)	0.76 (#2)	0.73 (#2)	0.68 (#2)	0.74 (#2)
UMass-MechBayes^42^	0.80	0.73	0.67	0.62	0.70
MOBS-GLEAM COVID^43^	0.78	0.73	0.68	0.63	0.70
LANL-GrowthRate^44^	0.78	0.71	0.67	0.62	0.69
UA-EpiCovDA^45^	0.74	0.66	0.61	0.60	0.65
epiforecasts-ensemble^8^	0.74	0.67	0.62	0.54	0.64
Naive	0.75	0.69	0.59	0.49	0.64

The RMSE, MAE, Pearson correlation and their averages are reported. Methods are sorted based on their average. Our ARGOX-Ensemble’s rankings for each error metric are included in parenthesis.

From Tables 4 and 5, we can observe that our ARGOX-Ensemble model produces competitive accuracy for COVID-19 1 to 4 weeks ahead predictions and is among the top 8 models in term of all three error metrics. Figure S9 further displays ARGOX-Ensemble’s accuracy and robustness in terms of the metrics’ mean and standard deviation range over all 51 states. Overall, all the error metric comparisons demonstrate ARGOX-Ensemble’s competitiveness during the comparison time periods.

## Discussion

While our ARGOX-Ensemble approach shows strong results, its accuracy and robustness depend on the reliability of its inputs. One limitation of our method is that the Google search query volumes are sensitive to media coverage, and such instability could propagate into our COVID-19 death predictions. Fortunately, media driven searches die down as pandemic progresses. In addition, our model also mitigates such instability via adaptive training.

We use the summer period to identify the optimal lag and the 23 highly correlated queries. Such idea of optimal lag captures the intuition that people tend to search before clinic visits. It is interesting to observe different indications of epidemiological plausibility and infections from the optimal delays of the queries (Table S3). COVID-19 incremental death trend has the longest delay from COVID-19 cases and tests related queries (more than 4 weeks), and follows by mild (3–4 weeks) and severe symptoms (2–3 weeks) related queries, indicating symptom to death is in moderate horizon and symptom’s severity increases while the optimal lag decreases. On the other hand, there are still some Google search queries affected by media coverage and public fear. Namely, vaccination related queries have the shortest delays, which is a short-term signal as well as general concern, intensified by news media coverage and spikes in cases or death trends, since the vaccination is not yet available in summer 2020. Nonetheless, our model is able to robustly capture the COVID-19 death trend, by determining the optimal lags during the summer period.

Information in Google search data deteriorates as forecast horizons expands, which could potentially impact the robustness and accuracy of our 4 weeks ahead predictions shown in Fig. 6 and Table 4. Nevertheless, the ${L}_{1}$ penalty and the dynamic training can capture the most relevant search terms and time series information for COVID-19 national level death estimation, and our model is still better than GT-only or AR-only models (Table 1) in all forecasting horizons. Meanwhile, ARGOX-Ensemble is able to robustly select accurate 1–4 weeks ahead state-level predictions from the three ARGOX alternative methods, despite that JHU dataset is only a noisy ground-truth. Models to further alleviate the bias in Internet search data and capture long-term COVID-19 trends could be an interesting future direction.

For national level COVID-19 death, the last week’s COVID-19 death and all the Google search terms have significant effects on the 1 to 4 weeks ahead COVID-19 growth, shown in Figure S5 and S6, which reflects a strong temporal auto-correlation and dependence on people search behaviors. Smoothing the penalized linear regression’s coefficients with past three day’s coefficients further leads to a smooth and continuous estimation curve and prevents undesired spikes (Figs. 3, 4, 5, 6). ARGO also allows us to transparently understand how Google search information and historical COVID-19 information complement one another. For instance, past week COVID-19 death contributes positively to the current national COVID-19 death trend predictions, shown in Supplementary Materials (Figure S5), which indicates that the current trend is likely to follow from the past week’s growth/drop. Figure S5 and S5 also indicates time-varying between COVID-19 death trend and people’s search behavior for COVID-19 related terms (general and symptom related searches). In the national COVID-19 1 to 4 weeks ahead predictions, time series models tend to have delay responses to sudden changes and are easily carried away by the changes, as shown in Figs. 3, 4, 5, 6 from December 2020 to March 2021. Google search information, on the other hand, is better at reacting to sudden changes, but is also sensitive to public’s overreaction embedded in the search frequencies. Fortunately, the adaptive training can help ARGO achieves fast self-correction in the subsequent week.

For state level, besides producing ARGO state level predictions using the same framework as national level ARGO, we effectively combine state, regional, and national level publicly available data from Google searches and delayed COVID-19 cases and death to produce ARGOX-2Step state level estimations. ARGOX-NatConstraint improve upon ARGOX-2Step by restricting the sum of state level death predictions to be similar to national level death predictions, as ARGO national level predictions have already shown its strength. Both ARGOX-2Step and ARGOX-NatConstraint incorporate geographical and temporal correlation of COVID-19 death to provide accurate, reliable 1 to 4 weeks ahead estimations. To further improve accuracy and robustness, we combine all three methods and produce winner-takes-all ensemble forecast for 1 to 4 weeks ahead state level deaths. ARGO and ARGOX-2Step are unified frameworks adapted directly from influenza prediction with minimal changes, which demonstrates their robustness and general applicability, while reducing the possibility of over-fitting. Furthermore, the winner-takes-all ensemble approach efficiently combines all three frameworks and is able to outperform the constituent models for all states in all 1 to 4 weeks ahead predictions. Our national model and state-level performances are competitive to other state-of-arts models from CDC. Thus, we have shown that adapting ARGOX framework to COVID-19 can achieve accurate and robust results, and our model could serve as a valuable input for the CDC’s current ensemble forecast.

Finally, there are also many other approaches for COVID-19 prediction besides the statistical approach that we took, such as compartmental models^7^ or simulation modeling^47^. Different approaches have their own merits in performance for different date ranges. For example, as the pandemic progresses, deep learning-based methods are showing their advantages in prediction accuracy^48–50^ and intervention inferences^51^ due to the accumulation of data. Our hierarchical structure, with every single step being a linear model, is a principled and data-driven statistical approach, with clear interpretation of how each step and each feature contribute to the overall forecasts. We can zoom in to see how internet search and COVID-19 time series information contribute to each sub-model predictions (Figure S5 and S6), and how each sub-models participate in the final ensemble forecasts (Figure S7, S8). Thus, our proposed is fundamentally different from other modeling structures with competitive accuracy, providing a different angle that complements the existing COVID-19 prediction literature.

## Concluding remarks

In this paper, we demonstrate that methods for influenza prediction method using online search data^12,16,17^ can be re-purposed for COVID-19 prediction. Specifically, by incorporating Google search information and autoregressive information, we could achieve strong performance on national level deaths predictions, while aggregating Google search information and cross-state-regional-national data could achieve competitive performance on state level death predictions, for 1 to 4 weeks ahead COVID-19 death forecasts, compared with other existing COVID-19 methods submitted to CDC. The combination of COVID-19 cases and deaths with optimally delayed Google search information, as well as the utilization of geographical structure, appears to be key factors in the enhanced accuracy of ARGO in national and state level predictions, demonstrating great additional insights which could assist and complement current CDC forecasts.

## Supplementary Information

Supplementary Information.
