# Supplementary material for: COVID-19 forecasts using Internet search information in the United States
Source: Sci Rep. 2022 Jul 7;12:11539. doi: 10.1038/s41598-022-15478-y (PMC9261899; doi:10.1038/s41598-022-15478-y)
Supplement: Supplementary file 1 — Supplementary Information. [file 41598_2022_15478_MOESM1_ESM.pdf]

Supporting Information for COVID-19 Forecasts Using Internet Search Information in the United States

Simin Ma, Shihao Yang

Correspondence to: shihao.yang@isye.gatech.edu This PDF file includes:

- Supplementary Text
- Supplementary Figs. S3a to S66
- Supplementary Tables S1 to S57

This Supplementary Material is organized as following:

Implementation detail for ARGOX 2-Step

In the first step, we use LASSO to aggregate the search volume information in the corresponding area. In the second step, we take a dichotomous approach for the 51 US states/districts, setting apart six states: AK, HI, DE, KY, VT and ME. We first set apart AK and HI, since they are geographically separated from the contiguous US. Then, we determine the rest by computing multiple correlation in COVID-19 incremental death count of each state to the COVID-19 incremental death counts of entire nation, the COVID-19 incremental death counts of the other regions (excluding the region that the state belongs) and the COVID-19 incremental death counts other states. DE, KY, VT and ME are the 4 states that have the lowest multiple correlations. A relatively low multiple correlation of a state implies that the state's COVID-19 death growth trend is not well aligned with other states', other regions' or the whole nation, indicating that information cross the other states or other regions might not help the stand-alone 6 states' death prediction. Therefore, we incorporate the dichotomous approach from ARGOX [17] on the 45 "joint" states, and 6 "alone" states.

First Step

For the first step, using the same notation as in "ARGO-Inspired Prediction" Section, we extract region/state level internet search information in region/state m for day $T+l$ by estimating $\hat{y}_{T+l,m}$ using Google search terms with equation (4), for $l > 0$.

$$\hat{y}_{T+l,m} = \hat{\mu}_{y,m} + \sum_{k=1}^{K} \hat{\delta}_{k,m} X_{k,T+l-\hat{O}_k,m} + \sum_{r=1}^{6} \hat{\gamma}_{r,m} \mathbb{I}_{\{T+l,r\}} \tag{4}$$

where $X_{i,t,m}$ is the Google Trends data of search term i day t of region/state m and $\mathbb{I}_{\{T+l,r\}}$ is a weekday r indicator for the forecast date $T+l$. The coefficients $\{\mu_{y,m}, \boldsymbol{\delta} = (\delta_{1,m}, \ldots, \delta_{K,m}), \boldsymbol{\gamma} = (\gamma_{1,m}, \ldots, \gamma_{6,m}\}$ are obtained via

$$\underset{\mu_{y,m},\boldsymbol{\delta},\gamma_m,\boldsymbol{\lambda}}{\operatorname{argmin}} \sum_{t=T-M-l+1}^{T-l} \left(y_{t+l,m} - \mu_{y,m} - \sum_{k=1}^{27} \delta_{k,m} X_{k,t+l-\hat{O}_k,m} + \sum_{r=1}^{6} \gamma_{r,m} \mathbb{I}_{\{T+l,r\}} \right)^2 + \lambda_\delta \|\boldsymbol{\delta}\|_1 + \lambda_\gamma \|\boldsymbol{\gamma}\|_1 \tag{5}$$

We set $M = 56$ days for training, and $\boldsymbol{\lambda} = \{\lambda_\delta, \lambda_\gamma\}$ through cross-validation, where we let $\lambda_\delta = \lambda_\gamma$ for simplicity. Additionally, we use $K = 23$ highly correlated Google search terms, and let $\hat{O}_k = \max(O_k, l)$ as the adjusted optimal lag of kth Google search term subject to l^{th} day ahead prediction. Denote the regional estimates obtained as $(\hat{y}^{reg}_{T,1}, \ldots, \hat{y}^{reg}_{T,10})$ and state estimates obtained as $(\hat{y}^{GT}_{T,1}, \ldots, \hat{y}^{GT}_{T,51})$.

Lastly, we obtain national COVID-19 death estimate $\hat{y}^{nat}_T$ using equation (1). Since all the estimates obtained above are daily COVID-19 incremental death, we aggregate them into 1 to 4 weeks total incremental death and work on 1 to 4 weeks ahead death forecast separately using the following steps. We denote index τ for weekly indexing and t for daily indexing.

Second Step

For the 45 joint states, we gather the raw estimates for state/regional/national-level weekly COVID-19 incremental deaths from the first step, as well as lag-1 state-level COVID-19 incremental deaths, and

concatenate them into a VAR-X (vector autoregressive with exogenous variables) format. We then jointly estimate the 45 states' week τ COVID-19 incremental deaths with the VAR-X format via best linear predictor with ridge-regression inspired shrinkage [17].

Specifically, we obtain our raw estimates for the state-level weekly COVID-19 incremental deaths: $\hat{\boldsymbol{y}}_\tau^{GT} = (\hat{y}_{\tau,1}^{GT}, \ldots, \hat{y}_{\tau,45}^{GT})^\intercal$, expanded national and regional level COVID-19 death estimates: $\hat{\boldsymbol{y}}_\tau^{nat} = (\hat{y}_\tau^{nat}, \ldots, \hat{y}_\tau^{nat})^\intercal$ and $\hat{\boldsymbol{y}}_\tau^{reg} = (\hat{y}_{\tau,r_1}^{reg}, \ldots, \hat{y}_{\tau,r_{45}}^{reg})^\intercal$, where r_m is the region number for state m, and the previous week state-level groudtruth: $\boldsymbol{y}_{\tau-1} = (y_{\tau-1,1}, \ldots, y_{\tau-1,45})^\intercal$. We denote the state-level death increment at week τ as $\boldsymbol{Z}_\tau = \Delta \boldsymbol{y}_\tau = \boldsymbol{y}_\tau - \boldsymbol{y}_{\tau-1}$. Then, we concatenate the four estimates above into a VAR-X structure: $\boldsymbol{W}_\tau = [(\boldsymbol{Z}_{\tau-1})^\intercal, (\hat{\boldsymbol{y}}_\tau^{GT} - \boldsymbol{y}_{\tau-1})^\intercal, (\hat{\boldsymbol{y}}_\tau^{reg} - \boldsymbol{y}_{\tau-1})^\intercal, (\hat{\boldsymbol{y}}_\tau^{nat} - \boldsymbol{y}_{\tau-1})^\intercal]$, where the VAR-X structure consist of lag-1 groundtruth and three exogenous variables. Finally, we use a L_2 penalized best linear predictor to estimate the VAR-X structure and obtain the state-level predictions of week τ for 45 joint states all together. We use 15-weeks training window for parameter estimations.

For the 6 alone states, we take a stand-alone modeling approach [17], focusing on estimating the individual state's COVID-19 1 to 4 weeks ahead incremental death by integrating the within-state and national information in the second step. Specifically, we use 3 predictors, previous week state-level groundtruth, state level and national level COVID-19 estimates, $\hat{y}_{\tau,m}^{GT}, \hat{y}_\tau^{nat}$, for $m \in \{\text{AK, HI, DE, KY, VT and ME}\}$, where the regional terms are dropped. In other words, the VAR-X structure for the alone state m is $W_{\tau,m} = [(Z_{\tau-1,m})^\intercal, (\hat{y}_{\tau,m}^{GT} - y_{\tau-1,m})^\intercal, (\hat{y}_{\tau,m}^{nat} - y_{\tau-1,m})^\intercal]$, Similarly, we use the best linear predictor with ridge-regression inspired shrinkage [17] to estimate the VAR-X structure and get the final estimates for each of the 6 alone states, with 15-weeks training period.

2

Detail Derivation for ARGOX-Nat-Constraint

The detailed derivation for constrained optimization problem in equation (3) is shown as follows. For simplicity, we dropped week index τ. We assume that $\boldsymbol{Z}_\tau, \boldsymbol{W}_\tau$ are demeaned in this case. Additionally, we rewrite the constraint in equation (3) as $\mathbf{1}^\intercal \boldsymbol{A}\boldsymbol{W}_\tau = \tilde{y}$ for simplicity, by denoting $\tilde{y} = \hat{y}^{*\text{ARGO}}_{\tau,nat} - \mathbf{1}^\intercal \boldsymbol{y}_{\tau-1} - \mathbf{1}^\intercal \mu_Z$.

We first re-write the original optimization as a Lagrangian function after some simplification as :

$$f(A,\lambda) = \text{Tr}(\Sigma_{ZZ}) + \text{Tr}(\boldsymbol{A}\Sigma_{WW}\boldsymbol{A}^\intercal) - \text{Tr}(2\Sigma_{ZW}A^\intercal) + \lambda(\tilde{y} - \mathbf{1}^\intercal \boldsymbol{A}\boldsymbol{W}) \tag{6}$$

where $\Sigma_{ZZ} = \text{Var}(\boldsymbol{Z})$, $\Sigma_{WW} = \text{Var}(\boldsymbol{W})$ and $\Sigma_{ZW} = \text{Cov}(\boldsymbol{Z},\boldsymbol{W})$ and are all constructed through ARGOX original setup [17]. Then, we can re-write the original problem in equation (3) as:

$$\begin{aligned} &\min_{\boldsymbol{A},\lambda} f(A) \\ &\text{s.t. } \lambda(\tilde{y} - \mathbf{1}^\intercal \boldsymbol{A}\boldsymbol{W}) = 0 \end{aligned} \tag{7}$$

By taking derivative with respect to $\boldsymbol{A}$ and λ, we have

$$\begin{cases} \nabla_{\boldsymbol{A}} f(A,\lambda) &= 2\Sigma_{WW}A^\intercal - 2\Sigma_{ZW}^\intercal - \lambda \boldsymbol{W}\mathbf{1}^\intercal \\ \nabla_\lambda f(A,\lambda) &= \tilde{y} - \mathbf{1}^\intercal \boldsymbol{A}\boldsymbol{W} \end{cases}$$

After setting them to 0 for optimally condition and solve for $\boldsymbol{A}$ and λ, we have:

$$\begin{cases} \boldsymbol{A} &= \left(\Sigma_{ZW} + \frac{\lambda}{2}\mathbf{1}^\intercal \boldsymbol{W}^\intercal\right)\Sigma_{WW}^{-1} \\ \lambda &= \frac{2}{n\boldsymbol{W}^T\Sigma_{WW}^{-1}\boldsymbol{W}}\left(\tilde{y} - \mathbf{1}^\intercal \Sigma ZW \Sigma_{WW}^{-1}\boldsymbol{W}\right) \end{cases} \tag{8}$$

where $n = 49$ is the length of vector $\mathbf{1}$. Let $\widetilde{\boldsymbol{W}}_\tau = \boldsymbol{W}_\tau - \mu_W$ be the demeaned predictor. Our estimate for the increment at week t is

$$\hat{\boldsymbol{Z}}_\tau = \mu_Z + \hat{\boldsymbol{A}}\widetilde{\boldsymbol{W}}_\tau = \mu_Z + \left(\Sigma_{ZW} + \frac{1}{n\widetilde{\boldsymbol{W}}_\tau^T\Sigma_{WW}^{-1}\widetilde{\boldsymbol{W}}_\tau}\left(\tilde{y} - \mathbf{1}^\intercal\Sigma_{ZW}\Sigma_{WW}^{-1}\widetilde{\boldsymbol{W}}_\tau\right)\mathbf{1}^\intercal\widetilde{\boldsymbol{W}}_\tau^\intercal\right)\Sigma_{WW}^{-1}\widetilde{\boldsymbol{W}}_\tau$$

Thus, our final prediction for state level COVID-19 week τ incremental death is

$$\hat{\boldsymbol{y}}_\tau = \hat{\boldsymbol{y}}_{\tau-1} + \mu_Z + \left(\Sigma_{ZW} + \frac{1}{n\widetilde{\boldsymbol{W}}_\tau^T\Sigma_{WW}^{-1}\widetilde{\boldsymbol{W}}_\tau}\left(\tilde{y} - \mathbf{1}^\intercal\Sigma_{ZW}\Sigma_{WW}^{-1}\widetilde{\boldsymbol{W}}_\tau\right)\mathbf{1}^\intercal\widetilde{\boldsymbol{W}}_\tau^\intercal\right)\Sigma_{WW}^{-1}\widetilde{\boldsymbol{W}}_\tau \tag{9}$$

Moreover, we use the ridge-regression inspired shrinkage to modify the estimate, by replacing Σ_{ZW} as $\frac{1}{2}\Sigma_{ZW}$ and Σ_{WW} as $(\frac{1}{2}\Sigma_{WW} + \frac{1}{2}D_{WW})$ where D_{WW} is the diagonal of the empirical covariance of $\boldsymbol{W}_\tau$:

$$\hat{\boldsymbol{Z}}_\tau = \mu_Z + \left(\frac{1}{2}\Sigma_{ZW} + \frac{1}{n\widetilde{\boldsymbol{W}}_\tau^T\left(\frac{1}{2}\Sigma_{WW} + \frac{1}{2}D_{WW}\right)^{-1}\widetilde{\boldsymbol{W}}_\tau}\left(\tilde{y} - \frac{1}{2}\mathbf{1}^\intercal\Sigma_{ZW}\left(\frac{1}{2}\Sigma_{WW} + \frac{1}{2}D_{WW}\right)^{-1}\widetilde{\boldsymbol{W}}_\tau\right)\mathbf{1}^\intercal\widetilde{\boldsymbol{W}}_\tau^\intercal\right) \left(\frac{1}{2}\Sigma_{WW} + \frac{1}{2}D_{WW}\right)^{-1}\widetilde{\boldsymbol{W}}_\tau$$

Therefore, our final prediction for state-level COVID-19 week t incremental death with ridge inspired shrinkage is:

$$\hat{\boldsymbol{y}}_\tau = \hat{\boldsymbol{y}}_{\tau-1} + \mu_Z + \left(\Sigma_{ZW} + \frac{1}{n\widetilde{\boldsymbol{W}}_\tau^T\left(\Sigma_{WW} + D_{WW}\right)^{-1}\widetilde{\boldsymbol{W}}_\tau}\left(\tilde{y} - \mathbf{1}^\intercal\Sigma_{ZW}\left(\Sigma_{WW} + D_{WW}\right)^{-1}\widetilde{\boldsymbol{W}}_\tau\right)\mathbf{1}^\intercal\widetilde{\boldsymbol{W}}_\tau^\intercal\right) \left(\Sigma_{WW} + D_{WW}\right)^{-1}\widetilde{\boldsymbol{W}}_\tau \tag{10}$$

Selected important Google search queries

Table S1 lists the selected 23 important terms used in this study. They are selected through optimal lag selections.

Table S1: selected 23 important terms

coronavirus vaccine	cough	covid 19 vaccine	coronavirus exposure
coronavirus cases	coronavirus test	covid 19 cases	covid 19
exposed to coronavirus	fever	headache	how long covid 19
how long contagious	loss of smell	loss of taste	nausea
pneumonia	rapid covid 19	rapid coronavirus	robitussin
sore throat	sinus	symptoms of the covid 19	

4

Google search queries Optimal lagged Pearson correlations

Table S2 shows the optimal lag delayed Google search queries' Pearson correlation with COVID-19 daily incremental death.

Table S2: Optimal Lagged Google Query and COVID-19 Death Pearson Correlation from 2020-04-01 to 2020-06-30

Google Query	Pearson Correlation	Google Query	Pearson Correlation
loss of taste	0.909	covid 19 how long	0.223
loss of smell	0.877	normal body	0.223
how long contagious	0.864	body temperature	0.222
covid 19 vaccine	0.815	cold vs coronavirus	0.205
rapid covid 19	0.782	coronavirus vs cold	0.205
pneumonia	0.761	expectorant	0.203
robitussin	0.738	acute bronchitis	0.186
bronchitis	0.724	covid 19 hospital	0.178
sinus	0.711	high fever	0.157
cough	0.699	covid 19 relief	0.153
covid 19	0.649	human temperature	0.153
fever	0.649	is coronavirus contagious	0.147
symptoms of the covid 19	0.64	normal body temperature	0.14
how long covid 19	0.636	signs of the coronavirus	0.14
sore throat	0.636	contagious coronavirus	0.129
coronavirus test	0.628	coronavirus contagious	0.129
coronavirus cases	0.622	shortness of breath	0.125
strep throat	0.605	coronavirus vitamin c	0.089
coronavirus exposure	0.573	oseltamivir	0.082
coronavirus vaccine.	0.549	coronavirus test kit	0.079
exposed to coronavirus	0.523	covid 19 treatment	0.073
rapid coronavirus	0.513	cold and coronavirus	0.061
upper respiratory	0.51	coronavirus and cold	0.061
headache	0.507	how long does the coronavirus last	0.054
the covid 19	0.506	how long does coronavirus last	0.052
covid 19 cases	0.506	symptoms of covid 19	0.049
nausea	0.502	coronavirus medication	0.048
tessalon	0.491	coronavirus family	0.039
symptoms of pneumonia	0.482	taking temperature	0.037
oscillococcinum	0.476	do i have the coronavirus	0.036
strep	0.473	respiratory coronavirus	0.035
nasal congestion.	0.468	covid 19 what to do	0.034
common cold	0.446	coronavirus hospital	0.03
chest cold	0.434	i have the coronavirus	0.018
walking pneumonia	0.397	coronavirus care	0.013
coronavirus relief	0.357	covid 19 symptoms	0.011
covid 19 test	0.336	ear thermometer	0.01
cough fever	0.331	coronavirus how long	0.01
fever cough	0.331	how long coronavirus	0.01
covid 19 care	0.24	coronavirus recovery	0.005
sinus infections	0.23	how to treat coronavirus	0.004
reduce fever	0.223	coronavirus cough	0.001

5

Google searches optimal lag

Table S3 shows the selected 23 Google search queries' optimal lag (delay), selected through fitting regression of lagged terms against COVID-19 death trend and select the lag with minimal mean-squared error.

Table S3: selected 23 important terms' optimal lag ranked by lags

Google Search Term	Optimal Lag
coronavirus vaccine	5
covid 19 vaccine	7
coronavirus.exposure	13
robitussin	15
sinus	15
covid 19	21
how long covid 19	21
symptoms of the covid 19	21
nausea	23
cough	24
fever	24
exposed to coronavirus	24
loss of taste	24
loss of smell	25
how long contagious	25
rapid coronavirus	27
pneumonia	28
rapid covid 19	28
headache	29
sore throat	30
coronavirus cases	30
coronavirus test	30
covid 19 cases	30

6

ARGO-Inspired Model Sensitivity to Weekday Seasonality Effect

We use a weekday indicator in our ARGO-Inspired method since there is weekday seasonality in COVID reporting in the US. This section investigates our methods' sensitivity towards the weekday seasonality effect. In particular, we modified our ARGO-Inspired method by using 7-day moving average smoothed reported cases, death and Google search terms (with corresponding optimal lag) as features in the L_1 penalized linear model (Method section equation 1), and forecast future 1-4 weeks national-level COVID-19 deaths. We denote the method “ARGO-Smooth”, and compare this method against “ARGO”, and the naive method. For fair comparison, both “ARGO-Smooth” and “ARGO” uses 56 days training period, and same optimal-lags for important Google search queries.

For future 1-4 weeks forecasts, “ARGO-Smooth” will produce a single weekly point prediction for each 1-4 weeks (since the features are 7-day smoothed), whereas “ARGO” will produce 7 daily point prediction for each 1-4 weeks and aggregate as weekly predictions. “ARGO” also uses past 3-day smoothed coefficients for each daily point prediction (see Method Section). In summary, “ARGO-Smooth” smoothing is done on input-level, whereas “ARGO” smoothing is done on output-level.

Table S4 below displays the national level 1-4 weeks ahead COVID-19 death forecast performance. ARGO-Smooth is similar compared to ARGO for 1 week ahead forecasts but fall short for 2-4 weeks. Figure S1 further displays the forecasting trends of “ARGO-Smooth”, “ARGO” and Naive methods. “ARGO-Smooth” forecasts exhibits more noise and fluctuations when there are sudden change in COVID-19 death trends compared to “ARGO”, especially during winter 2021 (re-openings) and winter 2022 (Omicron variant). One possible reason is “ARGO” produces daily forecasts and then aggregates into weekly forecasts, which is able to pick-up those sudden fluctuations better than “ARGO-Smooth” which only produces weekly forecasts.

Table S4: National Level Comparison Error Metrics

	1 Week Ahead	2 Weeks Ahead	3 Weeks Ahead	4 Weeks Ahead
RMSE				
Naive	2042.664	2901.271	3773.094	4577.691
ARGO-Smooth	1800.287	2901.515	3683.510	4152.673
ARGO	**1779.708**	**2182.819**	**2905.771**	**3863.739**
MAE				
Naive	1433.897	2144.364	2945.750	3601.648
ARGO-Smooth	1203.713	1907.807	2416.568	2720.170
ARGO	**1167.598**	**1553.648**	**1997.670**	**2609.636**
Correlation				
Naive	0.933	0.863	0.768	0.656
ARGO-Smooth	0.949	0.883	0.801	0.810
ARGO	**0.950**	**0.925**	**0.892**	**0.814**

National level 1 to 4 weeks ahead COVID-19 incremental prediction comparisons in 3 error metrics. Boldface highlights the best performance for each metric in each study period. All comparisons are based on the original scale of COVID-19 national incremental death.

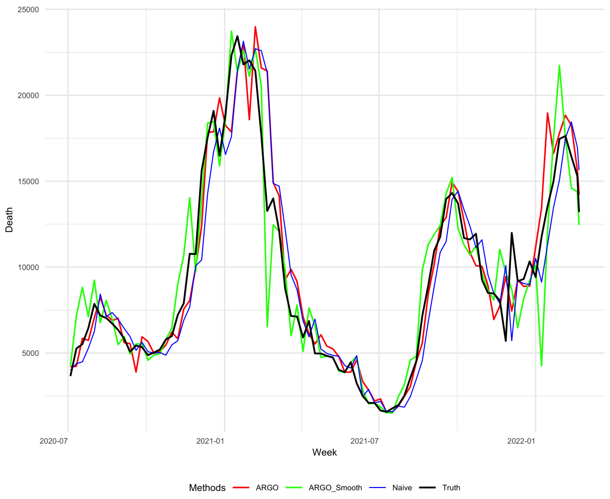

(a) 1 Week Ahead National Level Predictions

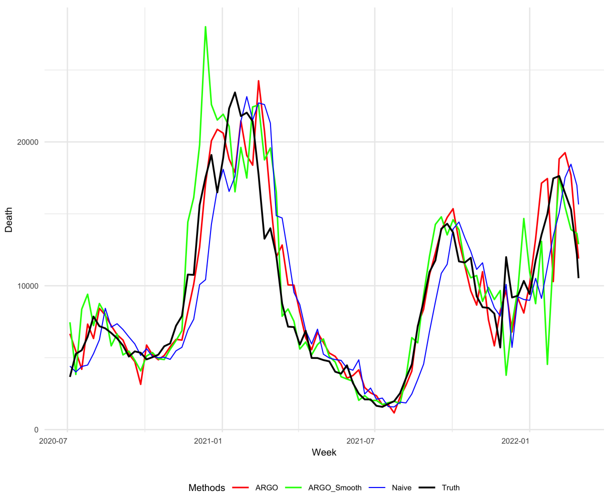

(b) 2 Weeks Ahead National Level Predictions

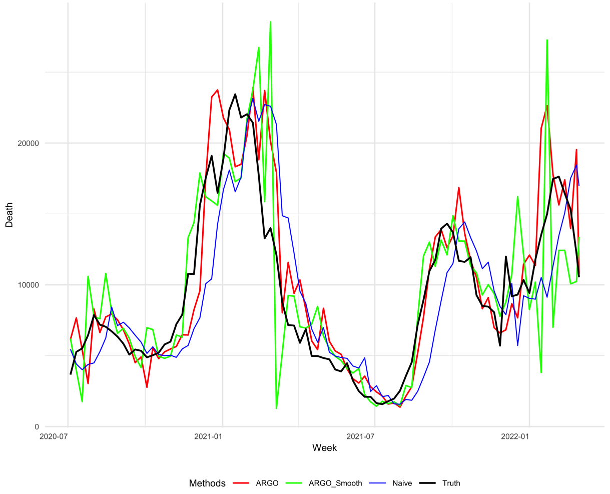

(c) 3 Weeks Ahead National Level Predictions

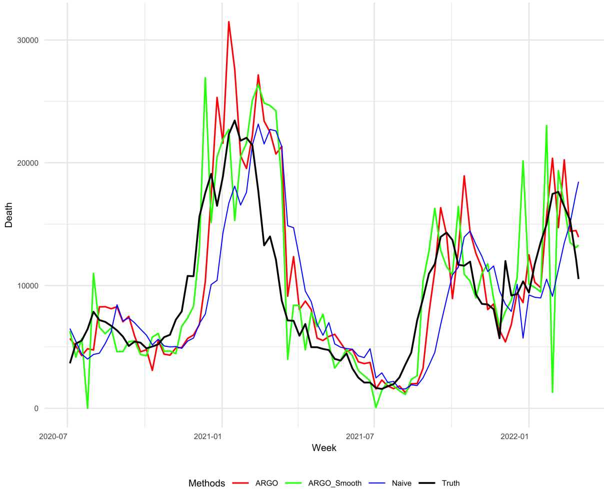

(d) 4 Weeks Ahead National Level Predictions

Figure S1: 1 to 4 weeks ahead national level COVID-19 weekly incremental death predictions' comparisons weekly from 2020-07-04 to 2022-03-05. The method included are ARGO, ARGO-Smooth, Naive (persistence), truth. Estimation results for COVID-19 1 (top left), 2 (top right), 3 (bottom left), and 4 (bottom right) weeks ahead weekly incremental death. ARGO estimations (red), contrasting with the true COVID-19 death from JHU dataset (black) as well as the estimates from ARGO-Smooth (Green), and Naive (blue).

8

ARGO-Inspired Model Sensitivity to Hospitalization Predictor

Following the logic of every death case must be a sever case before he/she dies, severe cases might contain potential predictive power towards COVID-19 deaths. In the United States, the severe cases can be acquired through new hospital admissions released by U.S. Department of Health and Human Services (HHS) [46]. In the week of 2020-11-06, HHS confirmed hospital admissions as the ground truth for hospitalizations, whereas there was no official source for hospitalization data identified nor collected prior to that week. On 2020-12-01, CDC COVID-19 Forecast Hub [11] officially incorporated the hospitalization data published by HHS. Meanwhile, hospitalization data was not available in some states, such as Hawaii and Washington D.C., until January 2021. On the other hand, we collected COVID-19 cases and deaths at the start of the COVID-19 pandemic (from 2020-01-21 to 2022-03-05) and use the summer 2020 period to determine our models' hyperparameters (such as important Google queries selection, optimal lags, etc.). We conduct COVID-19 deaths forecasts' retrospective evaluation starting 2020-07-04, to illustrate our model's robustness and accuracy in different COVID-19 periods (variants) by efficiently harnessing the predictive power in the search queries. Therefore, we do not consider using hospitalization as one of the COVID-19 death predictors due to data availability. However, we will investigate hospitalization's predictive power towards COVID-19 deaths in this section.

Specifically, we will evaluate four models for national COVID-19 deaths predictions: (1) persistence (Naive), (2) GT+AR+Hosp prediction, (3) GT+AR prediction, (4) AR+Hosp prediction. The Naive (persistence) predictions use the current week's death counts from NYT as next 1-4 weeks estimation. GT+AR+Hosp prediction uses the model setup in "ARGO Inspired Method" with additional delayed national-level hospitalization time series information. GT+AR prediction is the exact same ARGO model displayed in the main text that uses Google search and COVID-19 time series information (COVID-19 cases and death). AR+Hosp prediction uses delayed COVID-19 time series, and hospitalization information. For fair comparisons, the delayed national-level hospitalization time series considers 2 and 3 weeks lag, and all three ARGO models above use 56 days training period and include weekday indicators. Daily national COVID-19 deaths are estimated using four methods above, and aggregated into weekly deaths for the time period of January 2, 2021 to March 5th 2022, due to Hospitalization data availability.

Table S5 below displays the national level 1-4 weeks ahead COVID-19 death forecast performance. AR+Hosp is similar compared to GT+AR and GT+AR+Hosp for 1 week ahead forecasts but fall short for 2-4 weeks. Figure S2 further displays the forecasting trends of GT+AR+Hosp, GT+AR, AR+Hosp. The forecasting trends using hospitalization is quite robust comparing to using GT, but exhibit more noise and fluctuations when there are sudden surge in hospitalization or deaths, for example during the recent outbreak of Omicron variant (January 2022 to March 2022). This is because an outbreak leading surges in hospitalization will immediately impact the forecasting trend under the rolling-window prediction fashion. Also, AR+Hosp estimates suffer from more delaying behavior than the other two estimates in 4 weeks ahead forecasts, since hospitalization and COVID-19 cases/deaths signals lose their strength under the longer forecasting horizon, whereas GT+AR and GT+AR+Hosp can rely on those Google search terms with long optimal lags from COVID-19 deaths (see Table S3). In summary, hospitalization itself (with COVID-19 autoregressive information) is not powerful enough to predict COVID-19 deaths. On the other hand, hospitalization can serve as a strong additional short-term feature to Google search queries and COVID-19 cases/deaths information, and further assist and guide ARGO (GT+AR) predictions. However, due to data availability, we will not consider hospitalization in this study.

9

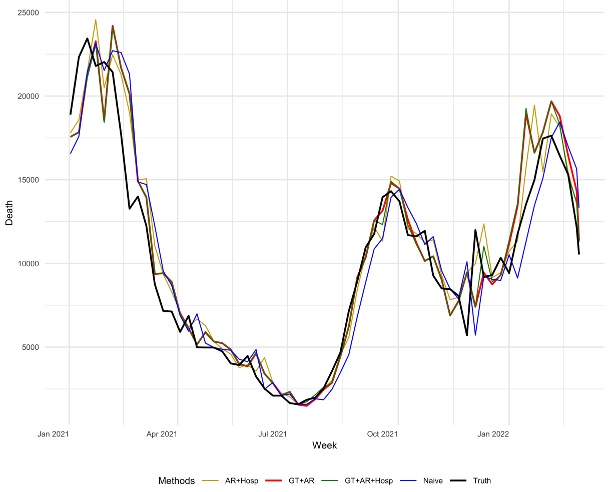

(a) 1 Week Ahead National Level Predictions

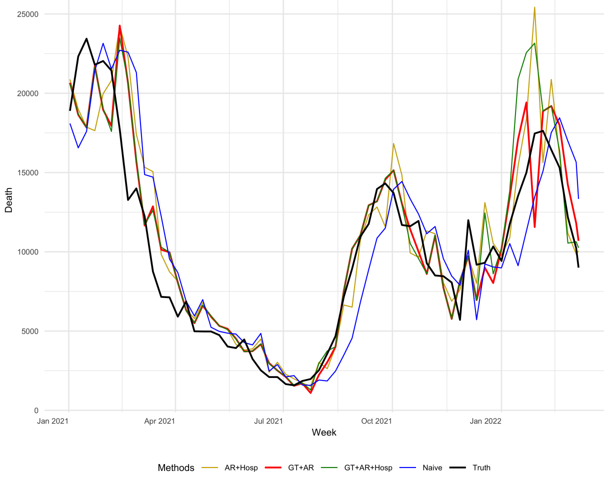

(b) 2 Weeks Ahead National Level Predictions

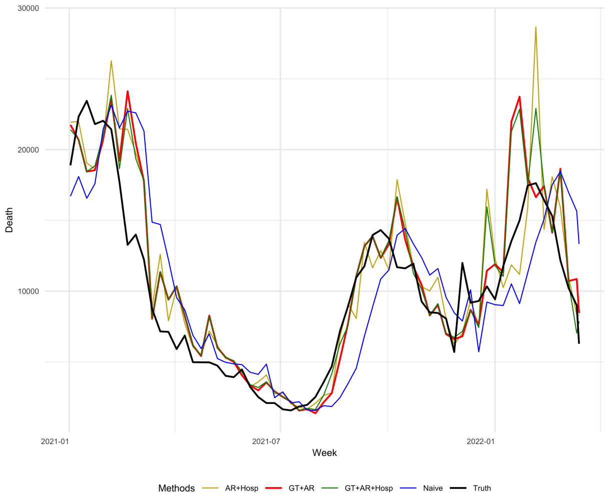

(c) 3 Weeks Ahead National Level Predictions

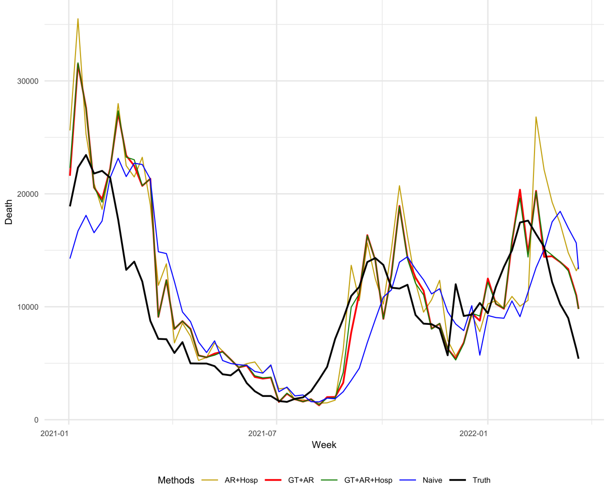

(d) 4 Weeks Ahead National Level Predictions

Figure S2: 1 to 4 weeks ahead national level COVID-19 weekly incremental death predictions' comparisons weekly from 2021-01-02 to 2022-03-05. The method included are AR+Hosp, GT+AR, GT+AR+Hosp, Naive (persistence), truth. Estimation results for COVID-19 1 (top left), 2 (top right), 3 (bottom left), and 4 (bottom right) weeks ahead weekly incremental death. GT+AR estimations (red), contrasting with the true COVID-19 death from JHU dataset (black) as well as the estimates from GT+AR+Hosp (Green), AR+Hosp (Gold), and Naive (blue).

10

Table S5: National Level Comparison Error Metrics

	1 Week Ahead	2 Weeks Ahead	3 Weeks Ahead	4 Weeks Ahead
RMSE				
Naive	2223.661	3090.029	4067.556	5033.468
AR+Hosp	1935.456	2726.080	3121.601	5020.274
GT+AR	1947.385	**2399.870**	3064.312	4035.767
GT+AR+Hosp	**1920.274**	2621.925	**3008.180**	**4010.453**
MAE				
Naive	1585.113	2335.667	3288.453	4100.215
AR+Hosp	1360.758	1971.460	2245.828	3863.923
GT+AR	1373.468	**1778.206**	**2085.062**	2968.646
GT+AR+Hosp	**1356.952**	1879.413	2089.156	**2903.477**
Correlation				
Naive	0.931	0.867	0.769	0.643
AR+Hosp	0.949	0.905	0.888	0.802
GT+AR	0.951	**0.920**	0.893	0.860
GT+AR+Hosp	**0.960**	0.914	**0.900**	**0.863**

National level 1 to 4 weeks ahead COVID-19 incremental prediction comparisons in 3 error metrics. Boldface highlights the best performance for each metric in each study period. All comparisons are based on the original scale of COVID-19 national incremental death for the period of 2021-01-02 to 2022-03-05.

ARGOX-Nat-Constrained motivation

FigS3a, S3b, S4a, and S4b show the inconsistency of aggregated state level ARGOX predictions comparing against ARGO national level predictions for future 1-4 weeks ahead predictions, motivating the development of ARGOX-NatConstraint method.

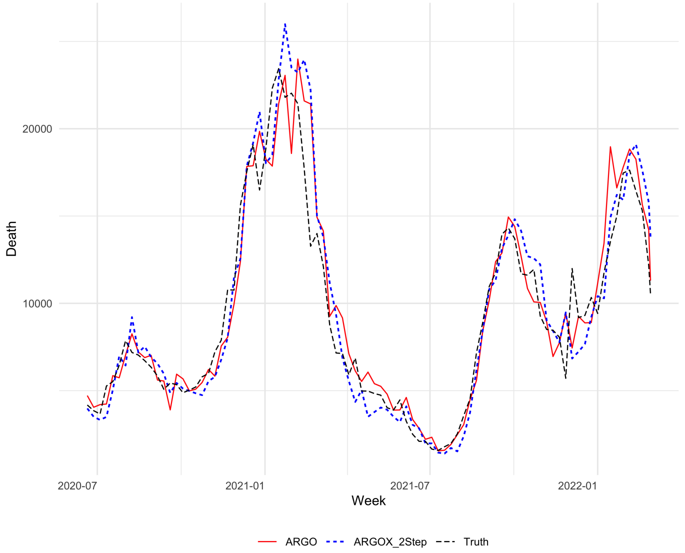

(a) 1 Week Ahead ARGO National Prediction and Summed ARGOX-2Step Predictions

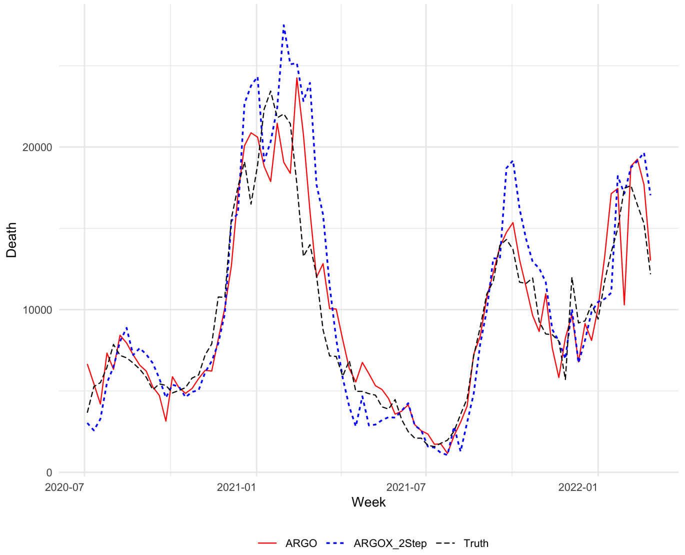

(b) 2 Weeks Ahead ARGO National Prediction and Summed ARGOX-2Step Predictions

Figure S3: The sum of ARGOX-2Step predictions is compared against ARGO national level prediction and JHU COVID-19 true deaths for 1 week ahead (up) and 2 weeks ahead (down) estimations. Illustrates the shortcoming of ARGOX-2Step prediction which doesn't sum up to reasonable national level predictions for both 1 and 2 weeks head prediction.

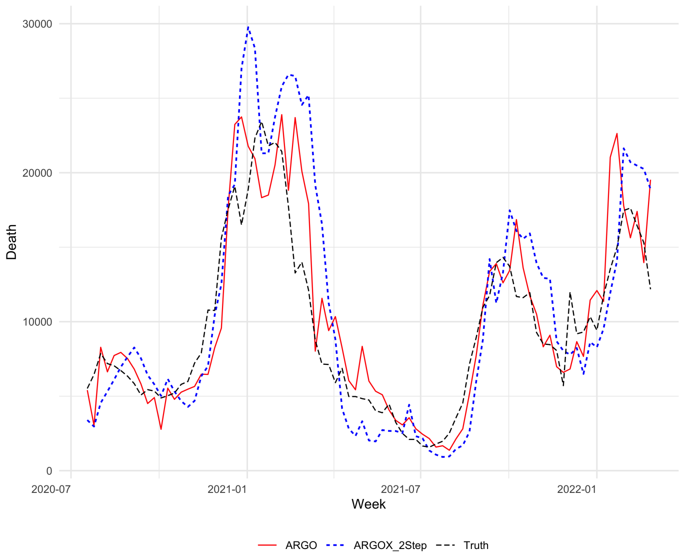

(a) 3 Weeks Ahead ARGO National Prediction and Summed ARGOX-2Step Predictions

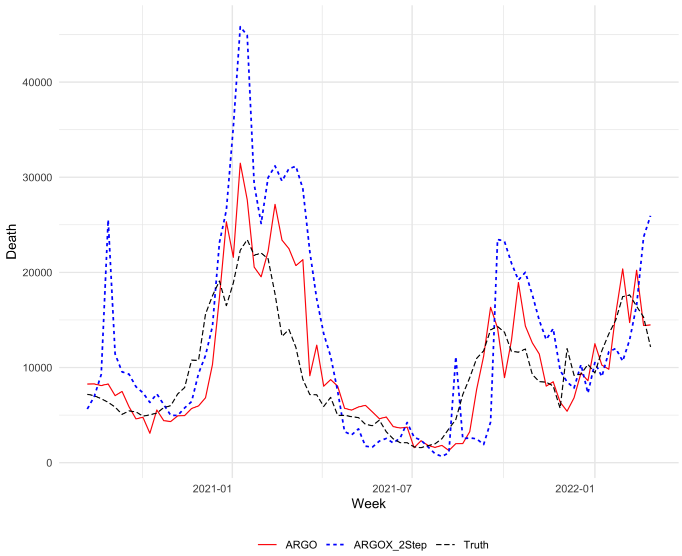

(b) 4 Weeks Ahead ARGO National Prediction and Summed ARGOX-2Step Predictions

Figure S4: The sum of ARGOX-2Step predictions is compared against ARGO national level prediction and JHU COVID-19 true deaths for 3 weeks ahead (up) and 4 weeks ahead (down) estimations. Illustrates the shortcoming of ARGOX-2Step prediction which doesn't sum up to reasonable national level predictions for both 3 and 4 weeks head prediction.

ARGO model parameter heatmaps

Fig S5a, S5b, S6a and S6b show ARGO national level 1 to 4 weeks ahead forecasts' model parameter in heatmaps.

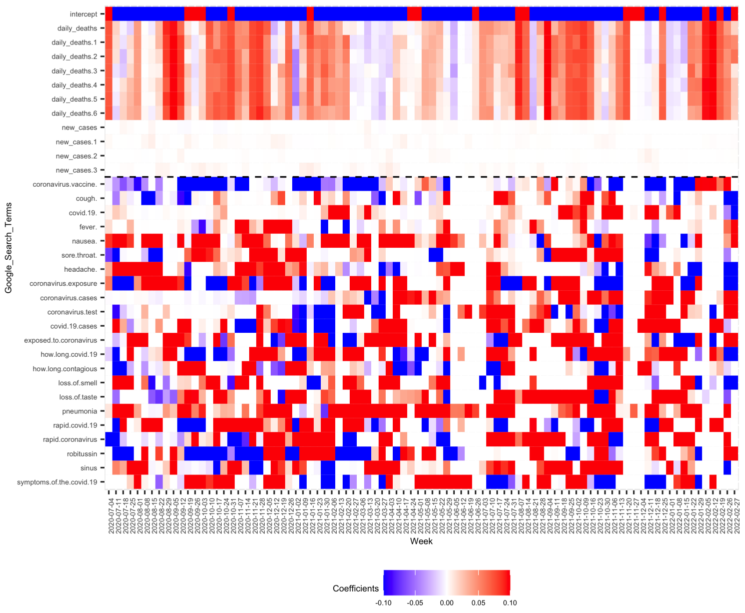

(a) 1 Week Ahead National Level ARGO Coefficients

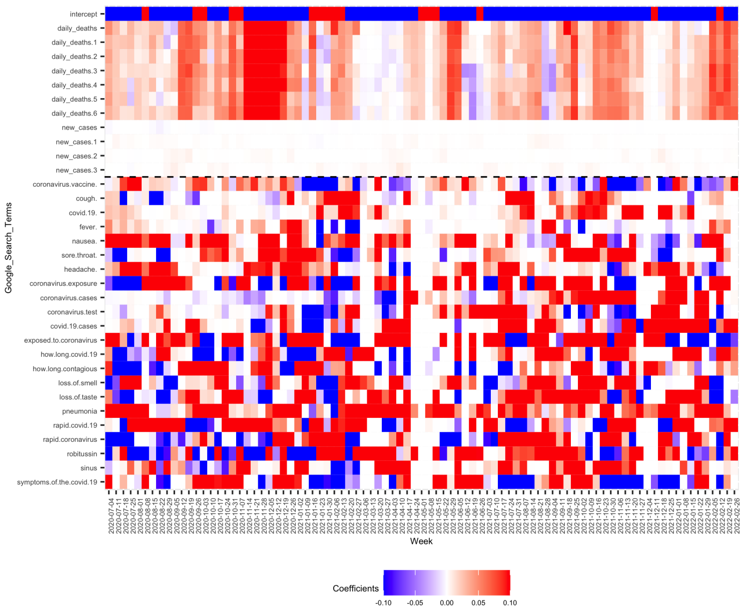

(b) 2 Weeks Ahead National Level ARGO Coefficients

Figure S5: Smoothed coefficients for ARGO national level 1 week and 2 weeks ahead predictions. Coefficients larger than 0.1 are scaled to 0.1 and lower than -0.1 are scaled to -0.1, for simplicity. Red color represents positive coefficients, blue color represents negative coefficients and white color represents zero. Black horizontal dashed line separates Google query queries from autoregressive (cases and death) lags.

ARGO model parameter heatmaps (Continue)

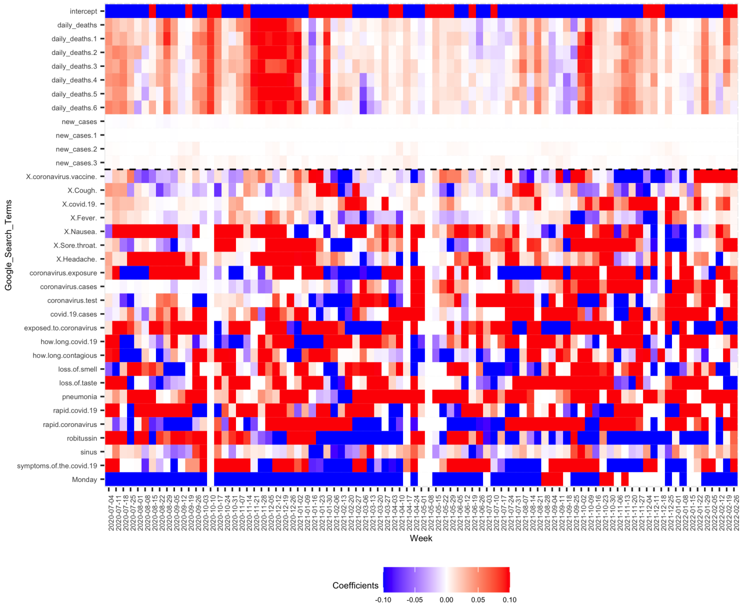

(a) 3 Weeks Ahead National Level ARGO Coefficients

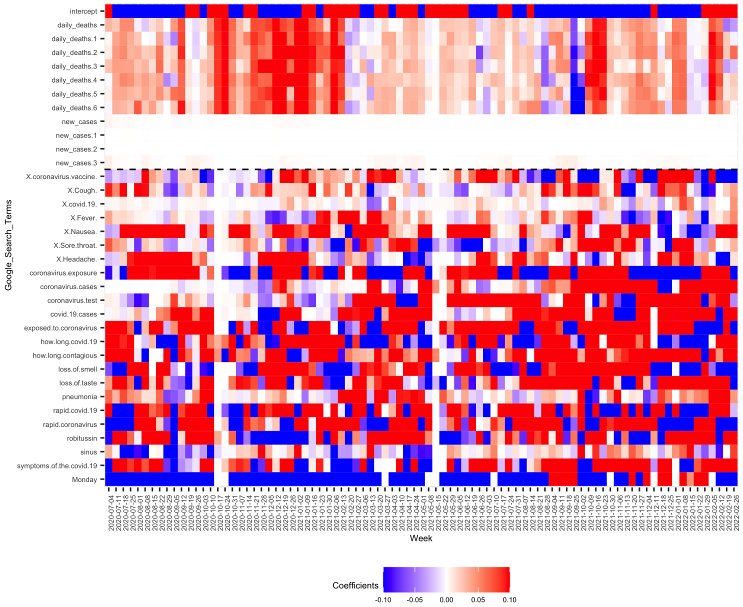

(b) 4 Weeks Ahead National Level ARGO Coefficients

Figure S6: Smoothed coefficients for ARGO national level 3 weeks and 4 weeks ahead predictions. Coefficients larger than 0.1 are scaled to 0.1 and lower than -0.1 are scaled to -0.1, for simplicity. Red color represents positive coefficients, blue color represents negative coefficients and white color represents zero. Black horizontal dashed line separates Google query queries from autoregressive (cases and death) lags.

15

Winner-takes-all ensemble method selections

Fig S7a and S7b show the 1 week and 2 weeks ahead winner-takes-all state level forecasts selection among the three other methods: ARGO, ARGOX-2Step and ARGOX-NatCOnstraint.

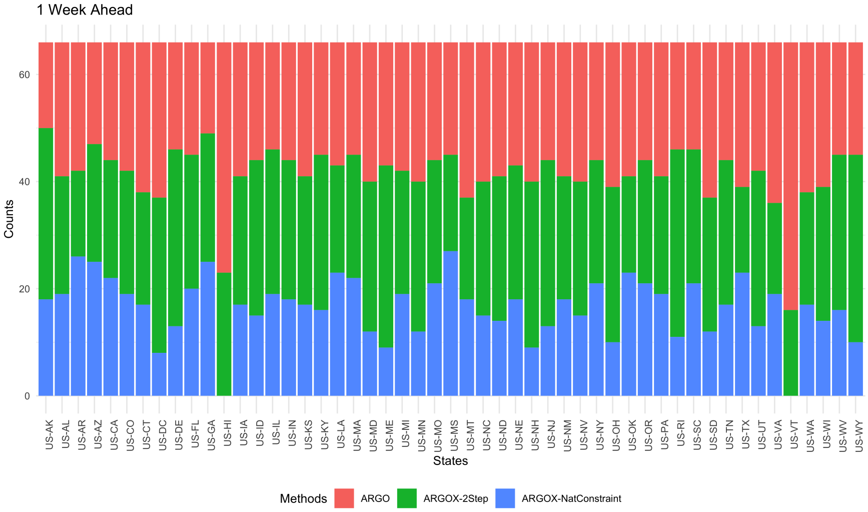

(a) 1 Week Ahead State Level Ensemble Selections

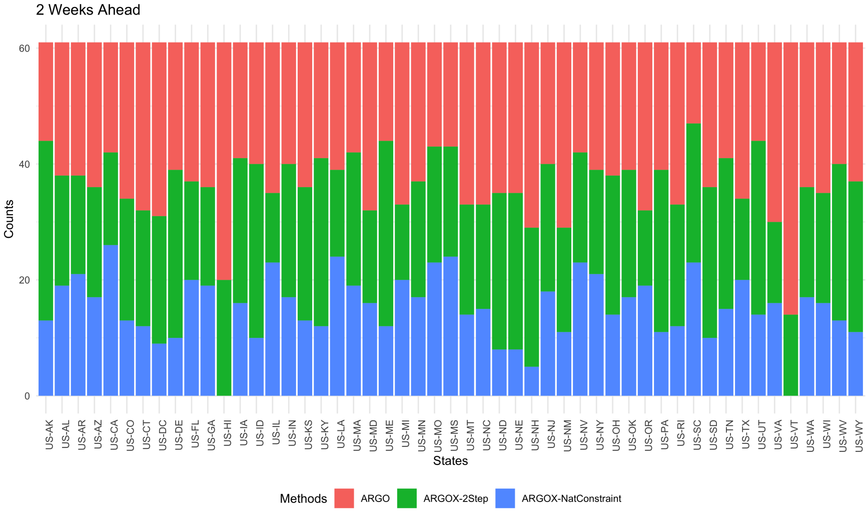

(b) 2 Weeks Ahead State Level Ensemble Selections

Figure S7: Ensemble method's selection among ARGO, ARGOX-2Step and ARGOX-NatConstraint for all 51 U.S. States for 1 week and 2 weeks ahead predictions, from 2020-07-04 to 2022-03-05.

Fig S8a and S8b show the 3 weeks and 4 weeks ahead winner-takes-all state level forecasts selection among the three other methods: ARGO, ARGOX-2Step and ARGOX-NatCOnstraint.

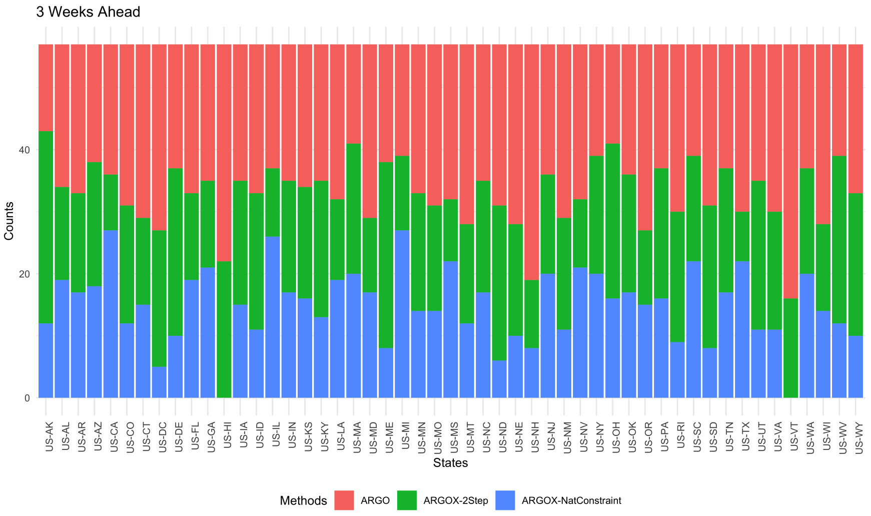

(a) 3 Weeks Ahead State Level Ensemble Selections

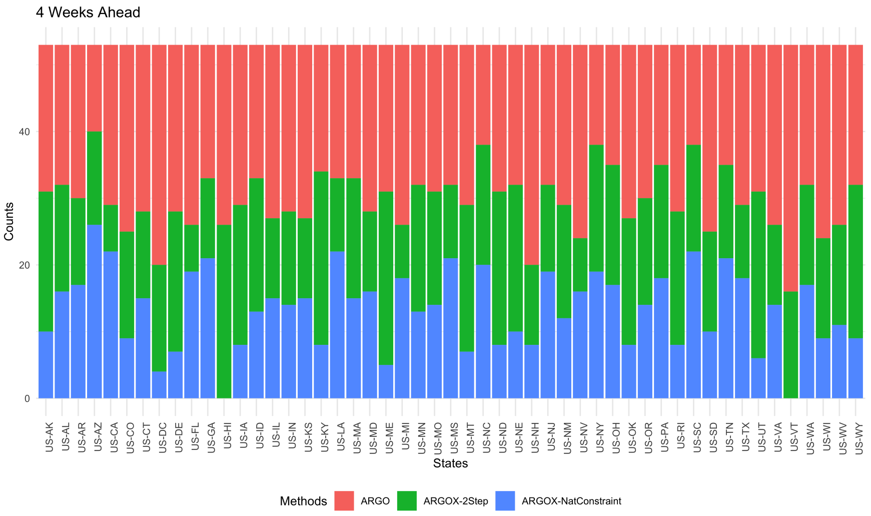

(b) 4 Weeks Ahead State Level Ensemble Selections

Figure S8: Ensemble method's selection among ARGO, ARGOX-2Step and ARGOX-NatConstraint for all 51 U.S. States for 3 weeks and 4 weeks ahead predictions, from 2020-07-04 to 2022-03-05.

State Level Forecasts Coverages

Table S6 shows ARGOX-Ensemble 1 to 4 weeks ahead forecasts' actual coverage for all states. The coverage is for 95% nominal confidence interval. The averages coverage over all states are 0.88%, 0.86% , 0.83%, and 0.78% for 1-4 weeks ahead predictions.

States	1 week ahead	2 weeks ahead	4 weeks ahead	4 weeks ahead
US-AK	0.82	0.77	0.76	0.81
US-AL	0.85	0.79	0.78	0.79
US-AR	0.89	0.82	0.82	0.76
US-AZ	0.87	0.83	0.82	0.75
US-CA	0.88	0.88	0.82	0.75
US-CO	0.88	0.87	0.86	0.80
US-CT	0.84	0.81	0.80	0.74
US-DC	0.90	0.90	0.87	0.89
US-DE	0.91	0.91	0.90	0.85
US-FL	0.82	0.85	0.78	0.76
US-GA	0.94	0.92	0.90	0.81
US-HI	0.89	0.88	0.86	0.86
US-IA	0.86	0.84	0.80	0.74
US-ID	0.88	0.87	0.85	0.79
US-IL	0.89	0.88	0.83	0.75
US-IN	0.89	0.84	0.76	0.72
US-KS	0.86	0.81	0.81	0.72
US-KY	0.88	0.87	0.89	0.91
US-LA	0.88	0.85	0.79	0.81
US-MA	0.88	0.90	0.83	0.80
US-MD	0.91	0.89	0.84	0.84
US-ME	0.88	0.85	0.81	0.78
US-MI	0.93	0.92	0.82	0.85
US-MN	0.89	0.87	0.82	0.78
US-MO	0.91	0.89	0.86	0.80
US-MS	0.85	0.84	0.80	0.72
US-MT	0.83	0.87	0.81	0.82
US-NC	0.91	0.88	0.87	0.70
US-ND	0.84	0.83	0.81	0.75
US-NE	0.87	0.88	0.86	0.84
US-NH	0.89	0.88	0.83	0.79
US-NJ	0.92	0.91	0.88	0.82
US-NM	0.90	0.92	0.88	0.86
US-NV	0.86	0.88	0.85	0.75
US-NY	0.89	0.85	0.84	0.72
US-OH	0.91	0.87	0.82	0.77
US-OK	0.89	0.81	0.85	0.81
US-OR	0.90	0.86	0.85	0.79
US-PA	0.90	0.87	0.78	0.72
US-RI	0.91	0.91	0.85	0.81
US-SC	0.87	0.80	0.79	0.76
US-SD	0.89	0.86	0.86	0.78
US-TN	0.82	0.86	0.80	0.71
US-TX	0.90	0.86	0.82	0.72
US-UT	0.87	0.90	0.88	0.86
US-VA	0.89	0.85	0.86	0.79
US-VT	0.91	0.88	0.85	0.81
US-WA	0.90	0.88	0.83	0.81
US-WI	0.90	0.88	0.86	0.79
US-WV	0.83	0.82	0.76	0.74
US-WY	0.79	0.76	0.76	0.73

Table S6: All States' 1-4 weeks ahead forecast coverage.

More state-level model comparisons

Table 5 shows 1 to 4 weeks ahead state level forecast result comparison the 8 different methods through 3 error metrics. Fig S10 to fig S15 shows the teams comparison in three error metrics as heatmaps throughout 1 to 4 weeks ahead forecasts.

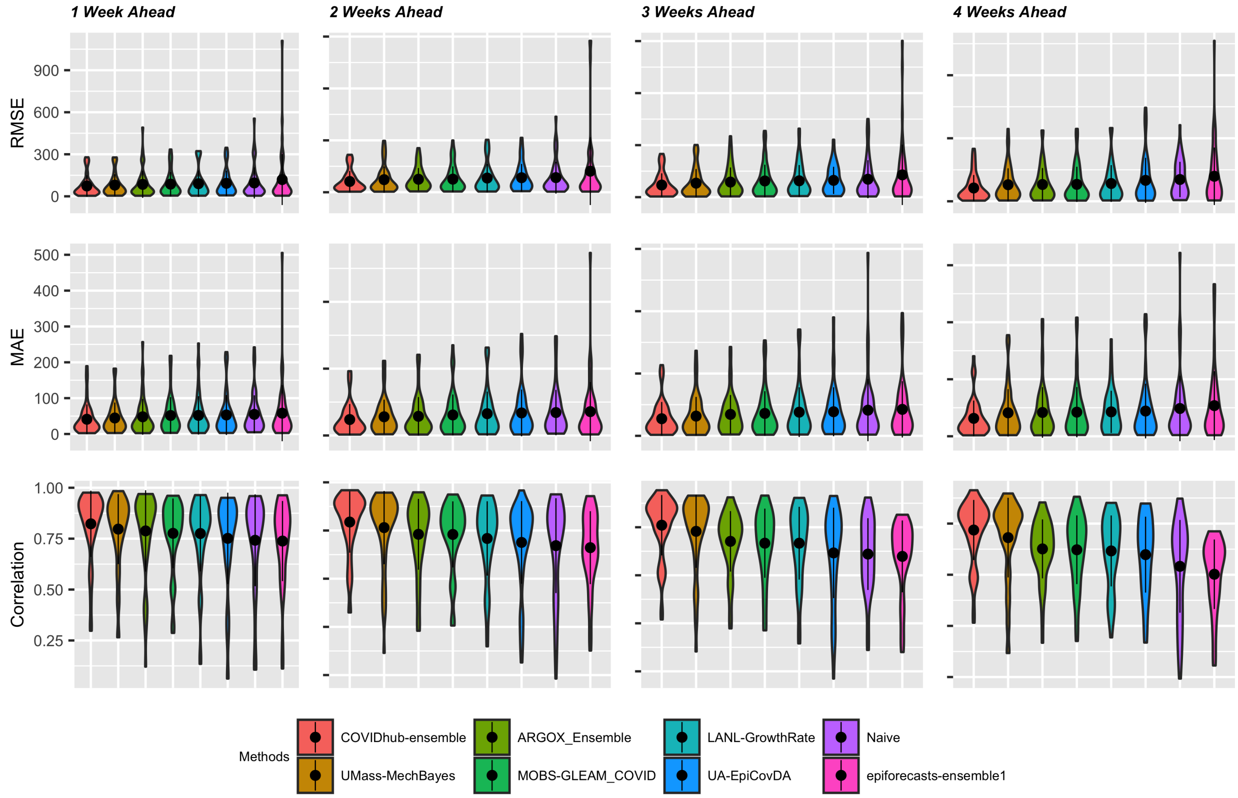

Figure S9: Comparison among different models' 1 to 4 weeks (from left to right) ahead U.S. states level weekly incremental death predictions (from 2020-07-04 to 2022-03-05). The RMSE, MAE and Pearson correlation for each method across all states are reported in the violin plot. The methods (x-axis) are sorted based on their RMSE.

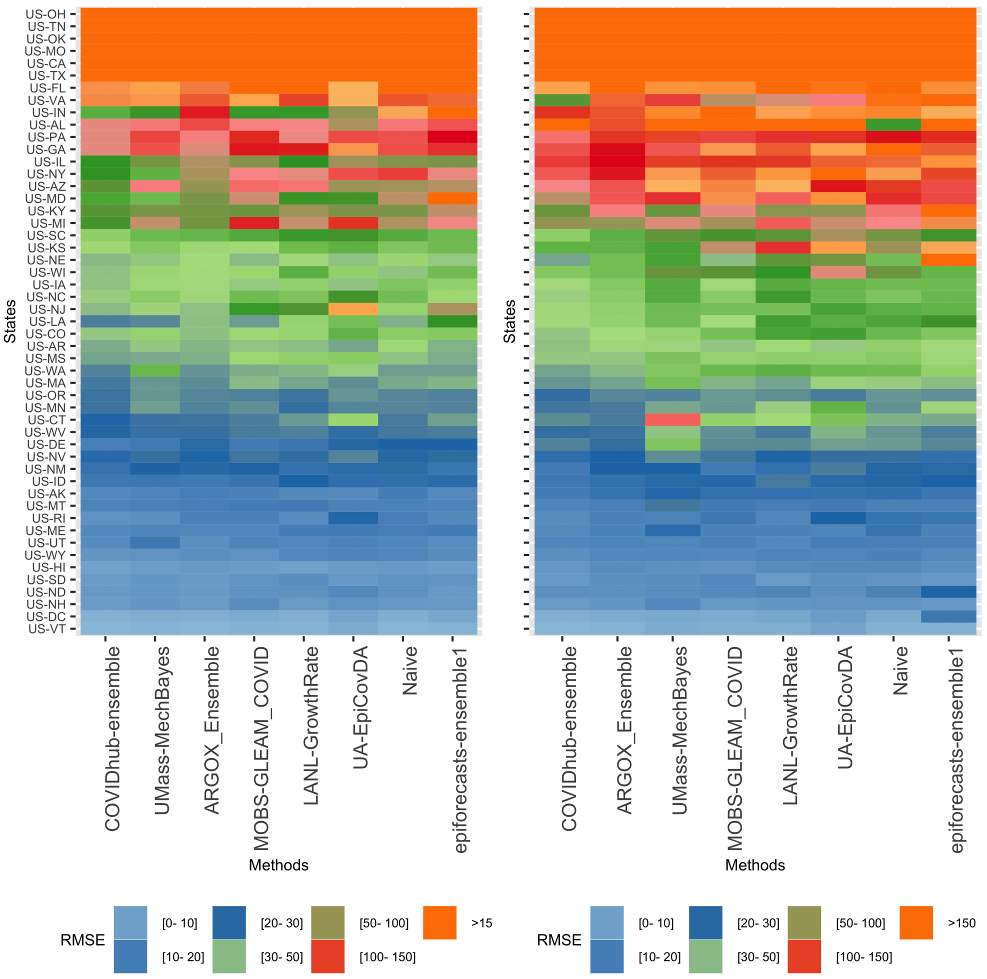

Figure S10: State Level 1 week (left) and 2 weeks (right) ahead all teams RMSE comparison heatmap. States (y-axis) are sorted based on ARGOX-Ensemble's RMSE. RMSE greater than 200 are scaled to 200.

20

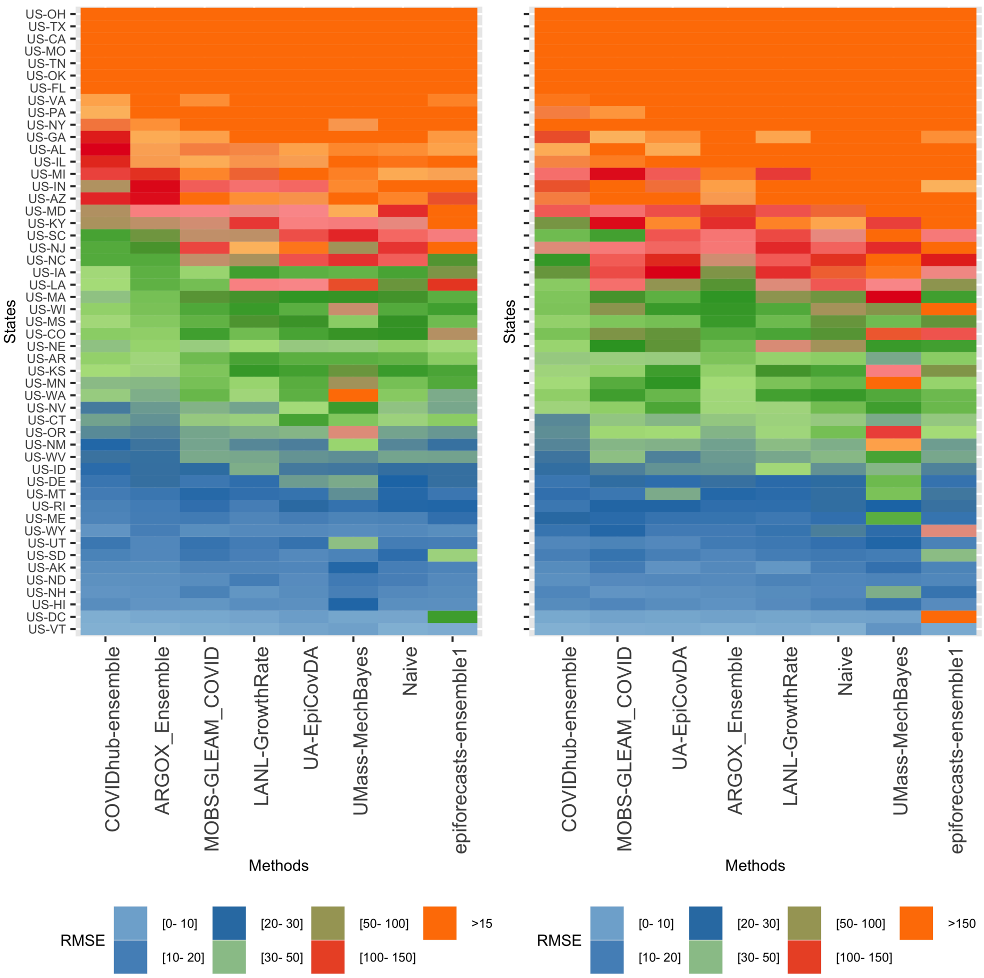

Figure S11: State Level 3 weeks (left) and 4 weeks (right) ahead all teams RMSE comparison heatmap. States (y-axis) are sorted based on ARGOX-Ensemble's RMSE. RMSE greater than 200 are scaled to 200.

21

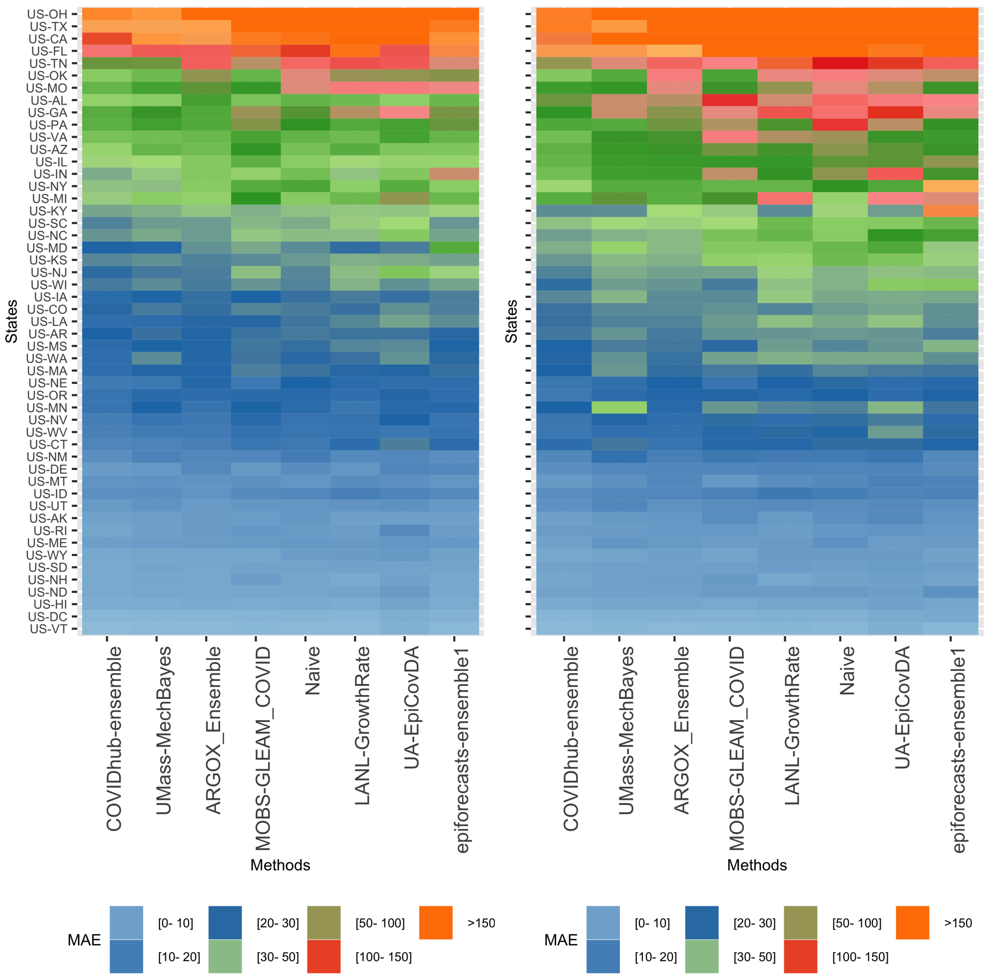

Figure S12: State Level 1 week (left) and 2 weeks (right) ahead all teams MAE comparison heatmap. States (y-axis) are sorted based on ARGOX-Ensemble's MAE. MAE greater than 200 are scaled to 200.

22

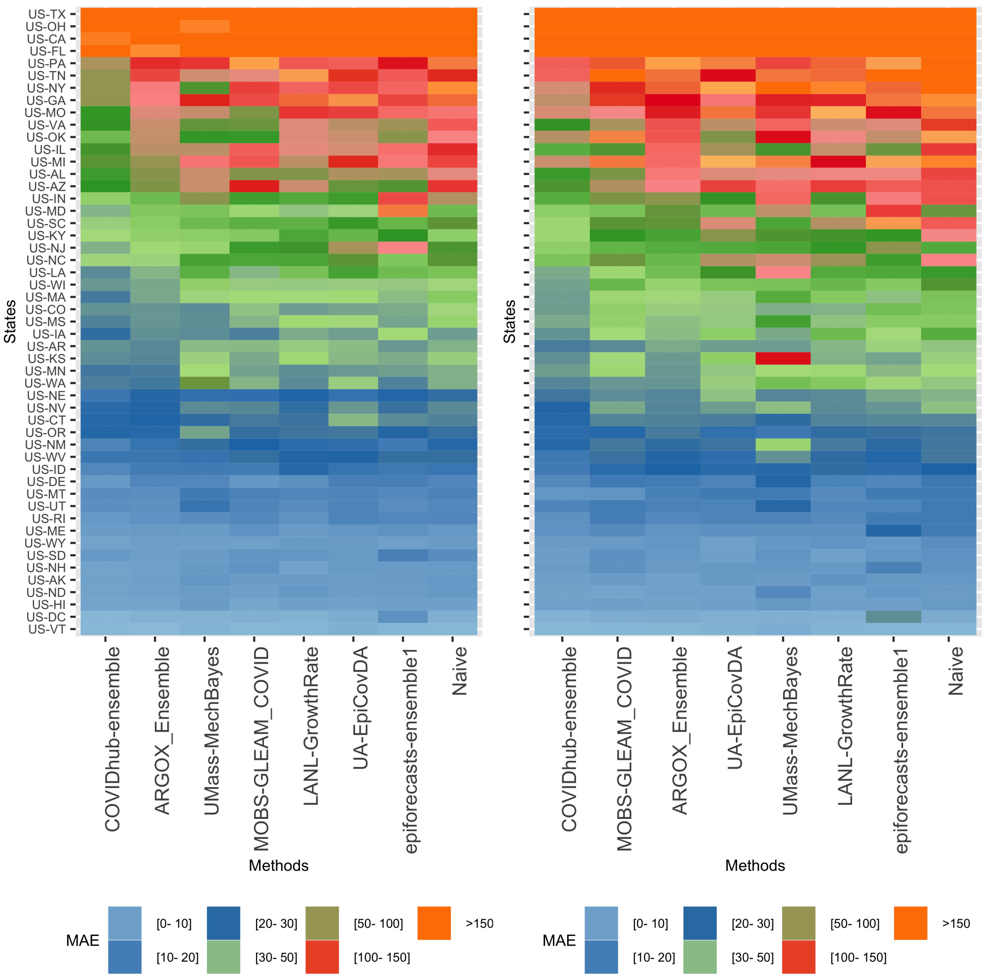

Figure S13: State Level 3 weeks (left) and 4 weeks (right) ahead all teams MAE comparison heatmap. States (y-axis) are sorted based on the best performing method's MAE, in this case ARGOX-Ensemble. MAE greater than 200 are scaled to 200.

23

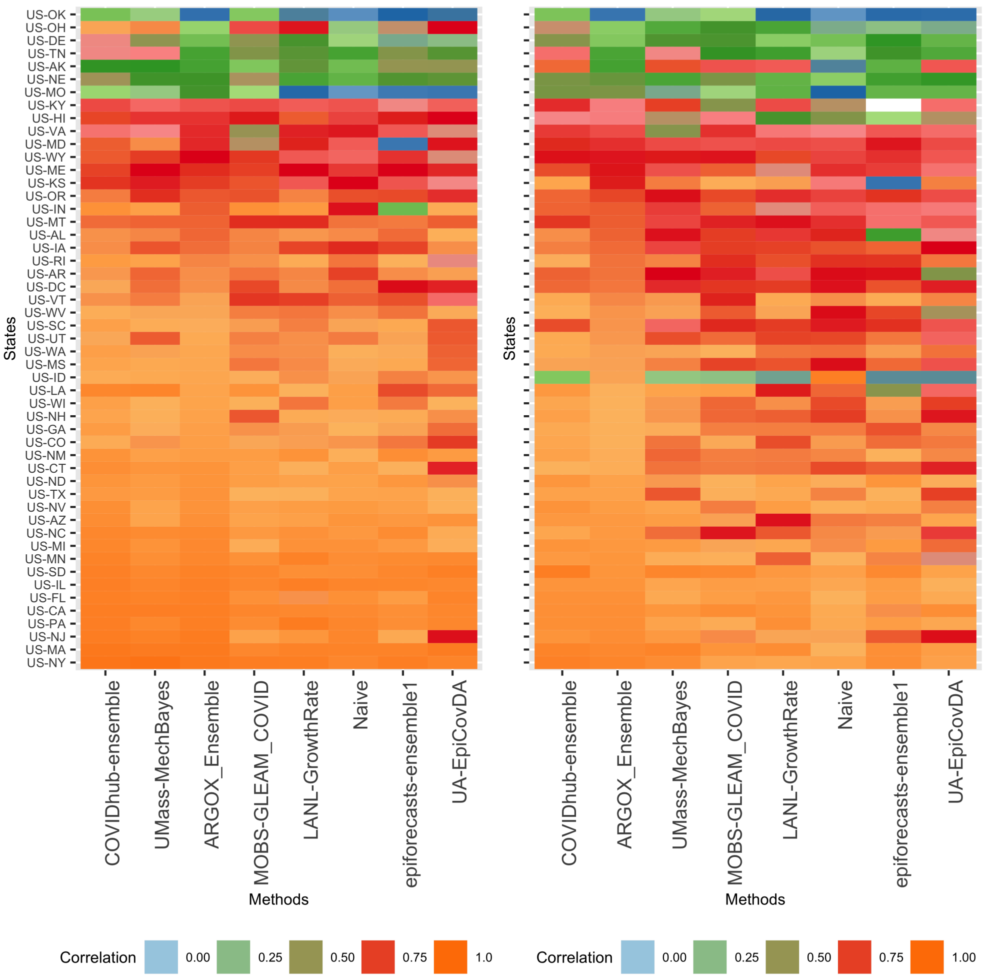

Figure S14: State Level 1 week (left) and 2 weeks (right) ahead all teams Pearson correlation against JHU groundtruth comparison heatmap. States (y-axis) are sorted based on ARGOX-Ensemble's correlation.

24

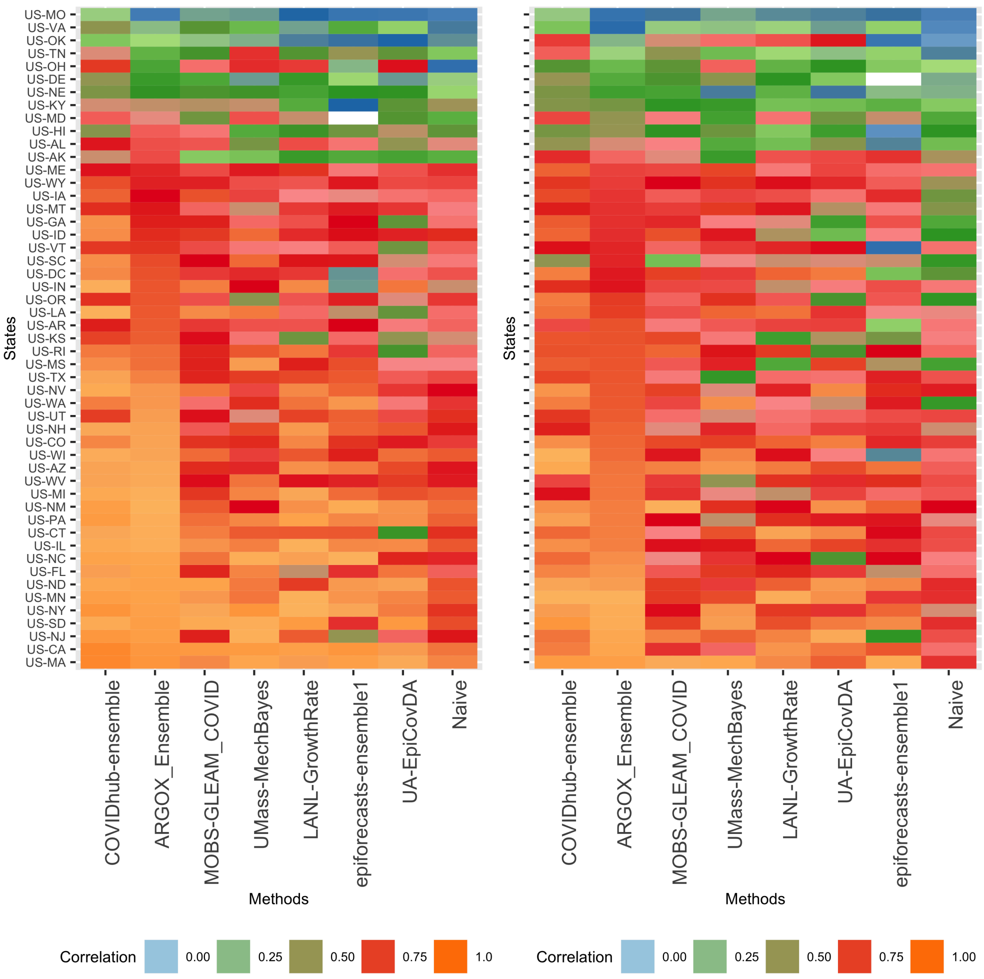

Figure S15: State Level 3 weeks (left) and 4 weeks (right) ahead all teams Pearson correlation against JHU groundtruth comparison heatmap. States (y-axis) are sorted based on ARGOX-Ensemble's correlation.

25

Detailed estimation results for each state

	1 Week Ahead	2 Weeks Ahead	3 Weeks Ahead	4 Weeks Ahead
RMSE				
ARGO	20.90	21.75	19.16	20.43
ARGOX 2Step	19.65	18.28	16.80	19.82
ARGOX NatConstraint	29.45	37.61	58.66	67.92
Ensemble	19.45	18.36	15.18	14.00
Naive	19.92	22.90	19.88	19.57
MAE				
ARGO	12.62	13.43	12.44	12.62
ARGOX 2Step	10.94	10.91	10.17	11.04
ARGOX NatConstraint	19.48	26.03	37.89	41.16
Ensemble	11.19	11.46	9.78	9.40
Naive	12.14	13.45	12.22	12.04
Correlation				
ARGO	0.33	0.25	0.43	0.46
ARGOX 2Step	0.37	0.54	0.65	0.61
ARGOX NatConstraint	0.07	0.15	0.02	0.01
Ensemble	0.40	0.48	0.64	0.68
Naive	0.35	0.15	0.36	0.40

Table S7: Comparison of different methods for state-level COVID-19 1 to 4 weeks ahead incremental death in Alaska (AK). The RMSE, MAE, and correlation are reported.

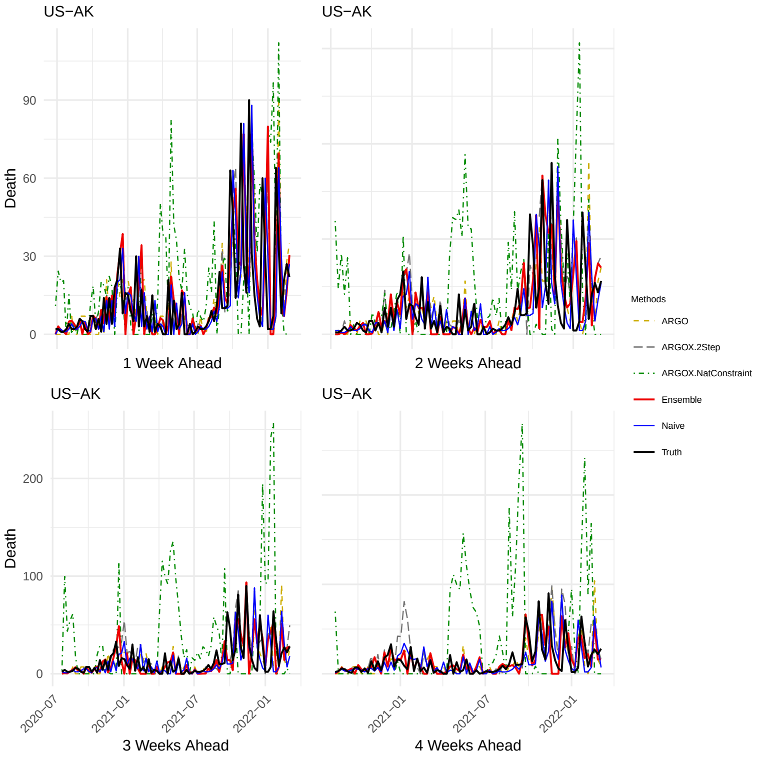

Figure S16: Plots of the COVID-19 1 week (top left), 2 weeks (top right), 3 weeks (bottom left), and 4 weeks (bottom right) ahead estimates for Alaska (AK).

26

	1 Week Ahead	2 Weeks Ahead	3 Weeks Ahead	4 Weeks Ahead
RMSE				
ARGO	141.54	167.53	198.87	267.23
ARGOX 2Step	132.21	176.93	232.77	339.06
ARGOX NatConstraint	126.03	160.35	203.13	276.04
Ensemble	128.86	143.54	169.54	239.79
Naive	117.17	158.03	187.66	241.90
MAE				
ARGO	86.91	98.82	123.22	162.40
ARGOX 2Step	84.05	104.93	135.20	199.41
ARGOX NatConstraint	77.17	96.24	133.17	176.13
Ensemble	79.62	83.37	98.94	131.99
Naive	71.84	93.11	113.35	159.00
Correlation				
ARGO	0.75	0.65	0.49	0.30
ARGOX 2Step	0.81	0.74	0.55	0.39
ARGOX NatConstraint	0.79	0.68	0.46	0.24
Ensemble	0.79	0.78	0.64	0.49
Naive	0.82	0.68	0.55	0.38

Table S8: Comparison of different methods for state-level COVID-19 1 to 4 weeks ahead incremental death in Alabama (AL). The MSE, MAE, and correlation are reported.

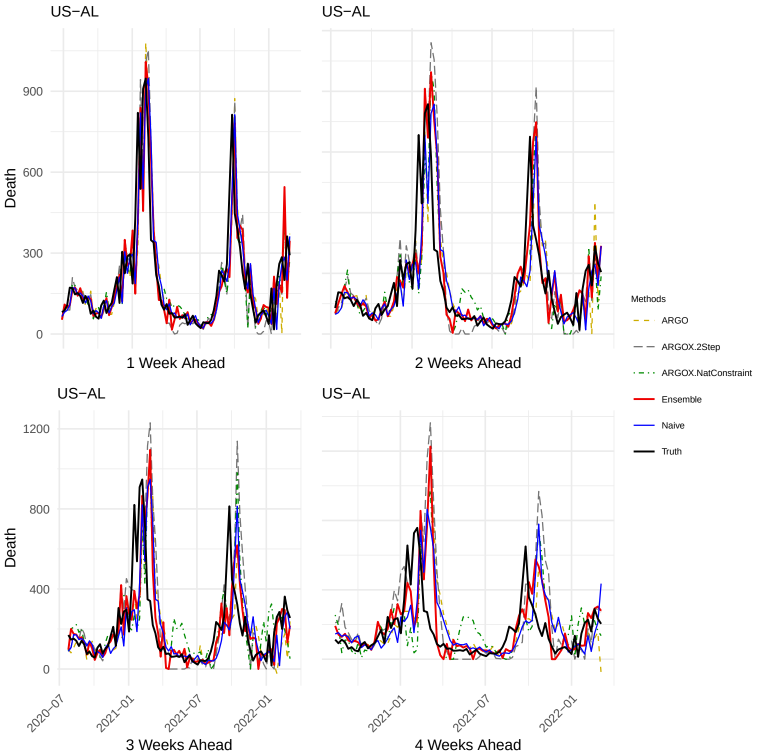

Figure S17: Plots of the COVID-19 1 week (top left), 2 weeks (top right), 3 weeks (bottom left), and 4 weeks (bottom right) ahead estimates for Alabama (AL).

27

	1 Week Ahead	2 Weeks Ahead	3 Weeks Ahead	4 Weeks Ahead
RMSE				
ARGO	54.83	63.56	71.35	99.48
ARGOX 2Step	57.99	75.35	87.64	128.70
ARGOX NatConstraint	60.91	69.38	105.80	112.58
Ensemble	48.74	52.65	56.61	58.13
Naive	58.14	63.45	73.27	85.54
MAE				
ARGO	34.88	44.60	51.47	70.65
ARGOX 2Step	35.92	53.14	62.97	91.87
ARGOX NatConstraint	41.07	51.60	77.27	87.31
Ensemble	29.09	35.16	39.77	44.42
Naive	34.62	42.60	49.36	59.99
Correlation				
ARGO	0.77	0.69	0.61	0.43
ARGOX 2Step	0.78	0.72	0.66	0.53
ARGOX NatConstraint	0.70	0.62	0.32	0.27
Ensemble	0.83	0.80	0.77	0.77
Naive	0.75	0.71	0.61	0.51

Table S9: Comparison of different methods for state-level COVID-19 1 to 4 weeks ahead incremental death in Arkansas (AR). The MSE, MAE, and correlation are reported.

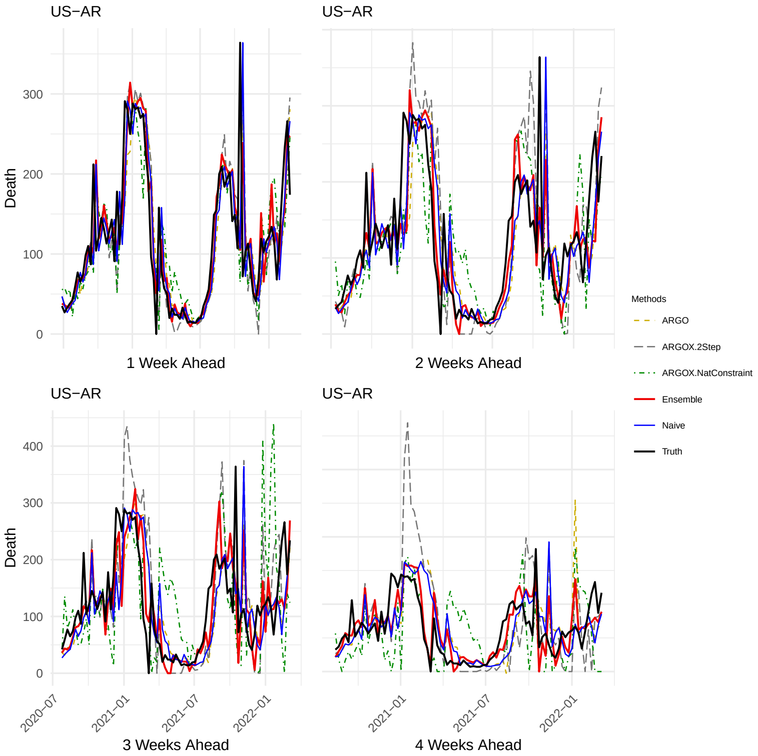

Figure S18: Plots of the COVID-19 1 week (top left), 2 weeks (top right), 3 weeks (bottom left), and 4 weeks (bottom right) ahead estimates for Arkansas (AR).

28

	1 Week Ahead	2 Weeks Ahead	3 Weeks Ahead	4 Weeks Ahead
RMSE				
ARGO	132.15	156.76	178.61	245.17
ARGOX 2Step	118.72	174.46	225.18	348.41
ARGOX NatConstraint	107.11	146.23	174.84	211.47
Ensemble	99.81	132.32	143.17	173.66
Naive	101.50	157.11	195.68	245.72
MAE				
ARGO	89.53	110.06	122.78	162.16
ARGOX 2Step	81.38	122.68	148.34	217.70
ARGOX NatConstraint	72.28	99.82	125.76	162.14
Ensemble	69.07	91.70	96.57	121.44
Naive	67.51	105.62	137.01	172.16
Correlation				
ARGO	0.86	0.80	0.75	0.67
ARGOX 2Step	0.91	0.83	0.78	0.78
ARGOX NatConstraint	0.91	0.82	0.76	0.80
Ensemble	0.92	0.86	0.85	0.92
Naive	0.91	0.80	0.69	0.60

Table S10: Comparison of different methods for state-level COVID-19 1 to 4 weeks ahead incremental death in Arizona (AZ). The MSE, MAE, and correlation are reported.

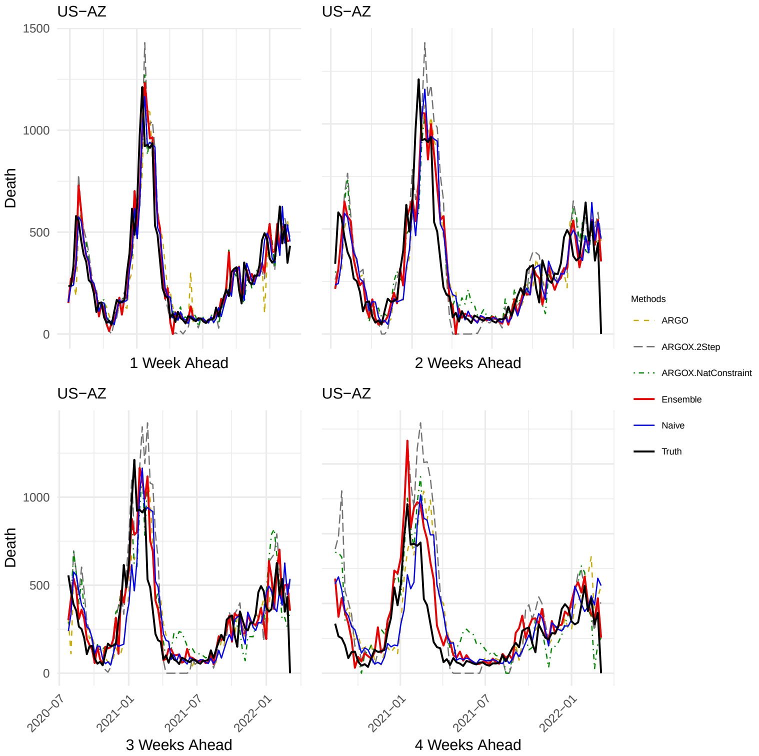

Figure S19: Plots of the COVID-19 1 week (top left), 2 weeks (top right), 3 weeks (bottom left), and 4 weeks (bottom right) ahead estimates for Arizona (AZ).

29

	1 Week Ahead	2 Weeks Ahead	3 Weeks Ahead	4 Weeks Ahead
RMSE				
ARGO	332.12	485.14	517.43	703.58
ARGOX 2Step	305.03	466.04	632.01	1066.13
ARGOX NatConstraint	290.65	399.59	478.81	705.62
Ensemble	254.73	365.26	368.32	554.94
Naive	299.64	393.26	540.17	761.76
MAE				
ARGO	211.80	298.46	332.29	484.55
ARGOX 2Step	191.05	281.59	354.89	697.89
ARGOX NatConstraint	186.54	249.01	302.21	536.13
Ensemble	165.71	217.64	211.15	385.64
Naive	194.50	249.84	353.06	522.06
Correlation				
ARGO	0.92	0.83	0.81	0.69
ARGOX 2Step	0.95	0.90	0.85	0.82
ARGOX NatConstraint	0.94	0.89	0.85	0.84
Ensemble	0.96	0.92	0.91	0.90
Naive	0.94	0.89	0.80	0.68

Table S11: Comparison of different methods for state-level COVID-19 1 to 4 weeks ahead incremental death in California (CA). The MSE, MAE, and correlation are reported.

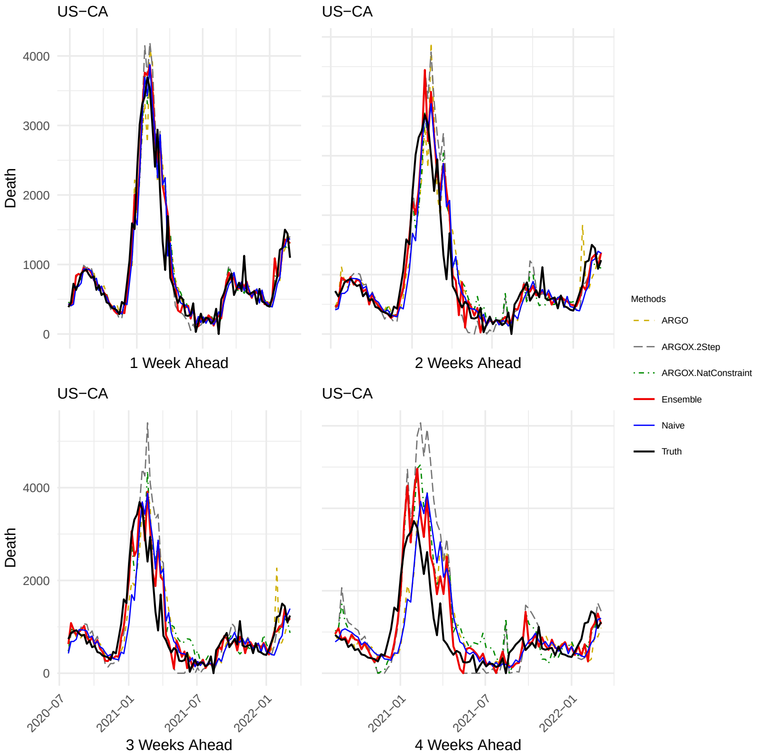

Figure S20: Plots of the COVID-19 1 week (top left), 2 weeks (top right), 3 weeks (bottom left), and 4 weeks (bottom right) ahead estimates for California (CA).

30

	1 Week Ahead	2 Weeks Ahead	3 Weeks Ahead	4 Weeks Ahead
RMSE				
ARGO	64.91	77.15	102.75	120.04
ARGOX 2Step	59.81	90.78	134.20	240.41
ARGOX NatConstraint	59.83	81.98	121.48	191.13
Ensemble	50.80	61.35	63.80	85.68
Naive	62.56	74.62	94.81	106.19
MAE				
ARGO	40.38	48.24	65.76	78.96
ARGOX 2Step	35.60	50.31	69.10	109.25
ARGOX NatConstraint	42.48	60.01	83.36	124.61
Ensemble	31.78	39.21	43.24	55.66
Naive	35.30	43.32	60.36	69.13
Correlation				
ARGO	0.81	0.75	0.57	0.48
ARGOX 2Step	0.87	0.81	0.69	0.48
ARGOX NatConstraint	0.85	0.75	0.60	0.37
Ensemble	0.90	0.87	0.84	0.74
Naive	0.83	0.76	0.62	0.54

Table S12: Comparison of different methods for state-level COVID-19 1 to 4 weeks ahead incremental death in Colorado (CO). The MSE, MAE, and correlation are reported.

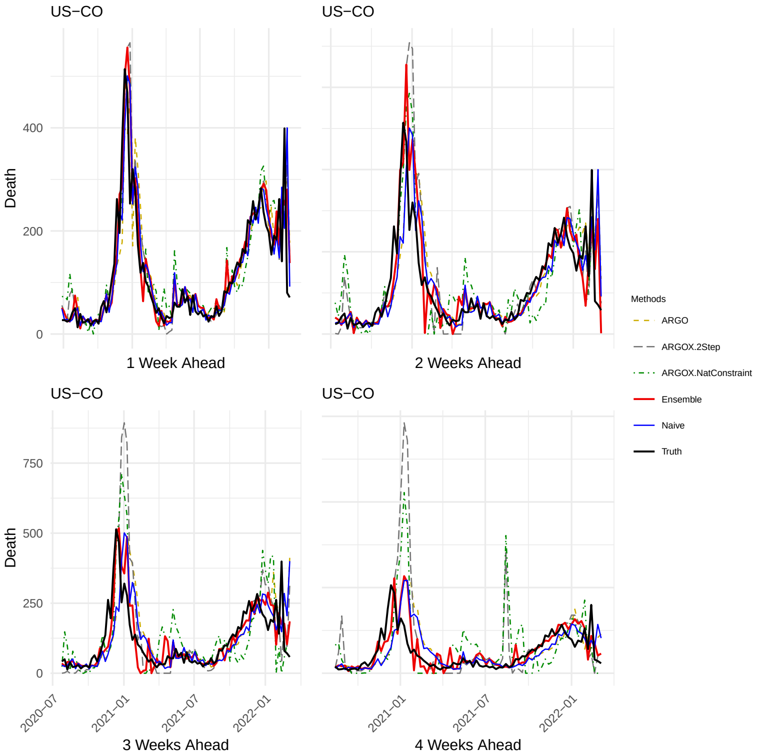

Figure S21: Plots of the COVID-19 1 week (top left), 2 weeks (top right), 3 weeks (bottom left), and 4 weeks (bottom right) ahead estimates for Colorado (CO).

	1 Week Ahead	2 Weeks Ahead	3 Weeks Ahead	4 Weeks Ahead
RMSE				
ARGO	38.68	39.58	52.80	80.65
ARGOX 2Step	35.15	49.01	74.30	129.20
ARGOX NatConstraint	41.40	57.70	67.56	107.71
Ensemble	33.16	34.52	39.02	52.33
Naive	34.70	40.63	54.31	67.26
MAE				
ARGO	23.72	28.89	36.92	55.39
ARGOX 2Step	22.16	31.69	49.06	79.79
ARGOX NatConstraint	29.83	43.25	52.92	78.08
Ensemble	19.50	23.54	26.82	35.30
Naive	21.08	26.83	35.95	47.76
Correlation				
ARGO	0.87	0.86	0.76	0.54
ARGOX 2Step	0.92	0.89	0.82	0.70
ARGOX NatConstraint	0.86	0.72	0.67	0.36
Ensemble	0.92	0.92	0.88	0.80
Naive	0.90	0.86	0.75	0.64

Table S13: Comparison of different methods for state-level COVID-19 1 to 4 weeks ahead incremental death in Connecticut (CT). The MSE, MAE, and correlation are reported.

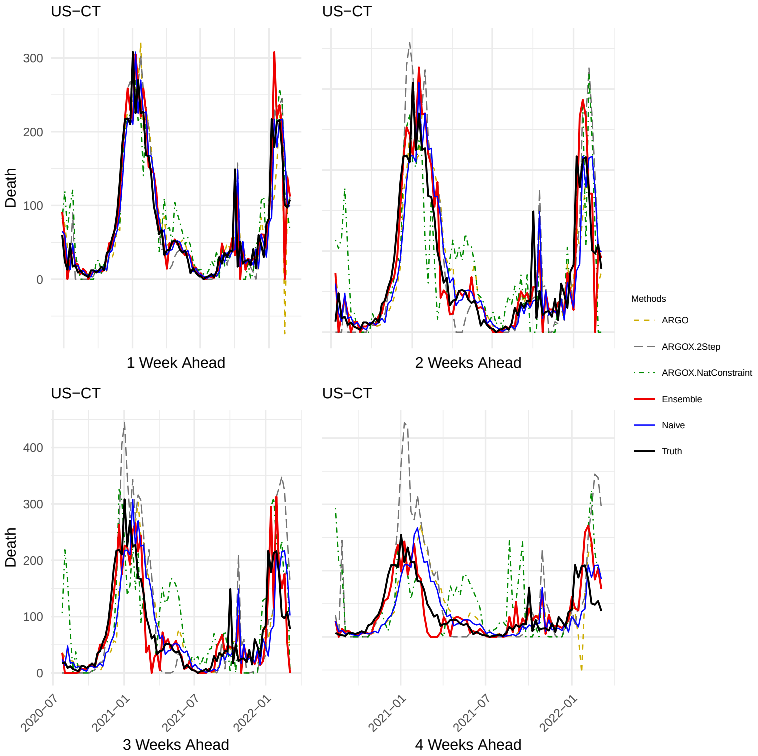

Figure S22: Plots of the COVID-19 1 week (top left), 2 weeks (top right), 3 weeks (bottom left), and 4 weeks (bottom right) ahead estimates for Connecticut (CT).

32

	1 Week Ahead	2 Weeks Ahead	3 Weeks Ahead	4 Weeks Ahead
RMSE				
ARGO	6.06	7.36	8.29	9.76
ARGOX 2Step	6.06	8.06	10.85	14.75
ARGOX NatConstraint	21.29	29.01	50.73	68.05
Ensemble	5.45	6.37	6.59	7.90
Naive	5.78	6.58	7.71	8.51
MAE				
ARGO	4.50	5.26	6.01	7.29
ARGOX 2Step	4.21	5.13	6.84	9.44
ARGOX NatConstraint	15.09	19.98	32.72	39.15
Ensemble	3.86	4.39	4.65	5.49
Naive	4.12	4.52	5.39	5.96
Correlation				
ARGO	0.77	0.67	0.59	0.53
ARGOX 2Step	0.80	0.74	0.67	0.62
ARGOX NatConstraint	0.23	0.07	0.01	0.14
Ensemble	0.83	0.79	0.78	0.74
Naive	0.80	0.74	0.64	0.63

Table S14: Comparison of different methods for state-level COVID-19 1 to 4 weeks ahead incremental death in District of Columbia (DC). The MSE, MAE, and correlation are reported.

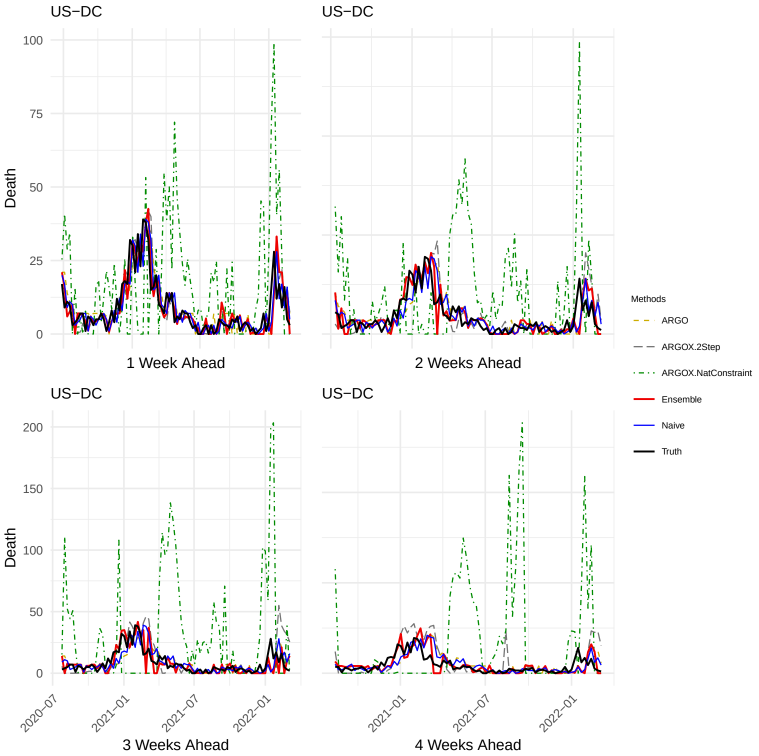

Figure S23: Plots of the COVID-19 1 week (top left), 2 weeks (top right), 3 weeks (bottom left), and 4 weeks (bottom right) ahead estimates for District of Columbia (DC).

	1 Week Ahead	2 Weeks Ahead	3 Weeks Ahead	4 Weeks Ahead
RMSE				
ARGO	32.29	31.21	30.91	34.74
ARGOX 2Step	26.58	27.93	27.04	32.91
ARGOX NatConstraint	37.55	44.83	64.93	72.42
Ensemble	30.10	28.92	31.61	26.21
Naive	28.67	29.60	28.06	31.70
MAE				
ARGO	16.49	16.48	17.04	20.27
ARGOX 2Step	13.17	15.00	14.56	21.04
ARGOX NatConstraint	26.37	32.23	44.59	50.71
Ensemble	16.11	17.30	16.84	18.48
Naive	15.16	16.37	16.65	20.82
Correlation				
ARGO	0.18	0.19	0.22	0.19
ARGOX 2Step	0.37	0.38	0.47	0.37
ARGOX NatConstraint	0.15	0.01	0.00	0.02
Ensemble	0.34	0.30	0.42	0.42
Naive	0.28	0.24	0.30	0.25

Table S15: Comparison of different methods for state-level COVID-19 1 to 4 weeks ahead incremental death in Delaware (DE). The MSE, MAE, and correlation are reported.

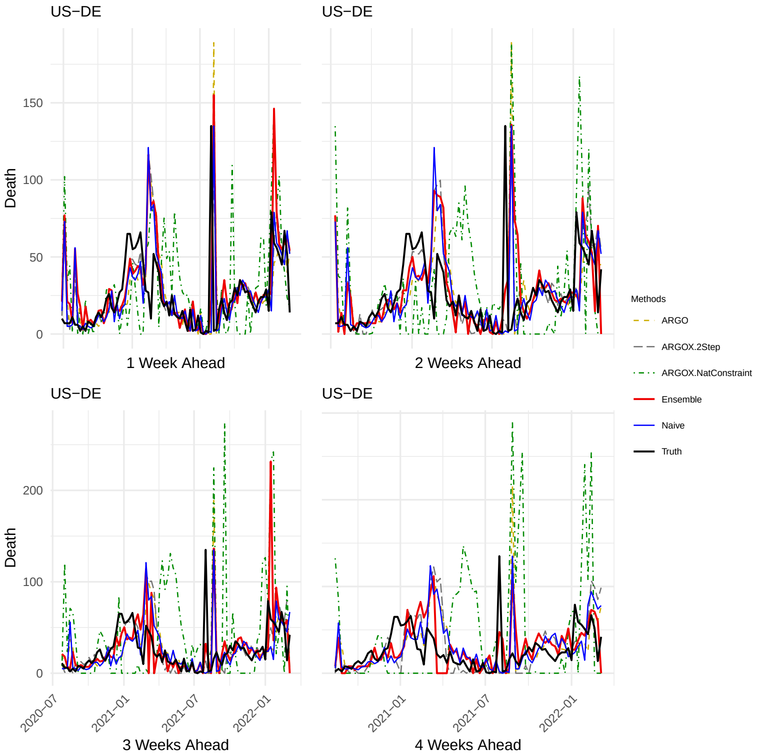

Figure S24: Plots of the COVID-19 1 week (top left), 2 weeks (top right), 3 weeks (bottom left), and 4 weeks (bottom right) ahead estimates for Delaware (DE).

	1 Week Ahead	2 Weeks Ahead	3 Weeks Ahead	4 Weeks Ahead
RMSE				
ARGO	233.92	295.62	374.55	458.43
ARGOX 2Step	201.06	365.45	469.94	923.19
ARGOX NatConstraint	184.53	291.83	350.04	727.36
Ensemble	159.96	265.08	251.38	336.77
Naive	204.46	330.79	461.88	581.77
MAE				
ARGO	159.95	209.98	284.56	357.21
ARGOX 2Step	148.36	250.58	337.74	610.09
ARGOX NatConstraint	141.44	206.99	258.09	489.19
Ensemble	123.82	171.64	186.80	245.60
Naive	148.09	240.51	335.35	436.55
Correlation				
ARGO	0.91	0.83	0.72	0.59
ARGOX 2Step	0.94	0.88	0.80	0.48
ARGOX NatConstraint	0.94	0.87	0.82	0.49
Ensemble	0.95	0.91	0.89	0.79
Naive	0.92	0.80	0.62	0.42

Table S16: Comparison of different methods for state-level COVID-19 1 to 4 weeks ahead incremental death in Florida (FL). The MSE, MAE, and correlation are reported.

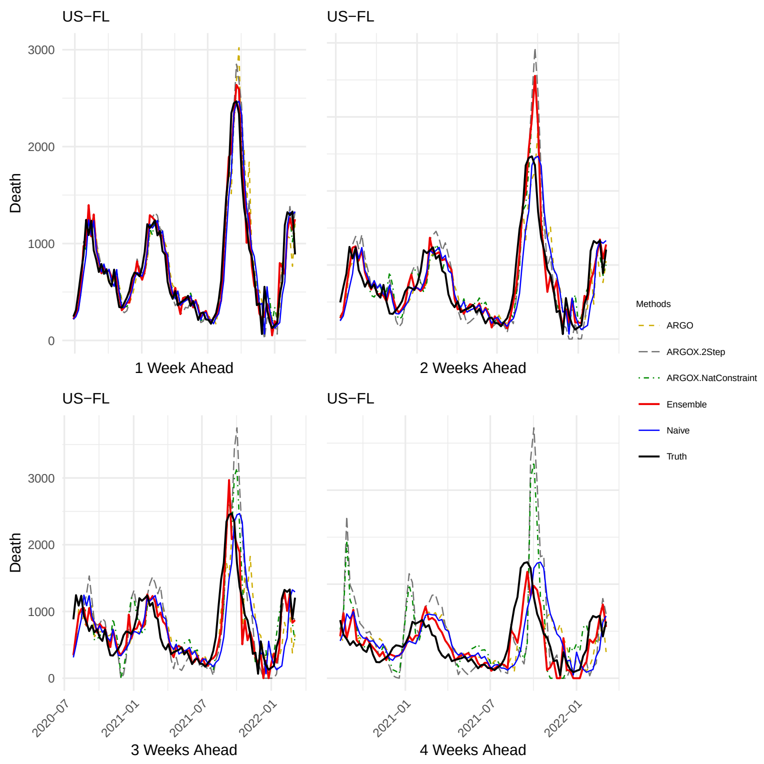

Figure S25: Plots of the COVID-19 1 week (top left), 2 weeks (top right), 3 weeks (bottom left), and 4 weeks (bottom right) ahead estimates for Florida (FL).

35

	1 Week Ahead	2 Weeks Ahead	3 Weeks Ahead	4 Weeks Ahead
RMSE				
ARGO	159.28	180.02	210.70	276.25
ARGOX 2Step	123.19	190.24	260.82	385.47
ARGOX NatConstraint	126.08	176.68	225.48	286.58
Ensemble	110.32	140.65	174.48	212.01
Naive	127.93	168.05	216.75	281.05
MAE				
ARGO	101.30	130.70	157.54	210.15
ARGOX 2Step	88.62	140.75	197.69	305.04
ARGOX NatConstraint	90.70	129.69	161.05	224.07
Ensemble	77.35	98.12	114.00	155.05
Naive	90.32	120.44	160.54	218.66
Correlation				
ARGO	0.77	0.70	0.58	0.40
ARGOX 2Step	0.88	0.78	0.62	0.47
ARGOX NatConstraint	0.86	0.72	0.55	0.45
Ensemble	0.89	0.83	0.73	0.68
Naive	0.86	0.75	0.59	0.38

Table S17: Comparison of different methods for state-level COVID-19 1 to 4 weeks ahead incremental death in Georgia (GA). The MSE, MAE, and correlation are reported.

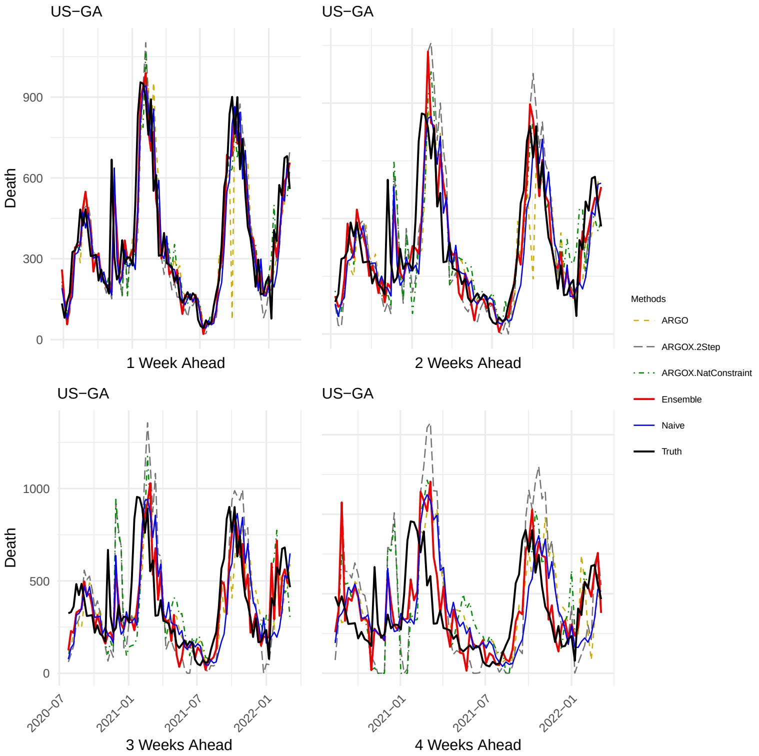

Figure S26: Plots of the COVID-19 1 week (top left), 2 weeks (top right), 3 weeks (bottom left), and 4 weeks (bottom right) ahead estimates for Georgia (GA).

	1 Week Ahead	2 Weeks Ahead	3 Weeks Ahead	4 Weeks Ahead
RMSE				
ARGO	12.19	13.44	15.64	17.02
ARGOX 2Step	10.34	12.03	12.63	13.97
ARGOX NatConstraint	12.19	13.44	15.64	17.02
Ensemble	11.68	12.67	13.05	15.05
Naive	11.67	13.20	14.79	17.63
MAE				
ARGO	7.18	8.92	10.56	11.49
ARGOX 2Step	5.43	7.29	8.16	9.00
ARGOX NatConstraint	7.18	8.92	10.56	11.49
Ensemble	7.05	8.18	8.60	10.01
Naive	6.88	8.14	9.86	11.65
Correlation				
ARGO	0.61	0.53	0.35	0.29
ARGOX 2Step	0.73	0.67	0.62	0.53
ARGOX NatConstraint	0.61	0.53	0.35	0.29
Ensemble	0.68	0.64	0.61	0.51
Naive	0.66	0.56	0.45	0.30

Table S18: Comparison of different methods for state-level COVID-19 1 to 4 weeks ahead incremental death in Hawaii (HI). The MSE, MAE, and correlation are reported.

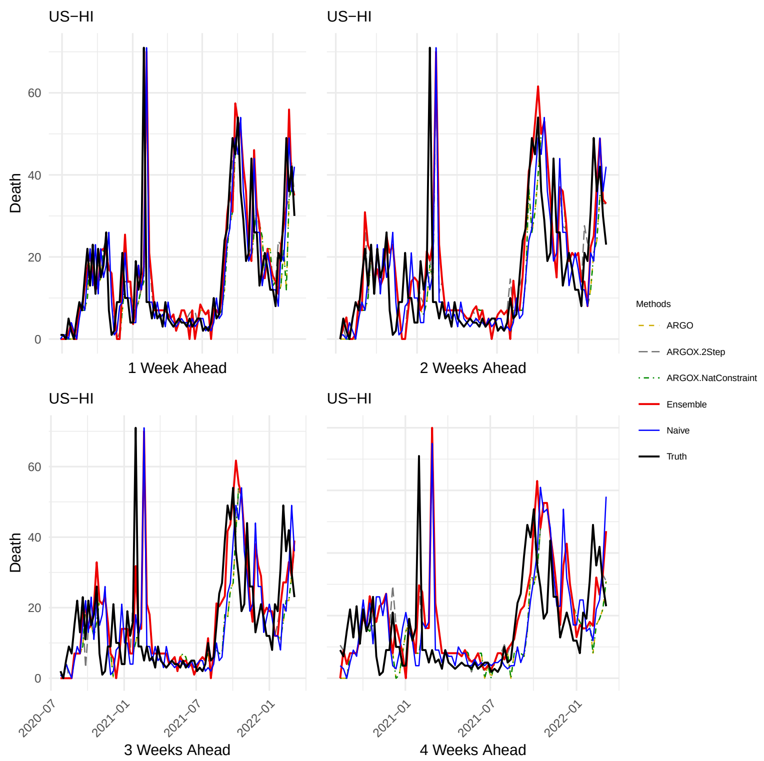

Figure S27: Plots of the COVID-19 1 week (top left), 2 weeks (top right), 3 weeks (bottom left), and 4 weeks (bottom right) ahead estimates for Hawaii (HI).

	1 Week Ahead	2 Weeks Ahead	3 Weeks Ahead	4 Weeks Ahead
RMSE				
ARGO	65.56	76.18	85.55	99.77
ARGOX 2Step	65.28	89.63	111.16	197.94
ARGOX NatConstraint	61.74	85.94	100.09	147.65
Ensemble	55.93	60.78	74.69	84.88
Naive	64.97	78.07	80.49	96.09
MAE				
ARGO	39.45	46.21	46.24	54.88
ARGOX 2Step	34.34	45.63	54.38	87.50
ARGOX NatConstraint	39.42	51.24	67.29	96.91
Ensemble	31.92	34.63	41.21	48.36
Naive	32.25	37.89	44.56	54.10
Correlation				
ARGO	0.74	0.65	0.56	0.48
ARGOX 2Step	0.76	0.68	0.62	0.41
ARGOX NatConstraint	0.75	0.57	0.50	0.26
Ensemble	0.79	0.77	0.71	0.65
Naive	0.74	0.62	0.60	0.49

Table S19: Comparison of different methods for state-level COVID-19 1 to 4 weeks ahead incremental death in Iowa (IA). The MSE, MAE, and correlation are reported.

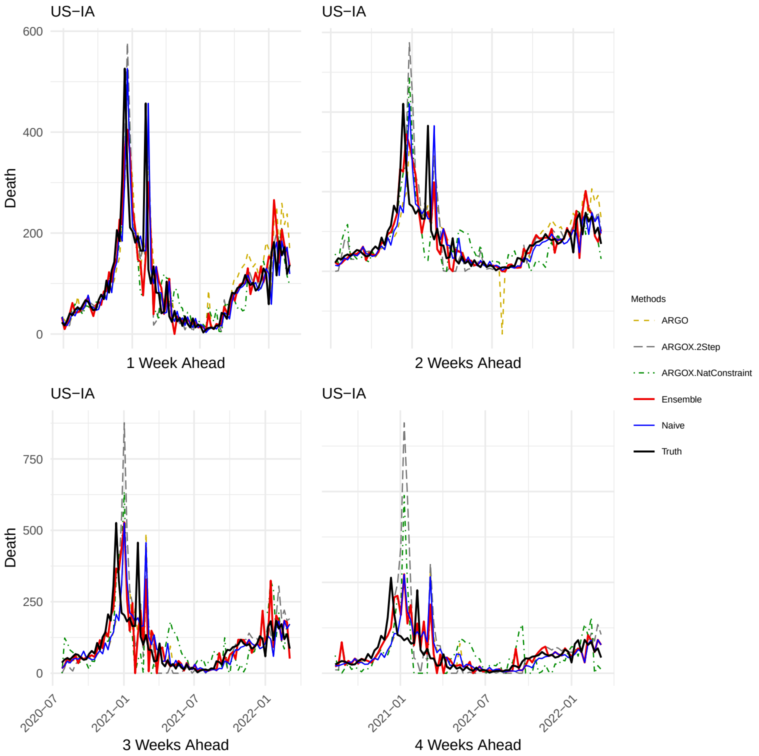

Figure S28: Plots of the COVID-19 1 week (top left), 2 weeks (top right), 3 weeks (bottom left), and 4 weeks (bottom right) ahead estimates for Iowa (IA).

	1 Week Ahead	2 Weeks Ahead	3 Weeks Ahead	4 Weeks Ahead
RMSE				
ARGO	25.28	29.40	35.09	47.02
ARGOX 2Step	26.00	30.15	42.04	72.30
ARGOX NatConstraint	35.23	44.14	70.01	74.43
Ensemble	20.89	25.04	32.32	42.81
Naive	24.22	27.85	31.98	41.45
MAE				
ARGO	16.65	21.41	24.92	33.66
ARGOX 2Step	16.68	21.22	27.87	48.07
ARGOX NatConstraint	23.72	32.81	48.95	59.17
Ensemble	12.71	17.40	20.73	29.70
Naive	16.24	20.25	23.08	28.99
Correlation				
ARGO	0.83	0.76	0.67	0.53
ARGOX 2Step	0.84	0.85	0.75	0.64
ARGOX NatConstraint	0.65	0.46	0.21	0.27
Ensemble	0.88	0.85	0.72	0.69
Naive	0.84	0.79	0.72	0.57

Table S20: Comparison of different methods for state-level COVID-19 1 to 4 weeks ahead incremental death in Idaho (ID). The MSE, MAE, and correlation are reported.

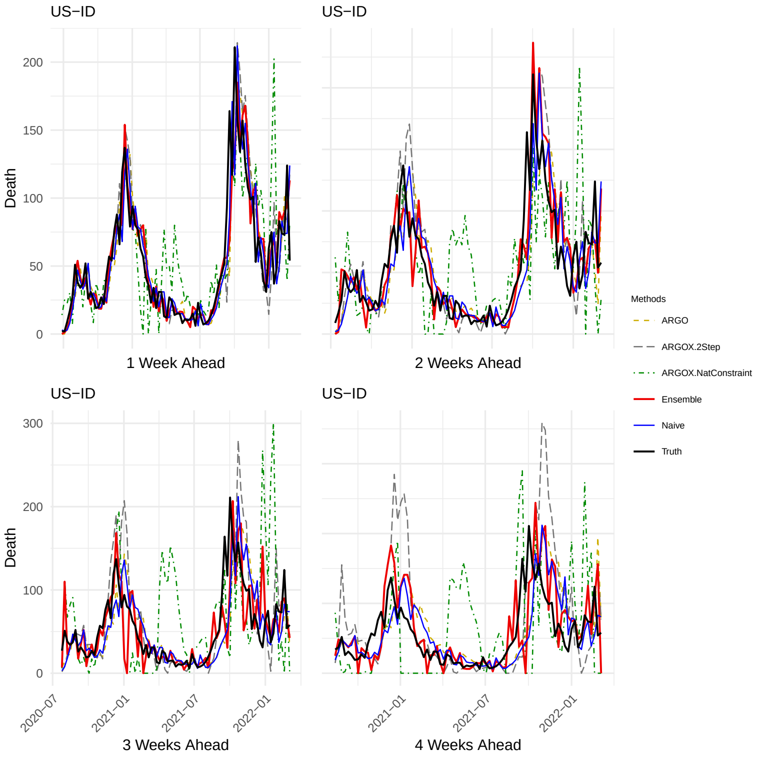

Figure S29: Plots of the COVID-19 1 week (top left), 2 weeks (top right), 3 weeks (bottom left), and 4 weeks (bottom right) ahead estimates for Idaho (ID).

39

	1 Week Ahead	2 Weeks Ahead	3 Weeks Ahead	4 Weeks Ahead
RMSE				
ARGO	128.11	171.08	206.97	262.97
ARGOX 2Step	123.29	225.01	346.47	562.15
ARGOX NatConstraint	99.61	160.64	256.16	430.44
Ensemble	105.05	146.62	160.10	218.96
Naive	97.66	147.50	194.38	263.79
MAE				
ARGO	80.58	107.08	132.61	180.85
ARGOX 2Step	79.16	146.62	212.52	315.17
ARGOX NatConstraint	66.19	99.65	150.27	245.13
Ensemble	65.70	85.74	100.60	139.30
Naive	63.61	99.98	136.62	187.01
Correlation				
ARGO	0.91	0.83	0.75	0.66
ARGOX 2Step	0.95	0.91	0.83	0.70
ARGOX NatConstraint	0.95	0.91	0.84	0.69
Ensemble	0.95	0.92	0.88	0.80
Naive	0.95	0.87	0.78	0.67

Table S21: Comparison of different methods for state-level COVID-19 1 to 4 weeks ahead incremental death in Illinois (IL). The MSE, MAE, and correlation are reported.

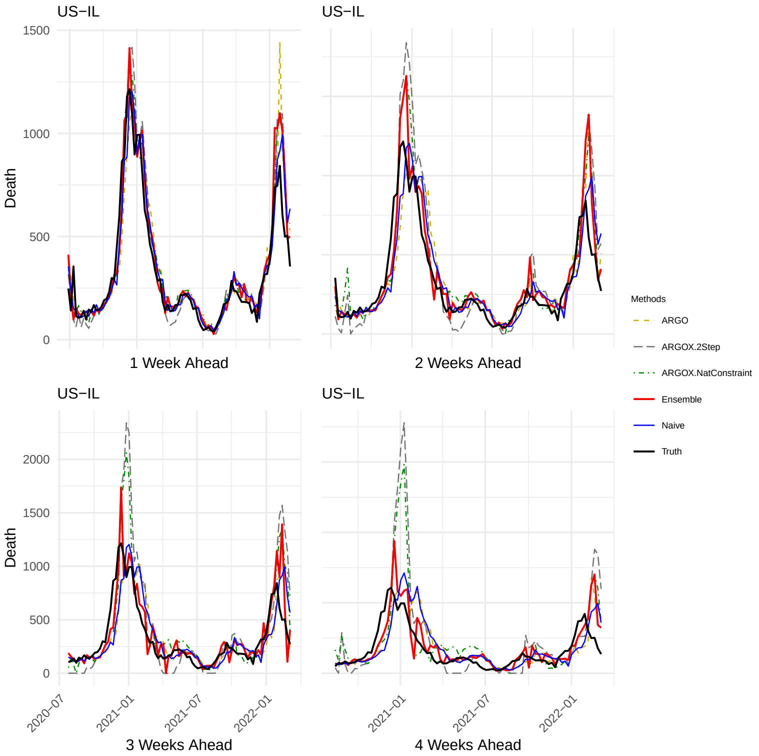

Figure S30: Plots of the COVID-19 1 week (top left), 2 weeks (top right), 3 weeks (bottom left), and 4 weeks (bottom right) ahead estimates for Illinois (IL).

	1 Week Ahead	2 Weeks Ahead	3 Weeks Ahead	4 Weeks Ahead
RMSE				
ARGO	151.78	175.84	184.69	225.99
ARGOX 2Step	164.14	202.95	225.03	304.34
ARGOX NatConstraint	152.41	180.21	179.91	212.94
Ensemble	139.17	157.10	147.22	201.49
Naive	168.47	198.96	212.07	236.93
MAE				
ARGO	78.69	97.36	101.86	132.65
ARGOX 2Step	75.96	107.19	128.88	184.09
ARGOX NatConstraint	74.22	98.14	104.02	142.42
Ensemble	64.33	75.84	72.40	103.82
Naive	67.91	87.55	108.04	134.37
Correlation				
ARGO	0.72	0.63	0.57	0.48
ARGOX 2Step	0.75	0.70	0.70	0.63
ARGOX NatConstraint	0.74	0.66	0.70	0.62
Ensemble	0.78	0.72	0.76	0.69
Naive	0.71	0.58	0.53	0.44

Table S22: Comparison of different methods for state-level COVID-19 1 to 4 weeks ahead incremental death in Indiana (IN). The MSE, MAE, and correlation are reported.

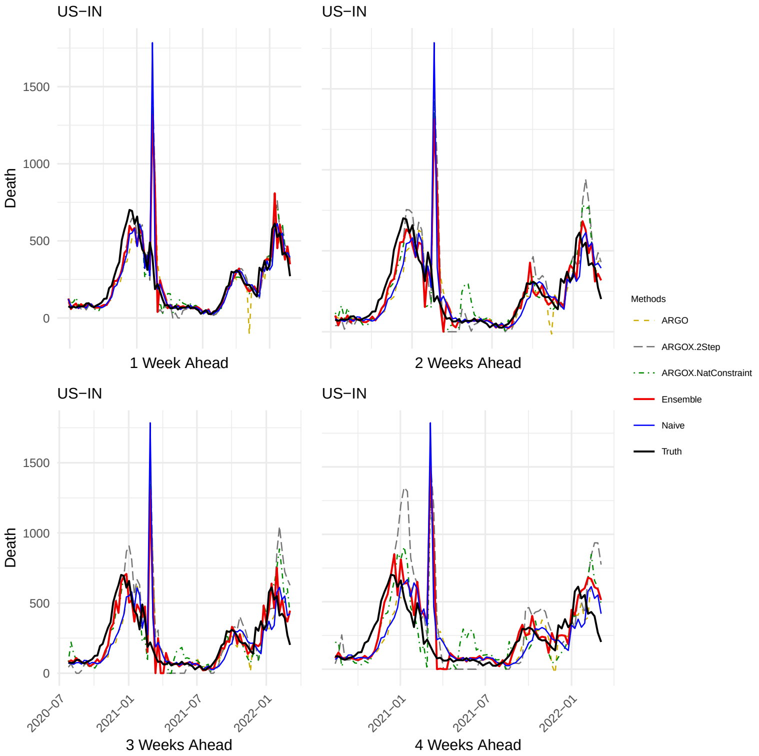

Figure S31: Plots of the COVID-19 1 week (top left), 2 weeks (top right), 3 weeks (bottom left), and 4 weeks (bottom right) ahead estimates for Indiana (IN).

41

	1 Week Ahead	2 Weeks Ahead	3 Weeks Ahead	4 Weeks Ahead
RMSE				
ARGO	61.58	69.11	81.38	99.16
ARGOX 2Step	63.99	74.36	82.23	112.64
ARGOX NatConstraint	66.00	78.83	95.31	100.76
Ensemble	58.30	64.67	56.26	63.29
Naive	64.84	69.08	83.44	82.67
MAE				
ARGO	39.90	46.70	49.87	60.44
ARGOX 2Step	39.34	47.22	53.34	73.33
ARGOX NatConstraint	45.17	56.80	71.91	76.40
Ensemble	36.10	39.83	39.82	44.06
Naive	42.01	46.62	56.09	57.23
Correlation				
ARGO	0.73	0.68	0.55	0.50
ARGOX 2Step	0.75	0.71	0.68	0.67
ARGOX NatConstraint	0.69	0.54	0.37	0.35
Ensemble	0.75	0.72	0.77	0.76
Naive	0.71	0.68	0.53	0.55

Table S23: Comparison of different methods for state-level COVID-19 1 to 4 weeks ahead incremental death in Kansas (KS). The MSE, MAE, and correlation are reported.

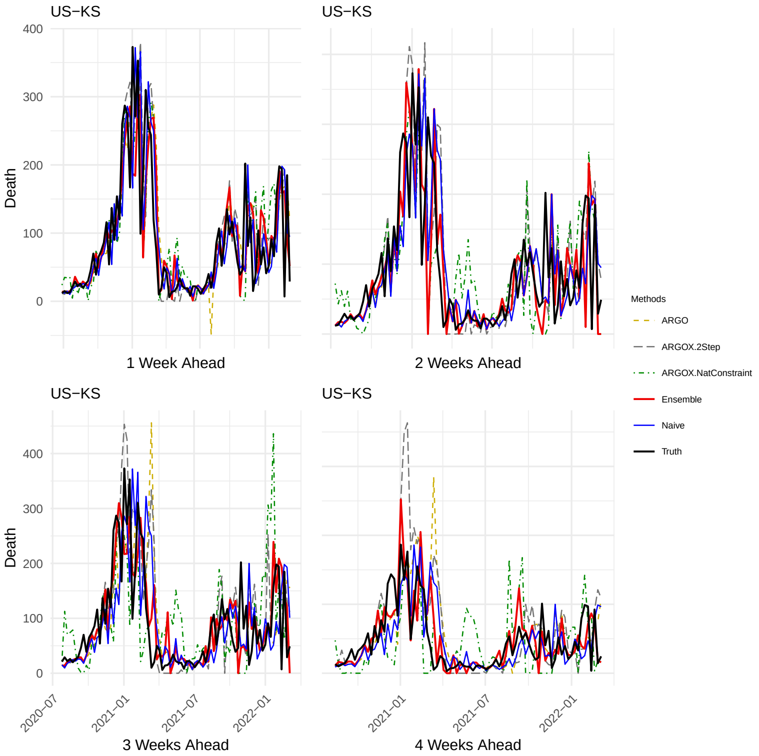

Figure S32: Plots of the COVID-19 1 week (top left), 2 weeks (top right), 3 weeks (bottom left), and 4 weeks (bottom right) ahead estimates for Kansas (KS).

42

	1 Week Ahead	2 Weeks Ahead	3 Weeks Ahead	4 Weeks Ahead
RMSE				
ARGO	96.47	107.73	125.92	170.33
ARGOX 2Step	96.89	119.00	133.75	173.83
ARGOX NatConstraint	102.09	128.56	163.30	193.95
Ensemble	94.59	100.19	107.17	121.67
Naive	95.62	114.25	113.23	127.33
MAE				
ARGO	52.91	60.86	74.42	100.37
ARGOX 2Step	55.28	73.29	90.17	122.63
ARGOX NatConstraint	59.97	78.44	113.27	137.56
Ensemble	51.69	54.75	62.79	79.61
Naive	50.92	62.99	63.24	81.07
Correlation				
ARGO	0.62	0.52	0.43	0.30
ARGOX 2Step	0.63	0.49	0.41	0.33
ARGOX NatConstraint	0.58	0.31	0.11	0.01
Ensemble	0.64	0.58	0.52	0.46
Naive	0.64	0.48	0.49	0.38

Table S24: Comparison of different methods for state-level COVID-19 1 to 4 weeks ahead incremental death in Kentucky (KY). The MSE, MAE, and correlation are reported.

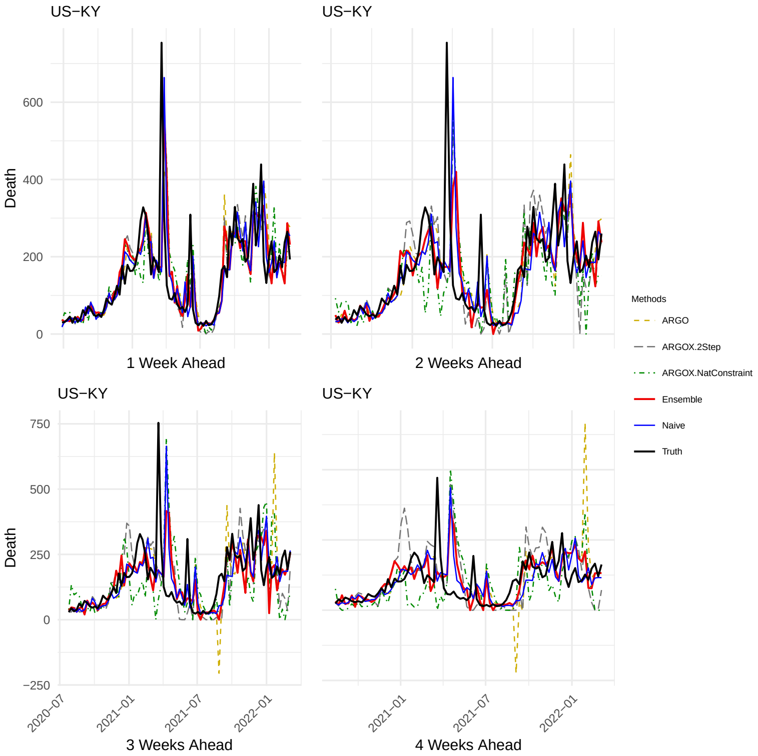

Figure S33: Plots of the COVID-19 1 week (top left), 2 weeks (top right), 3 weeks (bottom left), and 4 weeks (bottom right) ahead estimates for Kentucky (KY).

43

	1 Week Ahead	2 Weeks Ahead	3 Weeks Ahead	4 Weeks Ahead
RMSE				
ARGO	197.98	89.34	116.30	715.59
ARGOX 2Step	60.28	109.33	116.77	212.45
ARGOX NatConstraint	60.04	90.71	107.68	156.67
Ensemble	50.80	68.93	74.37	120.48
Naive	47.95	69.30	95.71	113.14
MAE				
ARGO	56.78	56.89	76.82	199.96
ARGOX 2Step	39.04	65.46	81.72	135.48
ARGOX NatConstraint	39.43	60.94	75.87	102.97
Ensemble	29.65	42.18	49.94	69.03
Naive	33.34	49.13	69.00	82.98
Correlation				
ARGO	0.30	0.69	0.53	0.36
ARGOX 2Step	0.88	0.81	0.71	0.60
ARGOX NatConstraint	0.83	0.75	0.60	0.61
Ensemble	0.88	0.85	0.77	0.70
Naive	0.90	0.78	0.59	0.44

Table S25: Comparison of different methods for state-level COVID-19 1 to 4 weeks ahead incremental death in Louisiana (LA). The MSE, MAE, and correlation are reported.

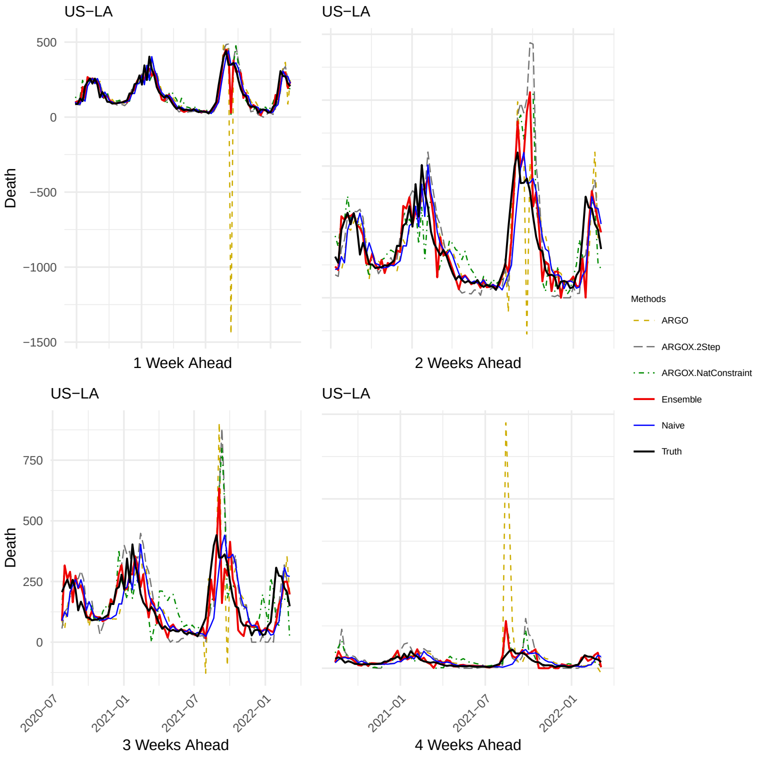

Figure S34: Plots of the COVID-19 1 week (top left), 2 weeks (top right), 3 weeks (bottom left), and 4 weeks (bottom right) ahead estimates for Louisiana (LA).

44

	1 Week Ahead	2 Weeks Ahead	3 Weeks Ahead	4 Weeks Ahead
RMSE				
ARGO	51.88	72.40	99.83	119.41
ARGOX 2Step	46.95	70.34	105.11	169.48
ARGOX NatConstraint	49.98	74.04	95.20	145.60
Ensemble	39.83	46.05	62.44	72.91
Naive	45.93	68.94	89.05	118.44
MAE				
ARGO	36.65	50.01	69.06	84.32
ARGOX 2Step	33.93	49.54	73.40	113.62
ARGOX NatConstraint	34.52	52.03	71.21	108.41
Ensemble	27.42	30.56	41.83	50.66
Naive	33.09	46.00	63.22	85.46
Correlation				
ARGO	0.94	0.88	0.81	0.74
ARGOX 2Step	0.96	0.93	0.88	0.82
ARGOX NatConstraint	0.94	0.87	0.79	0.61
Ensemble	0.97	0.95	0.93	0.92
Naive	0.95	0.89	0.82	0.72

Table S26: Comparison of different methods for state-level COVID-19 1 to 4 weeks ahead incremental death in Massachusetts (MA). The MSE, MAE, and correlation are reported.

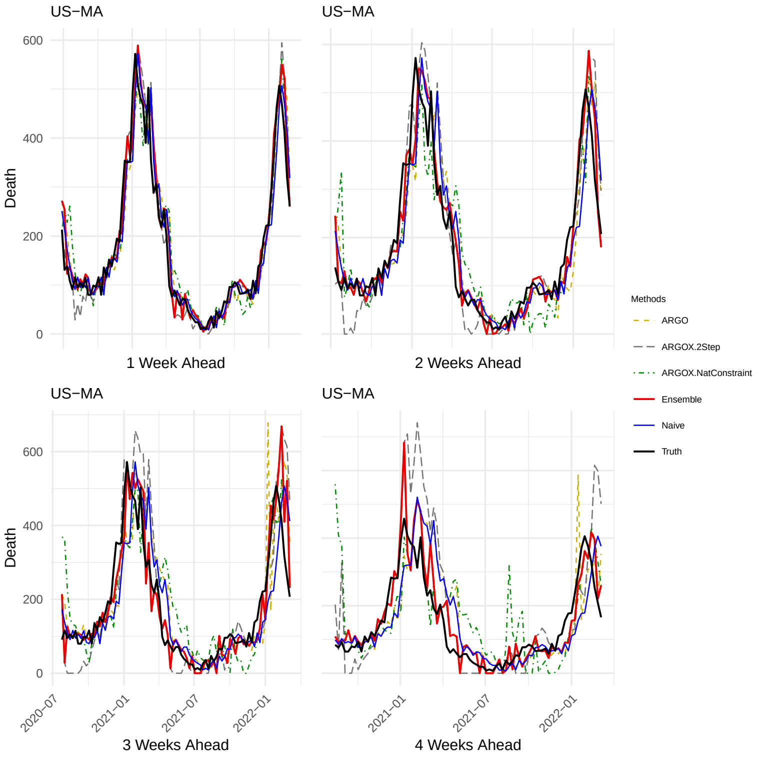

Figure S35: Plots of the COVID-19 1 week (top left), 2 weeks (top right), 3 weeks (bottom left), and 4 weeks (bottom right) ahead estimates for Massachusetts (MA).

	1 Week Ahead	2 Weeks Ahead	3 Weeks Ahead	4 Weeks Ahead
RMSE				
ARGO	101.66	136.61	135.92	162.53
ARGOX 2Step	104.72	161.94	217.90	308.20
ARGOX NatConstraint	104.37	170.15	184.44	253.76
Ensemble	95.72	116.15	117.33	153.16
Naive	105.15	119.11	138.38	161.32
MAE				
ARGO	46.74	70.47	76.67	103.43
ARGOX 2Step	48.66	88.16	125.97	172.31
ARGOX NatConstraint	52.21	91.13	116.12	154.27
Ensemble	40.79	56.96	64.91	93.73
Naive	39.10	55.33	70.13	95.66
Correlation				
ARGO	0.63	0.45	0.37	0.22
ARGOX 2Step	0.67	0.49	0.37	0.23
ARGOX NatConstraint	0.65	0.38	0.39	0.21
Ensemble	0.69	0.58	0.56	0.38
Naive	0.62	0.52	0.37	0.23

Table S27: Comparison of different methods for state-level COVID-19 1 to 4 weeks ahead incremental death in Maryland (MD). The MSE, MAE, and correlation are reported.

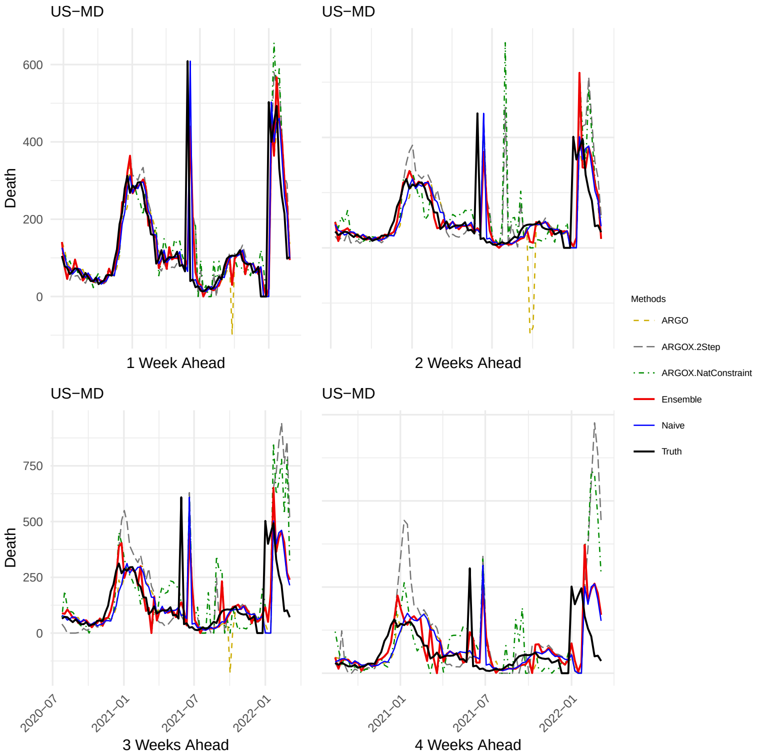

Figure S36: Plots of the COVID-19 1 week (top left), 2 weeks (top right), 3 weeks (bottom left), and 4 weeks (bottom right) ahead estimates for Maryland (MD).

46

	1 Week Ahead	2 Weeks Ahead	3 Weeks Ahead	4 Weeks Ahead
RMSE				
ARGO	17.29	17.69	18.25	20.65
ARGOX 2Step	16.40	17.18	18.79	23.88
ARGOX NatConstraint	30.14	38.41	59.91	69.85
Ensemble	16.83	18.94	20.32	17.73
Naive	19.39	20.22	19.49	21.74
MAE				
ARGO	10.95	11.47	11.81	13.68
ARGOX 2Step	9.57	11.01	11.75	14.75
ARGOX NatConstraint	21.19	27.86	40.63	45.49
Ensemble	10.57	12.62	11.64	11.83
Naive	11.66	12.85	12.75	13.72
Correlation				
ARGO	0.72	0.72	0.70	0.63
ARGOX 2Step	0.76	0.77	0.74	0.68
ARGOX NatConstraint	0.37	0.15	0.12	0.02
Ensemble	0.74	0.70	0.69	0.77
Naive	0.67	0.65	0.66	0.59

Table S28: Comparison of different methods for state-level COVID-19 1 to 4 weeks ahead incremental death in Maine (ME). The MSE, MAE, and correlation are reported.

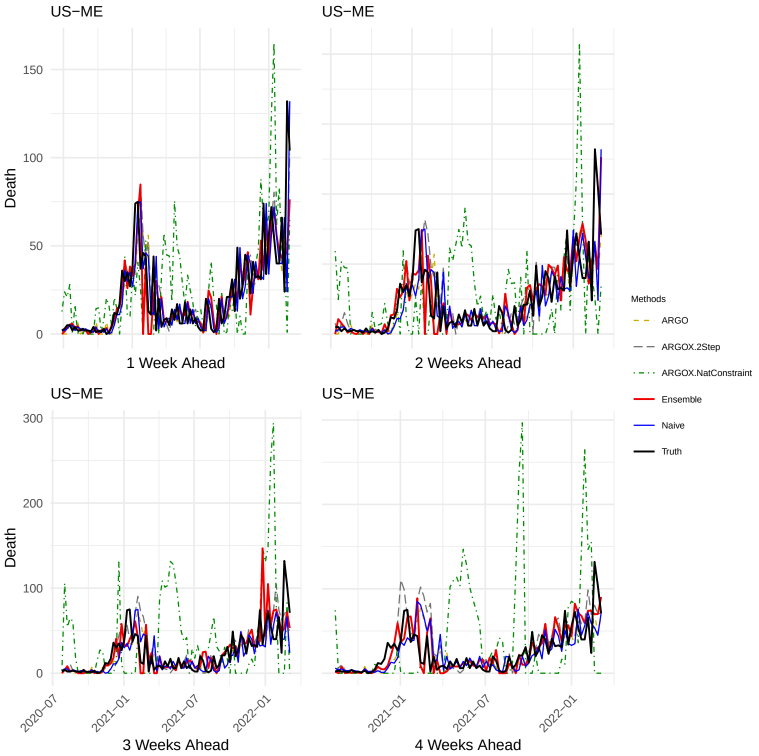

Figure S37: Plots of the COVID-19 1 week (top left), 2 weeks (top right), 3 weeks (bottom left), and 4 weeks (bottom right) ahead estimates for Maine (ME).

	1 Week Ahead	2 Weeks Ahead	3 Weeks Ahead	4 Weeks Ahead
RMSE				
ARGO	144.76	200.23	355.69	301.91
ARGOX 2Step	114.40	174.84	270.06	458.59
ARGOX NatConstraint	103.27	142.30	216.08	362.57
Ensemble	94.31	126.27	150.14	166.86
Naive	103.19	136.68	178.21	257.55
MAE				
ARGO	92.83	132.61	186.48	216.61
ARGOX 2Step	80.07	124.13	180.90	271.68
ARGOX NatConstraint	71.46	97.37	146.66	231.99
Ensemble	61.13	81.18	102.26	121.69
Naive	63.00	99.16	135.73	196.94
Correlation				
ARGO	0.86	0.74	0.44	0.55
ARGOX 2Step	0.93	0.87	0.77	0.60
ARGOX NatConstraint	0.93	0.87	0.77	0.58
Ensemble	0.94	0.90	0.85	0.83
Naive	0.92	0.86	0.77	0.58

Table S29: Comparison of different methods for state-level COVID-19 1 to 4 weeks ahead incremental death in Michigan (MI). The MSE, MAE, and correlation are reported.

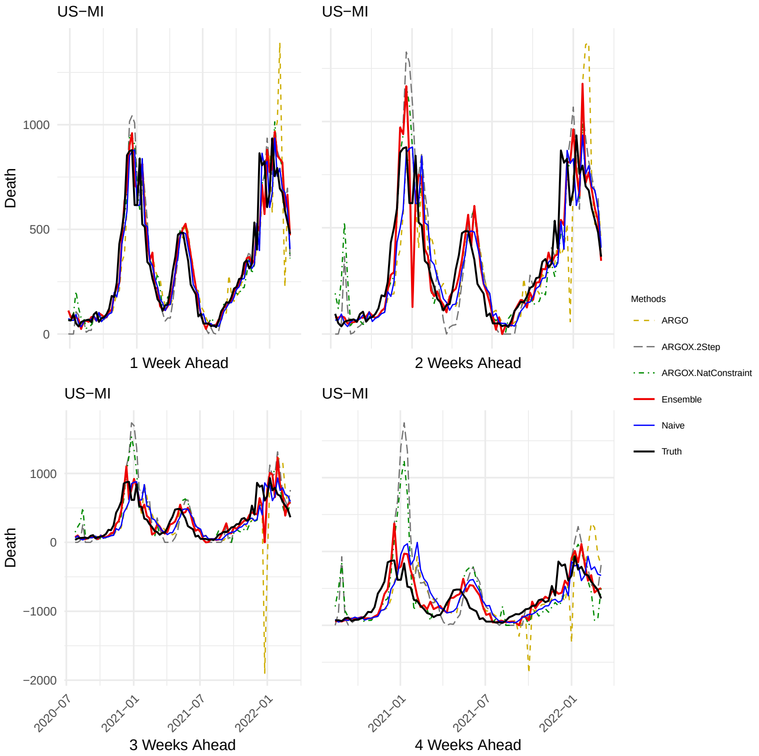

Figure S38: Plots of the COVID-19 1 week (top left), 2 weeks (top right), 3 weeks (bottom left), and 4 weeks (bottom right) ahead estimates for Michigan (MI).

48

	1 Week Ahead	2 Weeks Ahead	3 Weeks Ahead	4 Weeks Ahead
RMSE				
ARGO	47.14	57.65	75.03	96.29
ARGOX 2Step	45.95	71.51	105.64	180.90
ARGOX NatConstraint	47.14	67.37	100.41	138.91
Ensemble	37.56	45.04	48.48	74.31
Naive	38.04	54.05	68.44	92.18
MAE				
ARGO	31.31	41.61	53.19	70.04
ARGOX 2Step	30.80	46.66	61.91	89.36
ARGOX NatConstraint	31.99	50.79	76.88	108.04
Ensemble	23.46	31.57	33.95	50.08
Naive	25.98	37.94	48.56	64.45
Correlation				
ARGO	0.89	0.83	0.73	0.61
ARGOX 2Step	0.93	0.89	0.82	0.69
ARGOX NatConstraint	0.90	0.83	0.71	0.57
Ensemble	0.94	0.92	0.90	0.82
Naive	0.93	0.86	0.77	0.64

Table S30: Comparison of different methods for state-level COVID-19 1 to 4 weeks ahead incremental death in Minnesota (MN). The MSE, MAE, and correlation are reported.

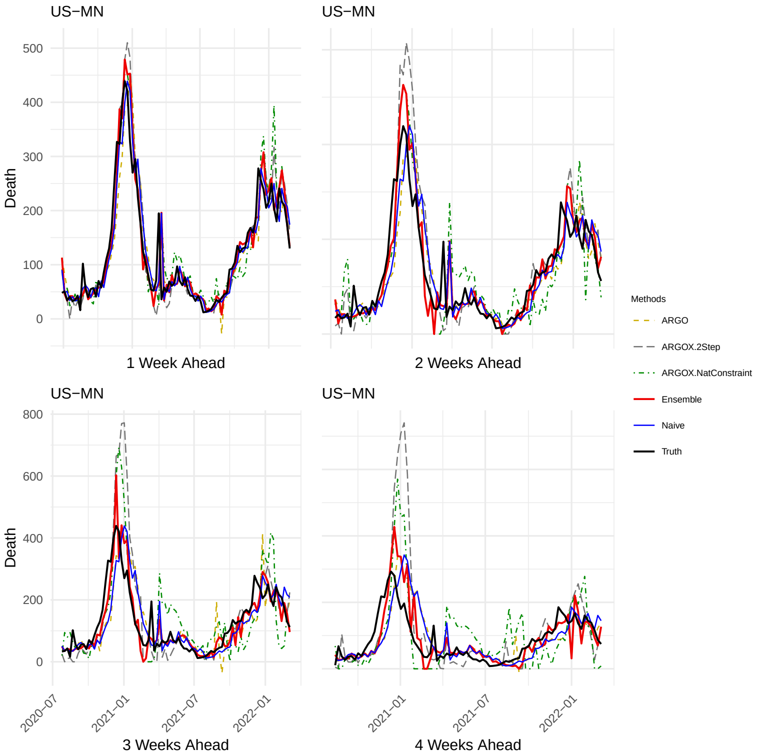

Figure S39: Plots of the COVID-19 1 week (top left), 2 weeks (top right), 3 weeks (bottom left), and 4 weeks (bottom right) ahead estimates for Minnesota (MN).

49

	1 Week Ahead	2 Weeks Ahead	3 Weeks Ahead	4 Weeks Ahead
RMSE				
ARGO	257.15	370.48	357.25	366.17
ARGOX 2Step	263.55	167.86	371.59	379.26
ARGOX NatConstraint	265.51	165.97	388.96	365.65
Ensemble	258.10	153.84	357.51	334.73
Naive	358.89	83.01	357.61	366.20
MAE				
ARGO	90.93	117.00	119.78	136.62
ARGOX 2Step	107.74	100.09	159.49	187.90
ARGOX NatConstraint	107.86	96.14	159.18	184.43
Ensemble	91.45	75.85	112.85	123.86
Naive	109.40	61.37	121.39	135.73
Correlation				
ARGO	0.45	0.45	0.09	0.08
ARGOX 2Step	0.48	0.80	0.11	0.10
ARGOX NatConstraint	0.45	0.81	0.06	0.01
Ensemble	0.44	0.84	0.09	0.10
Naive	0.07	0.96	0.09	0.08

Table S31: Comparison of different methods for state-level COVID-19 1 to 4 weeks ahead incremental death in Missouri (MO). The MSE, MAE, and correlation are reported.

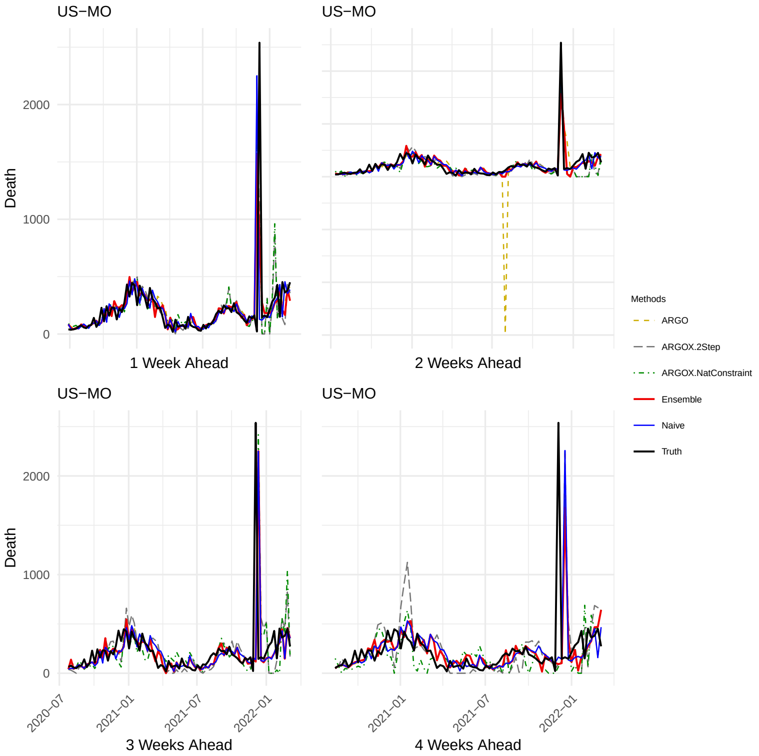

Figure S40: Plots of the COVID-19 1 week (top left), 2 weeks (top right), 3 weeks (bottom left), and 4 weeks (bottom right) ahead estimates for Missouri (MO).

50

	1 Week Ahead	2 Weeks Ahead	3 Weeks Ahead	4 Weeks Ahead
RMSE				
ARGO	51.35	74.19	87.66	109.55
ARGOX 2Step	59.96	78.70	87.27	171.83
ARGOX NatConstraint	57.76	65.80	87.40	107.51
Ensemble	47.50	57.40	64.74	70.47
Naive	51.95	72.23	89.17	107.83
MAE				
ARGO	31.70	50.00	63.15	80.39
ARGOX 2Step	37.48	52.30	63.69	118.98
ARGOX NatConstraint	35.24	47.45	62.63	81.44
Ensemble	27.05	37.38	41.70	51.16
Naive	32.08	47.39	62.33	78.76
Correlation				
ARGO	0.85	0.72	0.60	0.43
ARGOX 2Step	0.83	0.79	0.75	0.53
ARGOX NatConstraint	0.80	0.75	0.58	0.52
Ensemble	0.88	0.84	0.79	0.78
Naive	0.85	0.71	0.56	0.41

Table S32: Comparison of different methods for state-level COVID-19 1 to 4 weeks ahead incremental death in Mississippi (MS). The MSE, MAE, and correlation are reported.

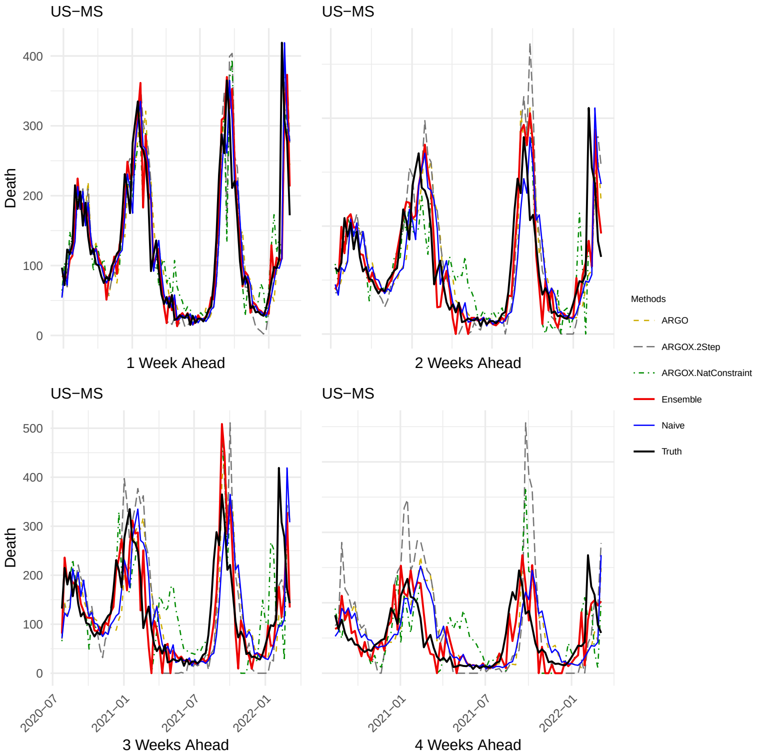

Figure S41: Plots of the COVID-19 1 week (top left), 2 weeks (top right), 3 weeks (bottom left), and 4 weeks (bottom right) ahead estimates for Mississippi (MS).

51

	1 Week Ahead	2 Weeks Ahead	3 Weeks Ahead	4 Weeks Ahead
RMSE				
ARGO	25.01	24.61	28.01	47.68
ARGOX 2Step	19.24	27.82	34.90	53.08
ARGOX NatConstraint	32.06	44.96	67.63	78.73
Ensemble	19.31	23.55	22.56	33.21
Naive	18.28	24.60	27.03	31.72
MAE				
ARGO	15.20	17.78	19.28	24.84
ARGOX 2Step	12.82	20.01	24.62	33.90
ARGOX NatConstraint	22.25	34.52	48.46	57.22
Ensemble	12.90	17.17	14.74	20.73
Naive	12.40	17.45	18.49	22.27
Correlation				
ARGO	0.68	0.62	0.53	0.27
ARGOX 2Step	0.80	0.64	0.59	0.47
ARGOX NatConstraint	0.48	0.10	0.04	0.17
Ensemble	0.79	0.67	0.71	0.58
Naive	0.80	0.64	0.56	0.48

Table S33: Comparison of different methods for state-level COVID-19 1 to 4 weeks ahead incremental death in Montana (MT). The MSE, MAE, and correlation are reported.

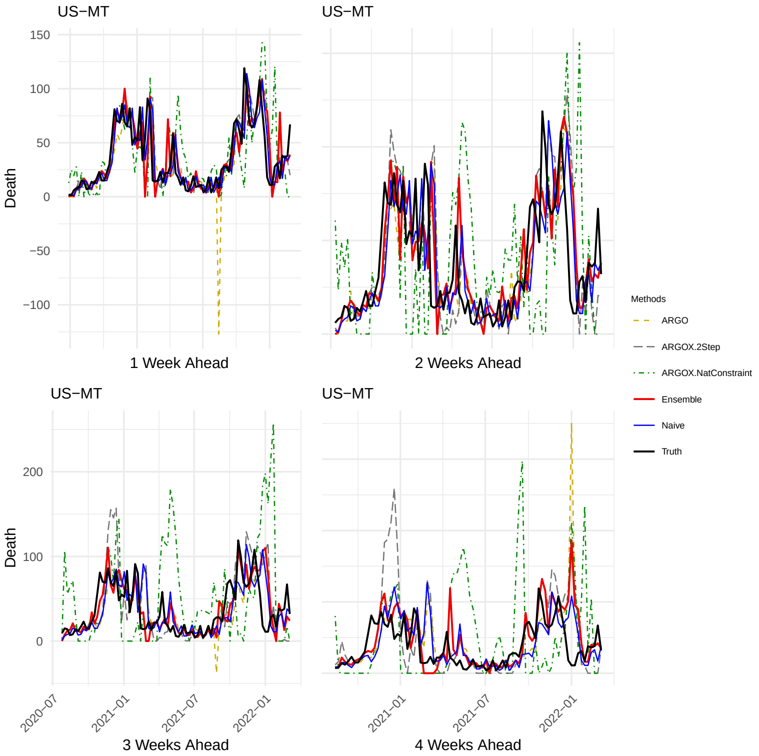

Figure S42: Plots of the COVID-19 1 week (top left), 2 weeks (top right), 3 weeks (bottom left), and 4 weeks (bottom right) ahead estimates for Montana (MT).

52

	1 Week Ahead	2 Weeks Ahead	3 Weeks Ahead	4 Weeks Ahead
RMSE				
ARGO	66.85	81.43	112.84	150.09
ARGOX 2Step	70.10	103.13	139.63	226.69
ARGOX NatConstraint	67.82	84.49	105.70	122.94
Ensemble	55.57	68.61	76.91	97.92
Naive	68.28	97.40	129.83	161.52
MAE				
ARGO	49.64	59.67	80.79	109.05
ARGOX 2Step	52.35	73.66	97.27	163.68
ARGOX NatConstraint	49.56	63.31	82.79	95.72
Ensemble	42.75	50.19	55.92	71.01
Naive	50.43	72.85	95.95	120.02
Correlation				
ARGO	0.91	0.86	0.72	0.58
ARGOX 2Step	0.92	0.86	0.75	0.63
ARGOX NatConstraint	0.90	0.85	0.75	0.71
Ensemble	0.94	0.91	0.88	0.87
Naive	0.91	0.81	0.67	0.53

Table S34: Comparison of different methods for state-level COVID-19 1 to 4 weeks ahead incremental death in North Carolina (NC). The MSE, MAE, and correlation are reported.

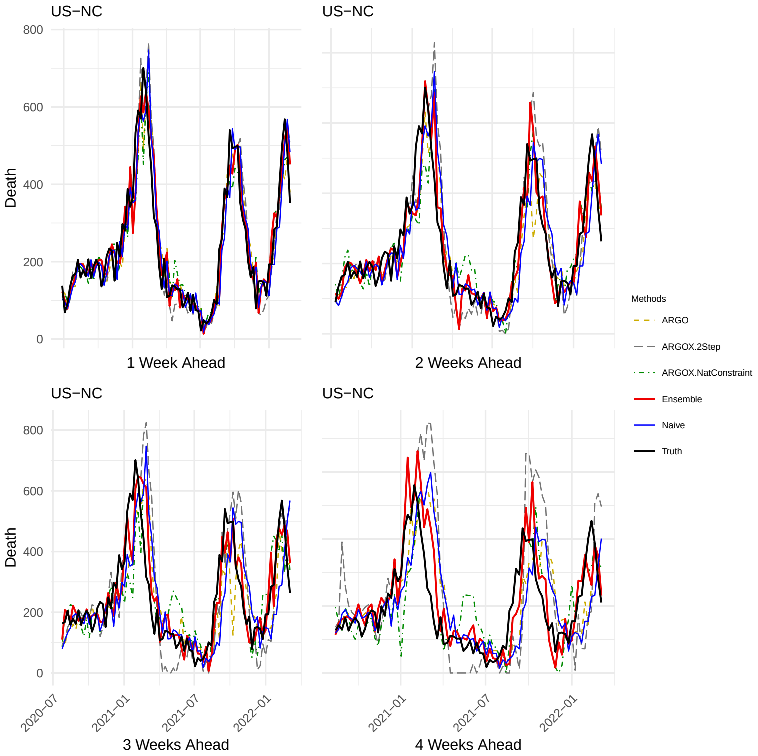

Figure S43: Plots of the COVID-19 1 week (top left), 2 weeks (top right), 3 weeks (bottom left), and 4 weeks (bottom right) ahead estimates for North Carolina (NC).

53

	1 Week Ahead	2 Weeks Ahead	3 Weeks Ahead	4 Weeks Ahead
RMSE				
ARGO	14.06	16.38	20.01	29.29
ARGOX 2Step	12.65	17.59	17.97	29.36
ARGOX NatConstraint	25.10	34.26	58.46	69.17
Ensemble	11.26	16.50	14.45	21.28
Naive	13.42	18.39	19.83	24.86
MAE				
ARGO	8.99	10.46	13.15	17.83
ARGOX 2Step	7.66	10.76	12.61	17.65
ARGOX NatConstraint	18.83	25.63	40.94	46.87
Ensemble	7.46	9.81	9.78	13.36
Naive	7.98	10.08	12.56	15.79
Correlation				
ARGO	0.88	0.82	0.77	0.61
ARGOX 2Step	0.91	0.84	0.88	0.82
ARGOX NatConstraint	0.66	0.42	0.20	0.04
Ensemble	0.92	0.84	0.89	0.80
Naive	0.89	0.79	0.76	0.70

Table S35: Comparison of different methods for state-level COVID-19 1 to 4 weeks ahead incremental death in North Dakota (ND). The MSE, MAE, and correlation are reported.

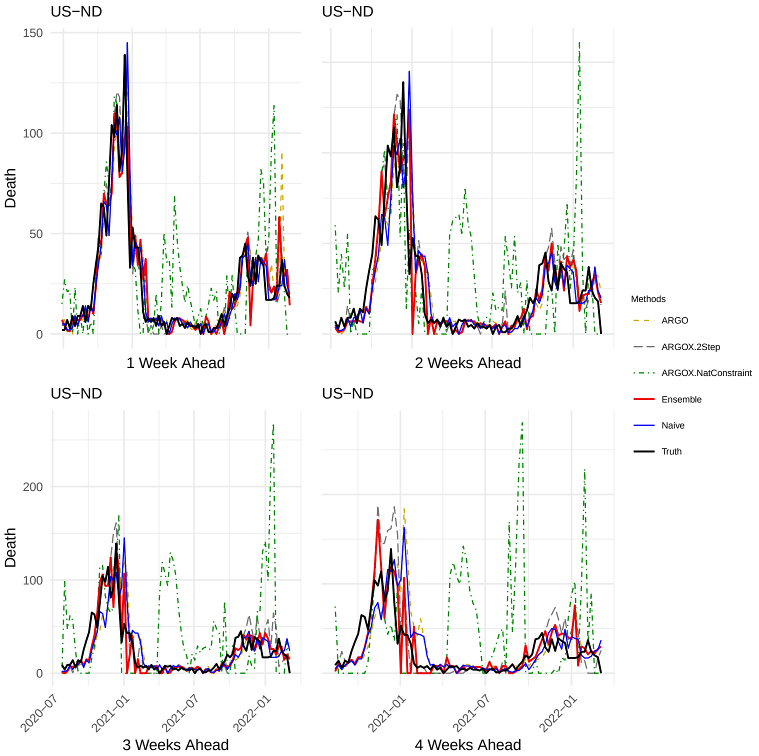

Figure S44: Plots of the COVID-19 1 week (top left), 2 weeks (top right), 3 weeks (bottom left), and 4 weeks (bottom right) ahead estimates for North Dakota (ND).

	1 Week Ahead	2 Weeks Ahead	3 Weeks Ahead	4 Weeks Ahead
RMSE				
ARGO	57.04	58.25	60.46	67.70
ARGOX 2Step	57.78	58.89	66.10	92.81
ARGOX NatConstraint	59.80	72.98	82.97	98.34
Ensemble	56.45	55.93	61.17	68.13
Naive	58.63	61.15	62.37	64.58
MAE				
ARGO	27.33	29.82	31.47	38.51
ARGOX 2Step	25.79	28.00	31.74	43.01
ARGOX NatConstraint	32.51	47.07	54.26	68.32
Ensemble	26.32	28.60	30.29	36.86
Naive	28.18	30.66	31.76	35.49
Correlation				
ARGO	0.38	0.36	0.33	0.25
ARGOX 2Step	0.41	0.45	0.41	0.31
ARGOX NatConstraint	0.36	0.20	0.15	0.02
Ensemble	0.44	0.47	0.43	0.34
Naive	0.36	0.31	0.29	0.27

Table S36: Comparison of different methods for state-level COVID-19 1 to 4 weeks ahead incremental death in Nebraska (NE). The MSE, MAE, and correlation are reported.

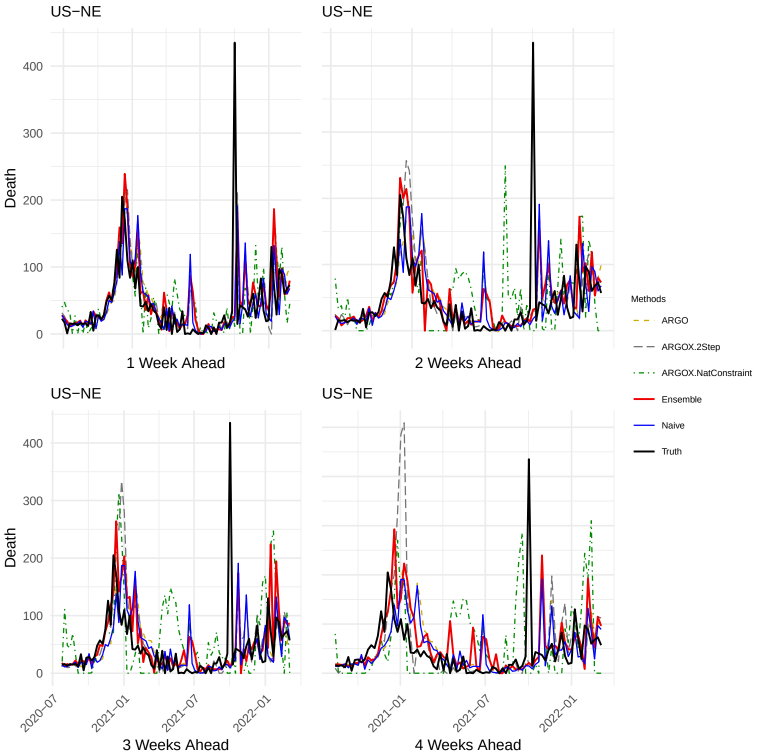

Figure S45: Plots of the COVID-19 1 week (top left), 2 weeks (top right), 3 weeks (bottom left), and 4 weeks (bottom right) ahead estimates for Nebraska (NE).

55

	1 Week Ahead	2 Weeks Ahead	3 Weeks Ahead	4 Weeks Ahead
RMSE				
ARGO	12.36	15.12	16.22	20.10
ARGOX 2Step	12.07	13.75	18.55	29.02
ARGOX NatConstraint	25.60	36.88	55.86	70.97
Ensemble	10.80	14.27	13.37	15.77
Naive	11.87	12.94	16.06	18.99
MAE				
ARGO	9.25	11.00	11.53	14.01
ARGOX 2Step	8.28	10.17	13.47	18.67
ARGOX NatConstraint	19.14	26.69	40.20	47.65
Ensemble	7.69	9.46	9.37	11.30
Naive	8.12	8.93	11.21	13.16
Correlation				
ARGO	0.85	0.77	0.72	0.66
ARGOX 2Step	0.87	0.84	0.78	0.72
ARGOX NatConstraint	0.58	0.23	0.17	0.05
Ensemble	0.89	0.82	0.85	0.83
Naive	0.85	0.82	0.73	0.67

Table S37: Comparison of different methods for state-level COVID-19 1 to 4 weeks ahead incremental death in New Hampshire (NH). The MSE, MAE, and correlation are reported.

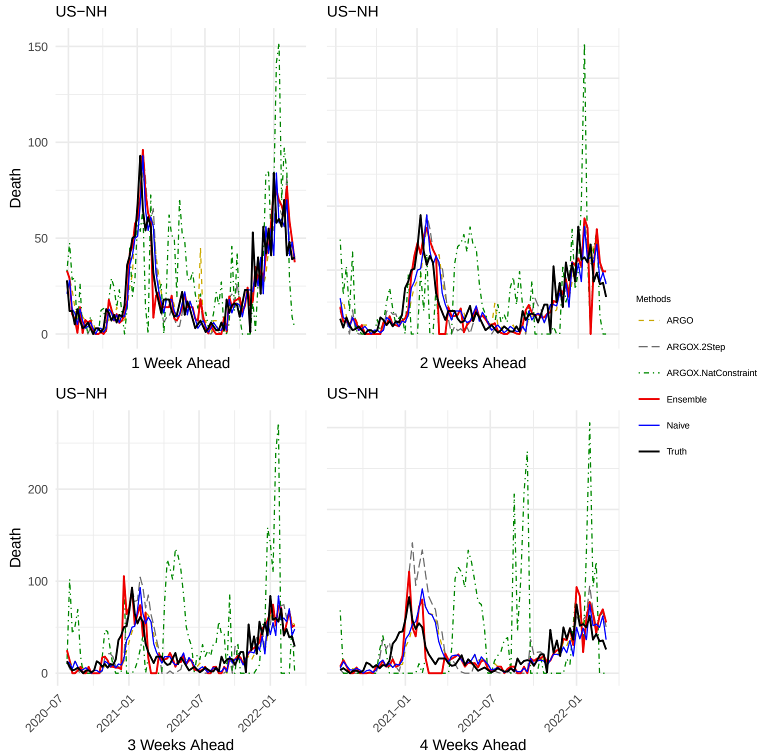

Figure S46: Plots of the COVID-19 1 week (top left), 2 weeks (top right), 3 weeks (bottom left), and 4 weeks (bottom right) ahead estimates for New Hampshire (NH).

	1 Week Ahead	2 Weeks Ahead	3 Weeks Ahead	4 Weeks Ahead
RMSE				
ARGO	70.02	100.39	124.99	161.71
ARGOX 2Step	72.30	122.59	194.68	314.83
ARGOX NatConstraint	73.06	114.66	182.25	270.20
Ensemble	55.36	72.99	86.47	134.70
Naive	61.30	103.48	137.53	177.95
MAE				
ARGO	48.55	69.61	88.28	115.94
ARGOX 2Step	48.14	76.00	113.47	187.03
ARGOX NatConstraint	46.18	72.11	109.53	161.04
Ensemble	37.59	50.25	56.63	86.73
Naive	38.75	65.78	90.95	121.10
Correlation				
ARGO	0.93	0.85	0.77	0.66
ARGOX 2Step	0.94	0.87	0.78	0.64
ARGOX NatConstraint	0.93	0.82	0.68	0.49
Ensemble	0.96	0.93	0.90	0.80
Naive	0.94	0.84	0.72	0.59

Table S38: Comparison of different methods for state-level COVID-19 1 to 4 weeks ahead incremental death in New Jersey (NJ). The MSE, MAE, and correlation are reported.

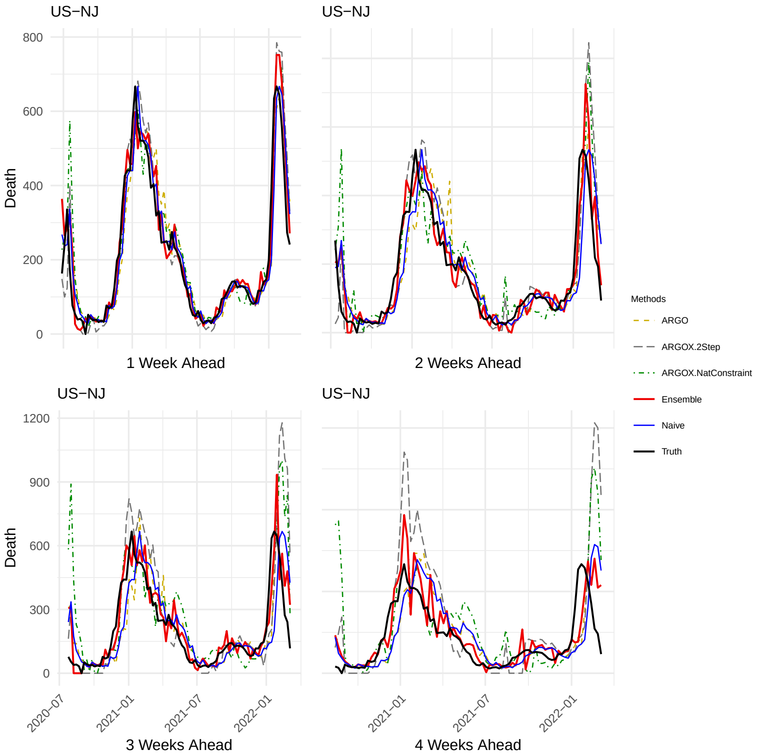

Figure S47: Plots of the COVID-19 1 week (top left), 2 weeks (top right), 3 weeks (bottom left), and 4 weeks (bottom right) ahead estimates for New Jersey (NJ).

57

	1 Week Ahead	2 Weeks Ahead	3 Weeks Ahead	4 Weeks Ahead
RMSE				
ARGO	29.96	34.32	41.14	55.46
ARGOX 2Step	30.78	40.83	59.66	146.43
ARGOX NatConstraint	38.49	55.48	73.95	147.46
Ensemble	26.42	28.95	33.80	44.61
Naive	30.69	32.55	38.78	51.32
MAE				
ARGO	20.15	24.08	29.54	39.51
ARGOX 2Step	19.34	26.61	39.45	70.11
ARGOX NatConstraint	29.31	43.00	57.56	85.85
Ensemble	16.64	19.40	23.65	31.26
Naive	20.10	20.97	27.84	36.22
Correlation				
ARGO	0.87	0.83	0.76	0.67
ARGOX 2Step	0.89	0.88	0.84	0.52
ARGOX NatConstraint	0.79	0.63	0.55	0.17
Ensemble	0.90	0.89	0.86	0.83
Naive	0.86	0.85	0.79	0.67

Table S39: Comparison of different methods for state-level COVID-19 1 to 4 weeks ahead incremental death in New Mexico (NM). The MSE, MAE, and correlation are reported.

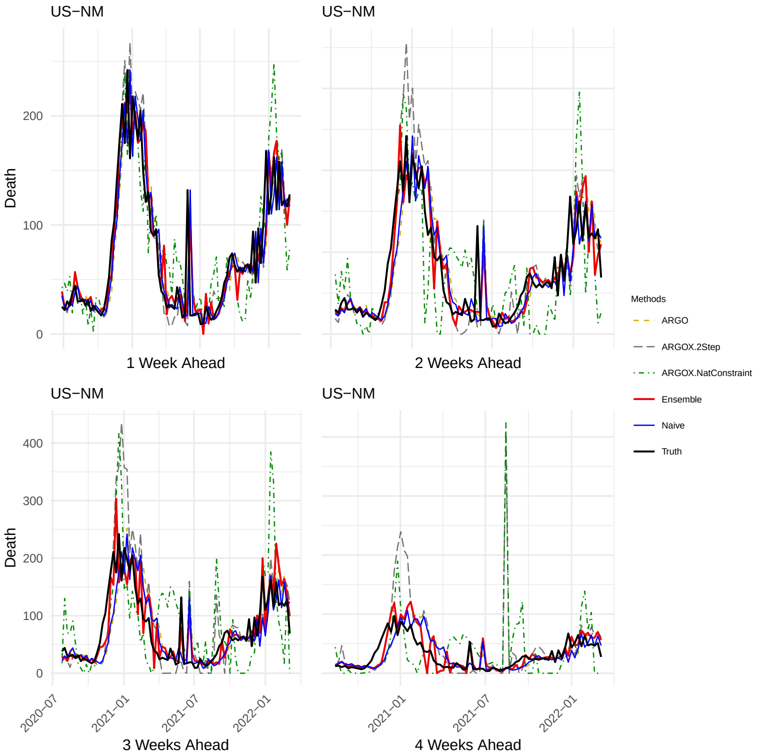

Figure S48: Plots of the COVID-19 1 week (top left), 2 weeks (top right), 3 weeks (bottom left), and 4 weeks (bottom right) ahead estimates for New Mexico (NM).

	1 Week Ahead	2 Weeks Ahead	3 Weeks Ahead	4 Weeks Ahead
RMSE				
ARGO	30.65	45.48	51.50	70.54
ARGOX 2Step	33.23	52.36	69.13	122.88
ARGOX NatConstraint	36.33	47.34	77.08	89.59
Ensemble	27.46	33.65	43.06	49.05
Naive	29.70	42.03	52.54	69.55
MAE				
ARGO	24.10	34.17	38.85	51.94
ARGOX 2Step	25.18	38.24	49.45	85.11
ARGOX NatConstraint	28.66	35.67	57.47	72.99
Ensemble	21.26	24.98	29.46	37.72
Naive	23.07	31.82	39.38	52.13
Correlation				
ARGO	0.90	0.78	0.70	0.54
ARGOX 2Step	0.91	0.85	0.78	0.69
ARGOX NatConstraint	0.85	0.73	0.47	0.47
Ensemble	0.92	0.89	0.83	0.81
Naive	0.91	0.81	0.70	0.55

Table S40: Comparison of different methods for state-level COVID-19 1 to 4 weeks ahead incremental death in Nevada (NV). The MSE, MAE, and correlation are reported.

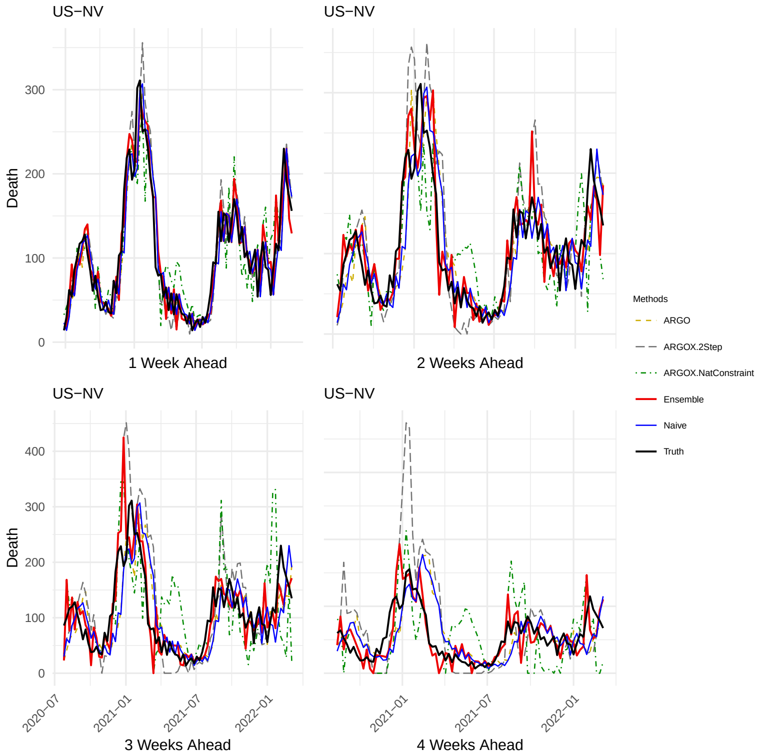

Figure S49: Plots of the COVID-19 1 week (top left), 2 weeks (top right), 3 weeks (bottom left), and 4 weeks (bottom right) ahead estimates for Nevada (NV).

	1 Week Ahead	2 Weeks Ahead	3 Weeks Ahead	4 Weeks Ahead
RMSE				
ARGO	128.69	192.40	253.99	358.30
ARGOX 2Step	130.47	255.84	413.06	597.82
ARGOX NatConstraint	136.36	283.21	373.61	449.81
Ensemble	102.58	143.31	186.08	292.33
Naive	132.02	211.37	288.52	370.52
MAE				
ARGO	81.47	132.32	174.44	245.22
ARGOX 2Step	84.84	147.29	221.41	337.03
ARGOX NatConstraint	82.50	157.43	217.86	278.27
Ensemble	63.76	94.56	116.47	176.47
Naive	77.43	136.72	189.99	249.82
Correlation				
ARGO	0.94	0.88	0.78	0.64
ARGOX 2Step	0.96	0.88	0.75	0.66
ARGOX NatConstraint	0.94	0.78	0.68	0.63
Ensemble	0.97	0.94	0.89	0.80
Naive	0.94	0.86	0.73	0.62

Table S41: Comparison of different methods for state-level COVID-19 1 to 4 weeks ahead incremental death in New York (NY). The MSE, MAE, and correlation are reported.

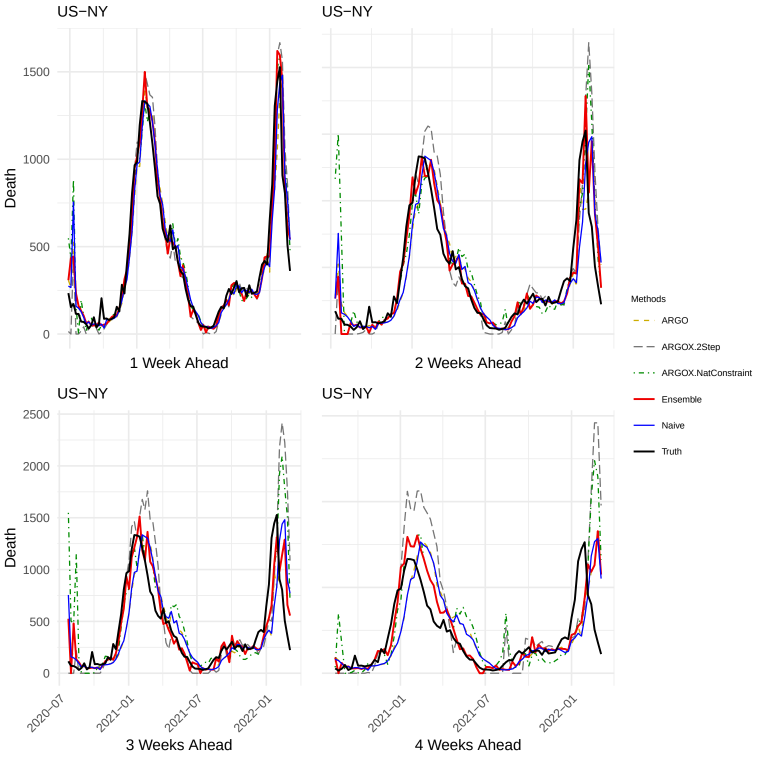

Figure S50: Plots of the COVID-19 1 week (top left), 2 weeks (top right), 3 weeks (bottom left), and 4 weeks (bottom right) ahead estimates for New York (NY).

	1 Week Ahead	2 Weeks Ahead	3 Weeks Ahead	4 Weeks Ahead
RMSE				
ARGO	465.96	503.83	534.62	627.43
ARGOX 2Step	478.65	440.29	496.86	650.59
ARGOX NatConstraint	465.98	411.64	449.58	563.88
Ensemble	488.08	398.19	407.64	512.22
Naive	551.84	583.41	611.42	663.63
MAE				
ARGO	273.03	300.21	325.41	389.44
ARGOX 2Step	244.85	252.08	269.41	374.54
ARGOX NatConstraint	252.32	251.75	252.20	345.57
Ensemble	254.60	239.03	242.47	311.03
Naive	250.50	276.02	306.02	347.76
Correlation				
ARGO	0.37	0.25	0.16	0.07
ARGOX 2Step	0.35	0.40	0.37	0.24
ARGOX NatConstraint	0.33	0.36	0.35	0.18
Ensemble	0.31	0.39	0.38	0.21
Naive	0.27	0.18	0.11	0.03

Table S42: Comparison of different methods for state-level COVID-19 1 to 4 weeks ahead incremental death in Ohio (OH). The MSE, MAE, and correlation are reported.

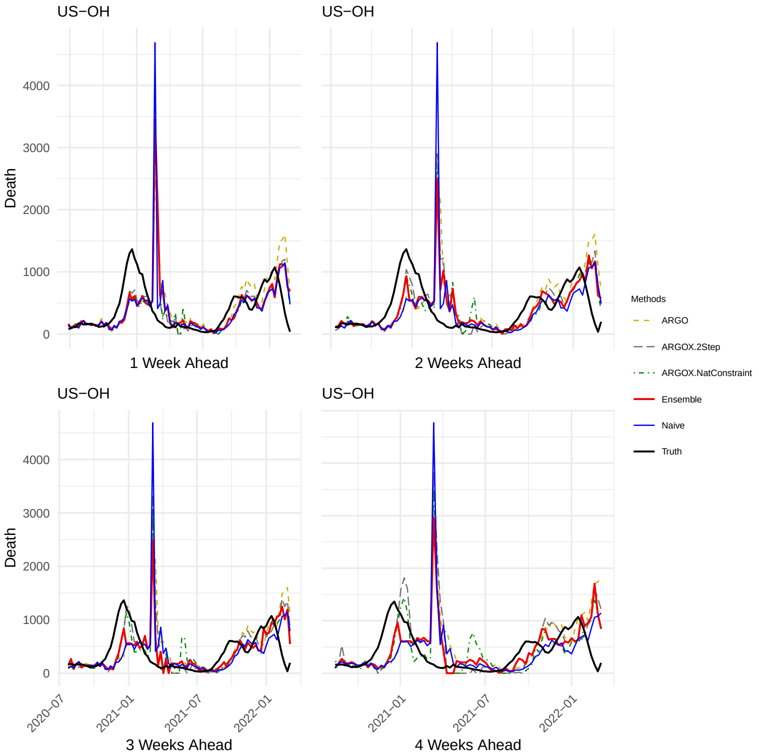

Figure S51: Plots of the COVID-19 1 week (top left), 2 weeks (top right), 3 weeks (bottom left), and 4 weeks (bottom right) ahead estimates for Ohio (OH).

	1 Week Ahead	2 Weeks Ahead	3 Weeks Ahead	4 Weeks Ahead
RMSE				
ARGO	311.55	313.01	284.65	346.47
ARGOX 2Step	288.77	323.93	333.20	380.45
ARGOX NatConstraint	285.72	338.56	351.73	445.63
Ensemble	278.94	294.10	261.30	311.60
Naive	305.23	308.33	289.06	315.81
MAE				
ARGO	116.16	122.98	125.69	153.30
ARGOX 2Step	110.34	141.10	150.42	183.20
ARGOX NatConstraint	120.63	170.76	171.79	206.73
Ensemble	98.43	116.80	109.30	129.22
Naive	109.85	116.76	118.46	141.61
Correlation				
ARGO	0.11	0.11	0.27	0.09
ARGOX 2Step	0.08	0.04	0.19	0.05
ARGOX NatConstraint	0.05	0.03	0.13	0.04
Ensemble	0.13	0.11	0.28	0.11
Naive	0.08	0.07	0.19	0.06

Table S43: Comparison of different methods for state-level COVID-19 1 to 4 weeks ahead incremental death in Oklahoma (OK). The MSE, MAE, and correlation are reported.

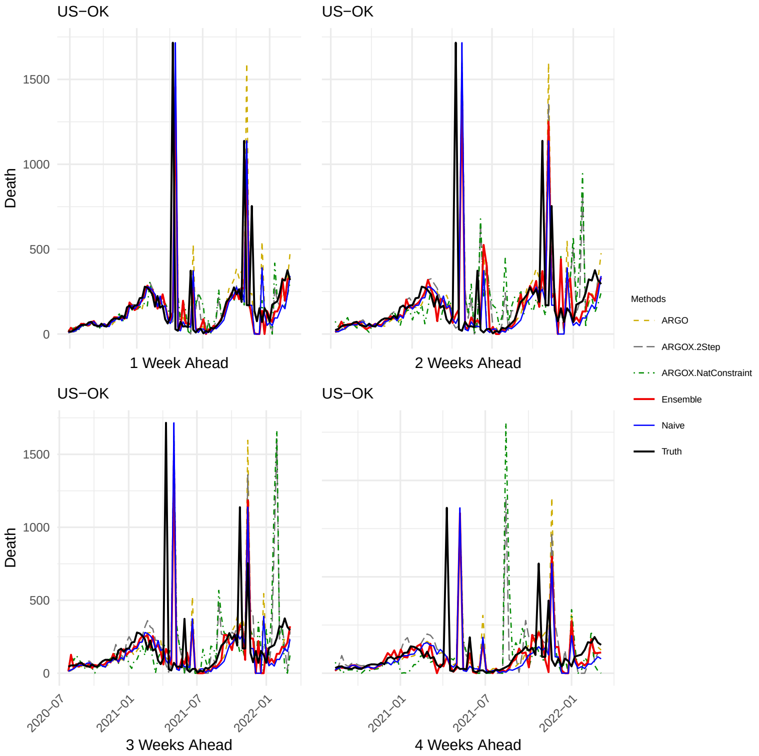

Figure S52: Plots of the COVID-19 1 week (top left), 2 weeks (top right), 3 weeks (bottom left), and 4 weeks (bottom right) ahead estimates for Oklahoma (OK).

62

	1 Week Ahead	2 Weeks Ahead	3 Weeks Ahead	4 Weeks Ahead
RMSE				
ARGO	40.08	40.97	43.95	53.18
ARGOX 2Step	40.56	47.87	68.37	86.46
ARGOX NatConstraint	43.91	52.68	88.14	87.47
Ensemble	37.59	31.64	37.29	45.56
Naive	37.62	43.12	45.53	48.62
MAE				
ARGO	28.90	29.53	32.31	38.46
ARGOX 2Step	27.22	32.04	43.03	54.81
ARGOX NatConstraint	32.32	37.43	59.29	70.51
Ensemble	24.92	22.98	26.86	32.17
Naive	26.00	31.70	33.02	35.13
Correlation				
ARGO	0.75	0.73	0.67	0.63
ARGOX 2Step	0.75	0.69	0.52	0.53
ARGOX NatConstraint	0.67	0.50	0.17	0.22
Ensemble	0.78	0.83	0.76	0.76
Naive	0.77	0.70	0.66	0.62

Table S44: Comparison of different methods for state-level COVID-19 1 to 4 weeks ahead incremental death in Oregon (OR). The MSE, MAE, and correlation are reported.

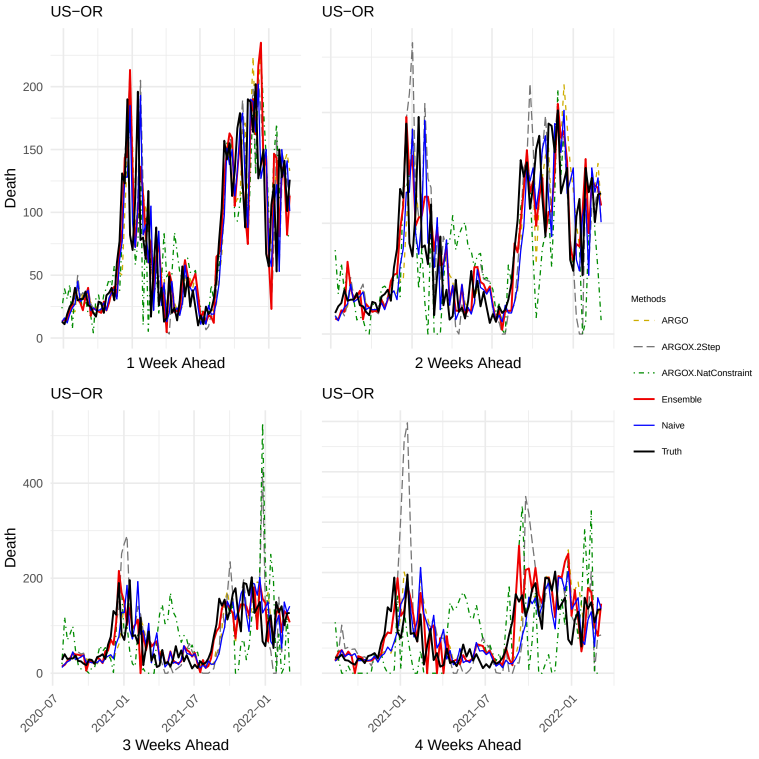

Figure S53: Plots of the COVID-19 1 week (top left), 2 weeks (top right), 3 weeks (bottom left), and 4 weeks (bottom right) ahead estimates for Oregon (OR).

63

	1 Week Ahead	2 Weeks Ahead	3 Weeks Ahead	4 Weeks Ahead
RMSE				
ARGO	149.67	224.74	269.81	358.97
ARGOX 2Step	149.45	236.85	333.90	575.04
ARGOX NatConstraint	128.28	180.87	241.68	411.23
Ensemble	115.87	153.68	207.65	244.45
Naive	126.68	190.54	257.60	342.81
MAE				
ARGO	101.78	140.91	178.28	232.50
ARGOX 2Step	98.86	154.11	223.88	335.09
ARGOX NatConstraint	92.12	127.80	179.07	264.44
Ensemble	76.61	104.11	136.38	160.85
Naive	85.36	123.29	166.16	214.26
Correlation				
ARGO	0.92	0.82	0.75	0.64
ARGOX 2Step	0.94	0.90	0.85	0.78
ARGOX NatConstraint	0.95	0.90	0.86	0.77
Ensemble	0.96	0.93	0.87	0.85
Naive	0.94	0.87	0.77	0.66

Table S45: Comparison of different methods for state-level COVID-19 1 to 4 weeks ahead incremental death in Pennsylvania (PA). The MSE, MAE, and correlation are reported.

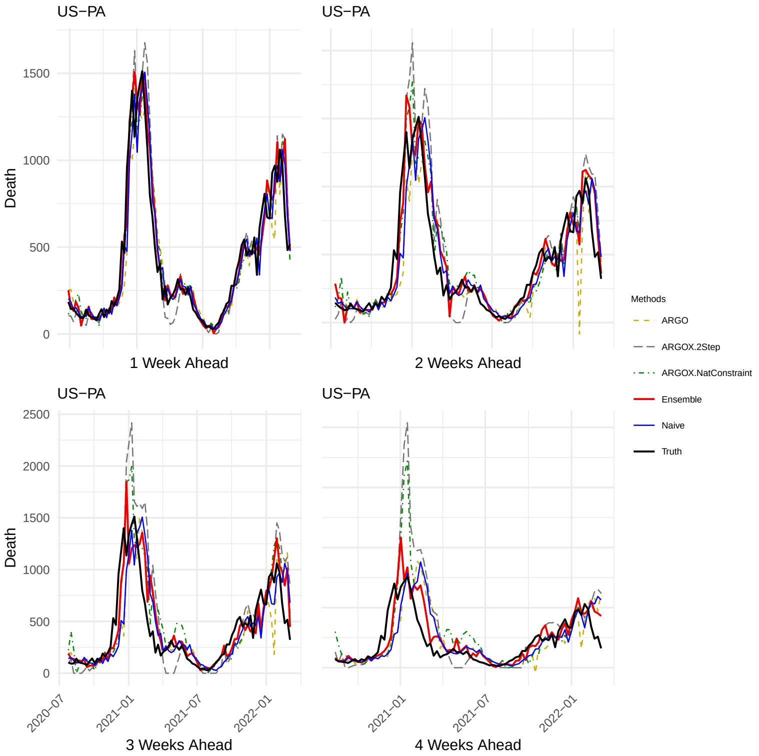

Figure S54: Plots of the COVID-19 1 week (top left), 2 weeks (top right), 3 weeks (bottom left), and 4 weeks (bottom right) ahead estimates for Pennsylvania (PA).

64

	1 Week Ahead	2 Weeks Ahead	3 Weeks Ahead	4 Weeks Ahead
RMSE				
ARGO	20.59	24.17	27.06	31.35
ARGOX 2Step	18.84	23.01	30.44	47.53
ARGOX NatConstraint	30.26	37.19	56.18	72.87
Ensemble	18.78	18.16	19.78	23.05
Naive	19.72	22.72	26.56	30.91
MAE				
ARGO	13.01	14.85	17.62	20.17
ARGOX 2Step	11.35	14.12	19.58	28.69
ARGOX NatConstraint	21.22	27.52	40.40	50.95
Ensemble	11.33	11.87	12.94	15.04
Naive	11.67	13.90	16.44	19.77
Correlation				
ARGO	0.76	0.68	0.61	0.49
ARGOX 2Step	0.83	0.81	0.74	0.64
ARGOX NatConstraint	0.58	0.37	0.17	0.09
Ensemble	0.81	0.84	0.81	0.74
Naive	0.79	0.73	0.63	0.52

Table S46: Comparison of different methods for state-level COVID-19 1 to 4 weeks ahead incremental death in Rhode Island (RI). The MSE, MAE, and correlation are reported.

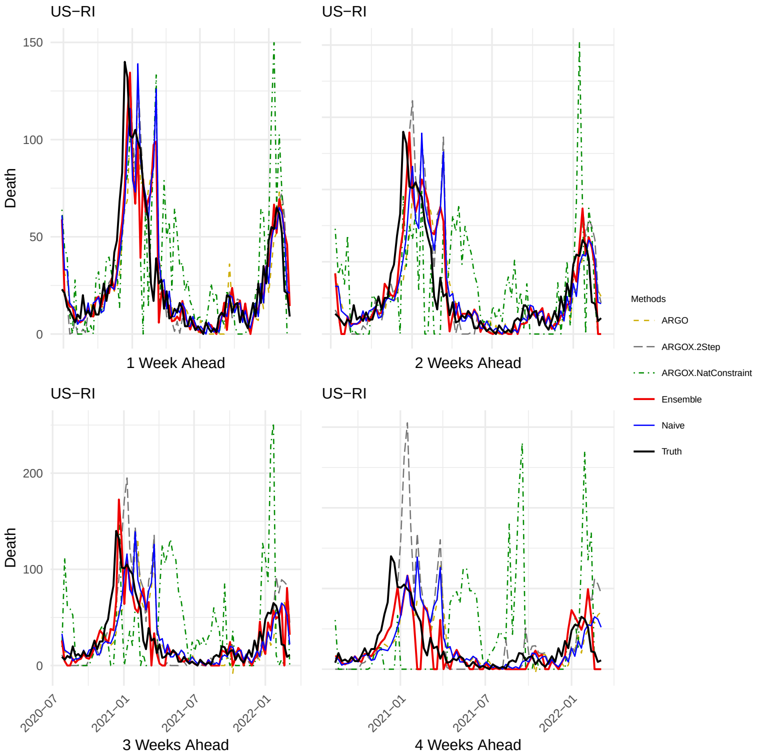

Figure S55: Plots of the COVID-19 1 week (top left), 2 weeks (top right), 3 weeks (bottom left), and 4 weeks (bottom right) ahead estimates for Rhode Island (RI).

65

	1 Week Ahead	2 Weeks Ahead	3 Weeks Ahead	4 Weeks Ahead
RMSE				
ARGO	79.62	167.26	209.05	225.14
ARGOX 2Step	79.46	105.19	139.39	246.74
ARGOX NatConstraint	77.31	90.59	120.48	157.71
Ensemble	71.47	76.56	94.14	105.71
Naive	75.16	98.21	123.95	152.33
MAE				
ARGO	54.34	90.98	121.68	138.01
ARGOX 2Step	50.11	76.64	100.94	169.63
ARGOX NatConstraint	51.26	59.95	86.46	119.38
Ensemble	43.03	49.91	61.95	76.65
Naive	46.92	71.49	92.82	115.54
Correlation				
ARGO	0.81	0.58	0.39	0.18
ARGOX 2Step	0.85	0.81	0.65	0.50
ARGOX NatConstraint	0.82	0.75	0.57	0.48
Ensemble	0.85	0.85	0.76	0.78
Naive	0.85	0.74	0.58	0.43

Table S47: Comparison of different methods for state-level COVID-19 1 to 4 weeks ahead incremental death in South Carolina (SC). The MSE, MAE, and correlation are reported.

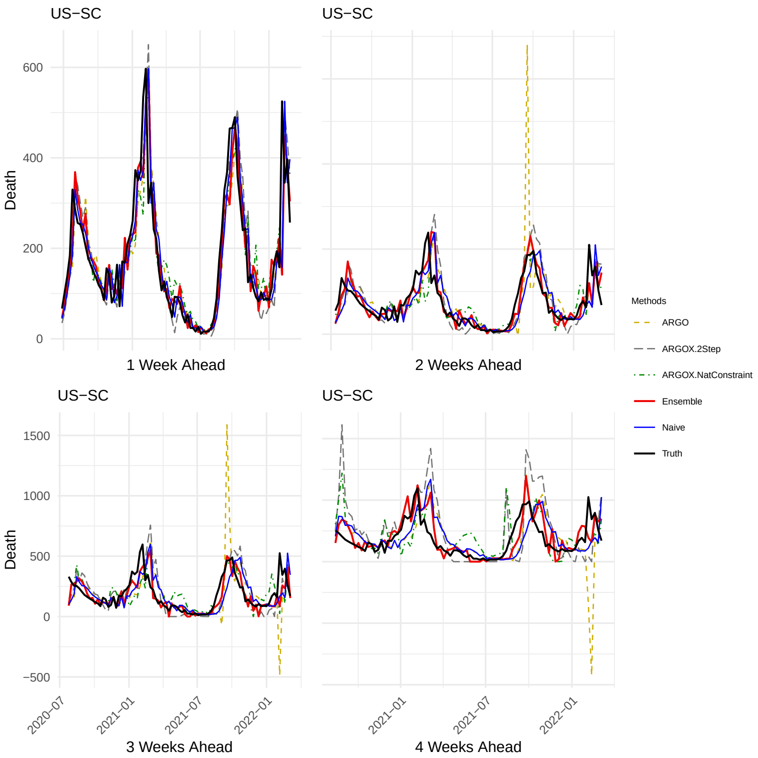

Figure S56: Plots of the COVID-19 1 week (top left), 2 weeks (top right), 3 weeks (bottom left), and 4 weeks (bottom right) ahead estimates for South Carolina (SC).

	1 Week Ahead	2 Weeks Ahead	3 Weeks Ahead	4 Weeks Ahead
RMSE				
ARGO	16.06	22.72	27.85	37.20
ARGOX 2Step	15.59	29.00	44.05	76.02
ARGOX NatConstraint	26.91	36.30	64.72	77.73
Ensemble	11.44	14.08	17.01	21.61
Naive	13.63	18.26	25.41	37.02
MAE				
ARGO	9.83	13.16	16.99	22.49
ARGOX 2Step	9.73	14.77	18.88	29.90
ARGOX NatConstraint	20.19	27.00	45.89	53.20
Ensemble	7.98	9.46	10.12	13.90
Naive	8.44	11.13	15.52	21.56
Correlation				
ARGO	0.90	0.80	0.69	0.56
ARGOX 2Step	0.94	0.87	0.76	0.63
ARGOX NatConstraint	0.77	0.66	0.39	0.24
Ensemble	0.95	0.93	0.89	0.87
Naive	0.93	0.87	0.76	0.63

Table S48: Comparison of different methods for state-level COVID-19 1 to 4 weeks ahead incremental death in South Dakota (SD). The MSE, MAE, and correlation are reported.

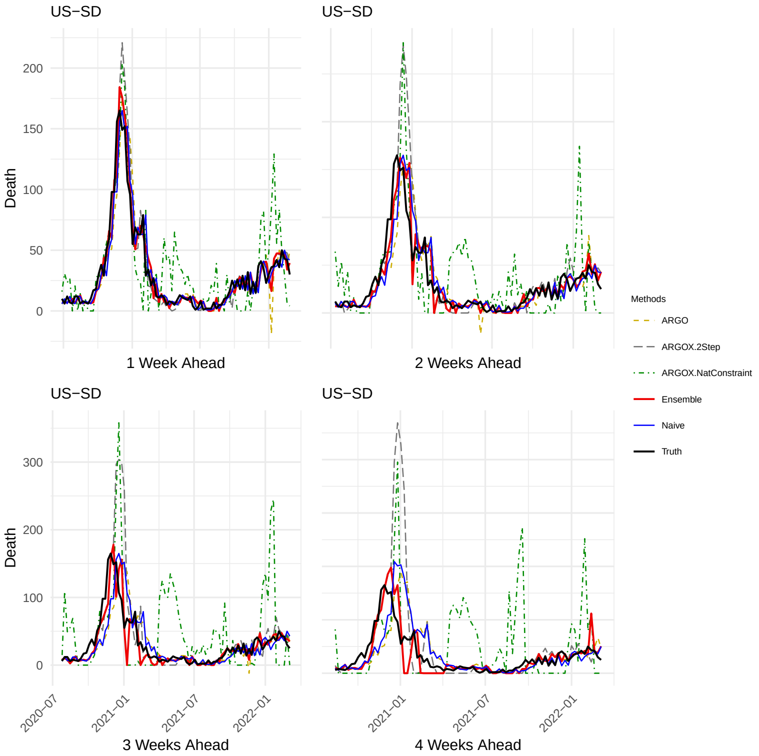

Figure S57: Plots of the COVID-19 1 week (top left), 2 weeks (top right), 3 weeks (bottom left), and 4 weeks (bottom right) ahead estimates for South Dakota (SD).

	1 Week Ahead	2 Weeks Ahead	3 Weeks Ahead	4 Weeks Ahead
RMSE				
ARGO	312.36	326.75	345.61	361.40
ARGOX 2Step	342.65	392.54	408.19	421.39
ARGOX NatConstraint	347.96	388.08	396.59	391.78
Ensemble	320.57	316.91	336.37	330.93
Naive	327.38	362.76	352.46	368.42
MAE				
ARGO	130.12	149.37	169.04	186.70
ARGOX 2Step	137.59	173.36	189.23	233.74
ARGOX NatConstraint	140.51	170.46	171.03	206.45
Ensemble	122.56	125.85	134.56	150.50
Naive	120.95	149.11	150.20	171.82
Correlation				
ARGO	0.41	0.34	0.31	0.31
ARGOX 2Step	0.38	0.31	0.23	0.32
ARGOX NatConstraint	0.33	0.24	0.18	0.27
Ensemble	0.40	0.39	0.35	0.46
Naive	0.40	0.27	0.31	0.30

Table S49: Comparison of different methods for state-level COVID-19 1 to 4 weeks ahead incremental death in Tennessee (TN). The MSE, MAE, and correlation are reported.

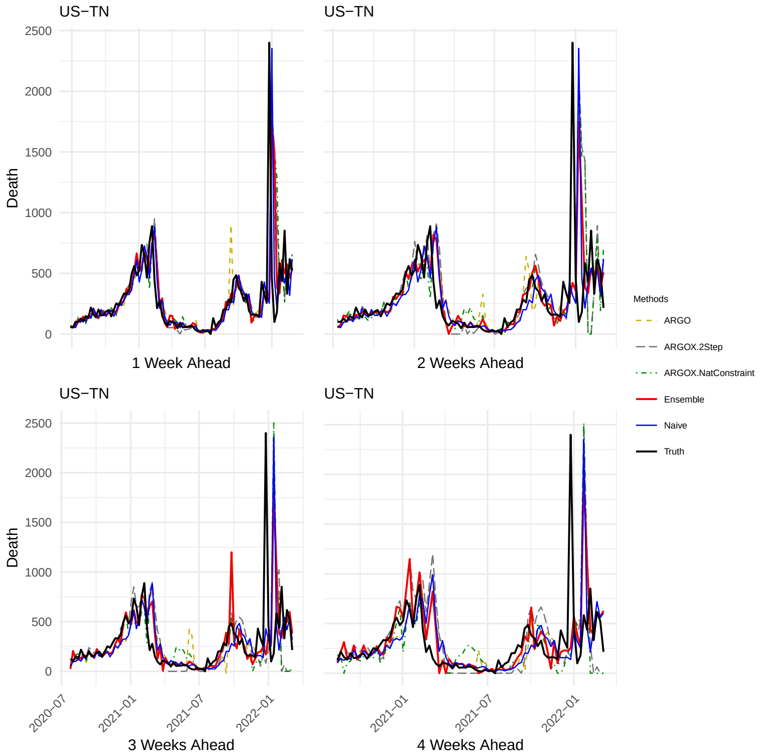

Figure S58: Plots of the COVID-19 1 week (top left), 2 weeks (top right), 3 weeks (bottom left), and 4 weeks (bottom right) ahead estimates for Tennessee (TN).

	1 Week Ahead	2 Weeks Ahead	3 Weeks Ahead	4 Weeks Ahead
RMSE				
ARGO	280.42	390.22	430.26	592.57
ARGOX 2Step	309.44	450.63	566.20	1154.04
ARGOX NatConstraint	281.11	383.58	435.94	906.80
Ensemble	244.41	333.96	363.25	526.69
Naive	285.57	419.63	502.19	645.30
MAE				
ARGO	210.26	298.67	336.26	473.26
ARGOX 2Step	212.66	333.41	423.12	797.45
ARGOX NatConstraint	198.05	288.90	329.65	618.13
Ensemble	176.75	243.07	262.68	373.78
Naive	199.43	308.31	390.42	512.54
Correlation				
ARGO	0.89	0.77	0.72	0.61
ARGOX 2Step	0.90	0.84	0.76	0.57
ARGOX NatConstraint	0.89	0.81	0.75	0.55
Ensemble	0.92	0.86	0.82	0.75
Naive	0.89	0.75	0.65	0.52

Table S50: Comparison of different methods for state-level COVID-19 1 to 4 weeks ahead incremental death in Texas (TX). The MSE, MAE, and correlation are reported.

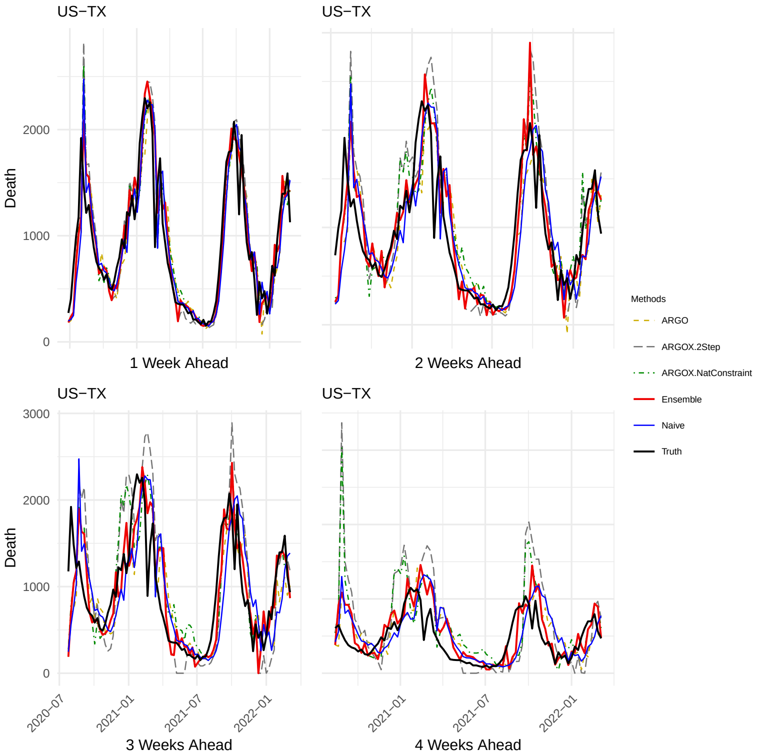

Figure S59: Plots of the COVID-19 1 week (top left), 2 weeks (top right), 3 weeks (bottom left), and 4 weeks (bottom right) ahead estimates for Texas (TX).

69

	1 Week Ahead	2 Weeks Ahead	3 Weeks Ahead	4 Weeks Ahead
RMSE				
ARGO	17.07	19.77	20.87	26.32
ARGOX 2Step	17.78	23.04	24.44	42.51
ARGOX NatConstraint	29.81	41.38	65.12	76.77
Ensemble	14.61	19.69	17.62	24.19
Naive	17.23	19.73	20.64	24.80
MAE				
ARGO	13.30	16.41	17.45	22.41
ARGOX 2Step	13.05	17.44	19.19	30.89
ARGOX NatConstraint	22.78	32.20	51.90	62.23
Ensemble	11.21	14.39	14.36	19.89
Naive	12.45	15.94	17.02	21.51
Correlation				
ARGO	0.83	0.75	0.73	0.65
ARGOX 2Step	0.82	0.77	0.80	0.63
ARGOX NatConstraint	0.51	0.17	0.00	0.09
Ensemble	0.88	0.78	0.82	0.79
Naive	0.81	0.75	0.72	0.63

Table S51: Comparison of different methods for state-level COVID-19 1 to 4 weeks ahead incremental death in Utah (UT). The MSE, MAE, and correlation are reported.

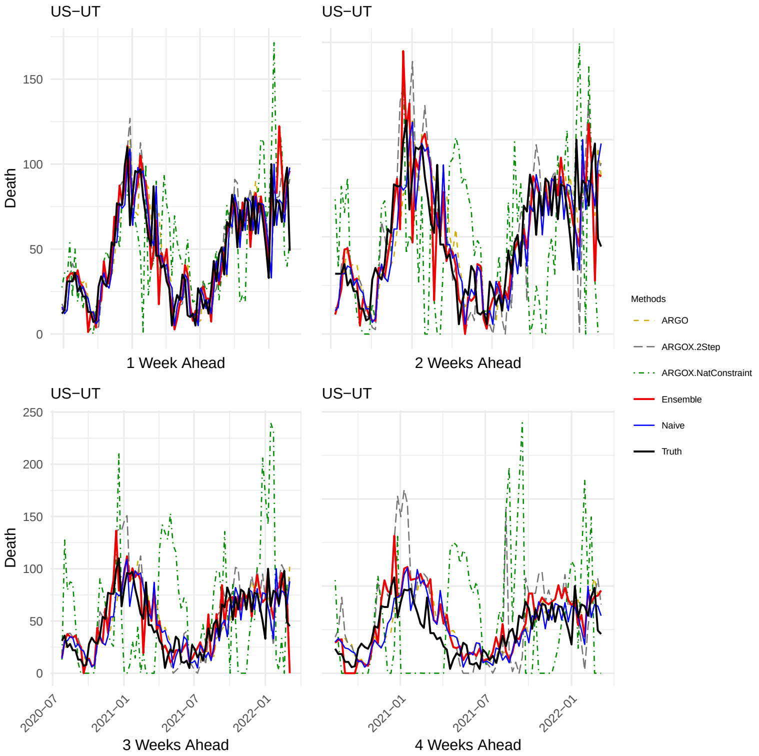

Figure S60: Plots of the COVID-19 1 week (top left), 2 weeks (top right), 3 weeks (bottom left), and 4 weeks (bottom right) ahead estimates for Utah (UT).

	1 Week Ahead	2 Weeks Ahead	3 Weeks Ahead	4 Weeks Ahead
RMSE				
ARGO	151.47	246.90	249.92	268.66
ARGOX 2Step	179.75	259.08	288.25	319.86
ARGOX NatConstraint	173.18	262.00	298.59	310.07
Ensemble	153.92	242.86	237.46	256.52
Naive	153.13	227.51	253.48	264.73
MAE				
ARGO	73.59	121.33	132.56	149.63
ARGOX 2Step	85.65	133.76	146.14	174.67
ARGOX NatConstraint	83.37	147.00	161.94	188.32
Ensemble	70.55	111.83	109.61	123.39
Naive	72.26	112.21	130.76	138.82
Correlation				
ARGO	0.69	0.28	0.20	0.18
ARGOX 2Step	0.64	0.33	0.20	0.20
ARGOX NatConstraint	0.62	0.16	0.03	0.05
Ensemble	0.69	0.33	0.26	0.27
Naive	0.70	0.35	0.20	0.19

Table S52: Comparison of different methods for state-level COVID-19 1 to 4 weeks ahead incremental death in Virginia (VA). The MSE, MAE, and correlation are reported.

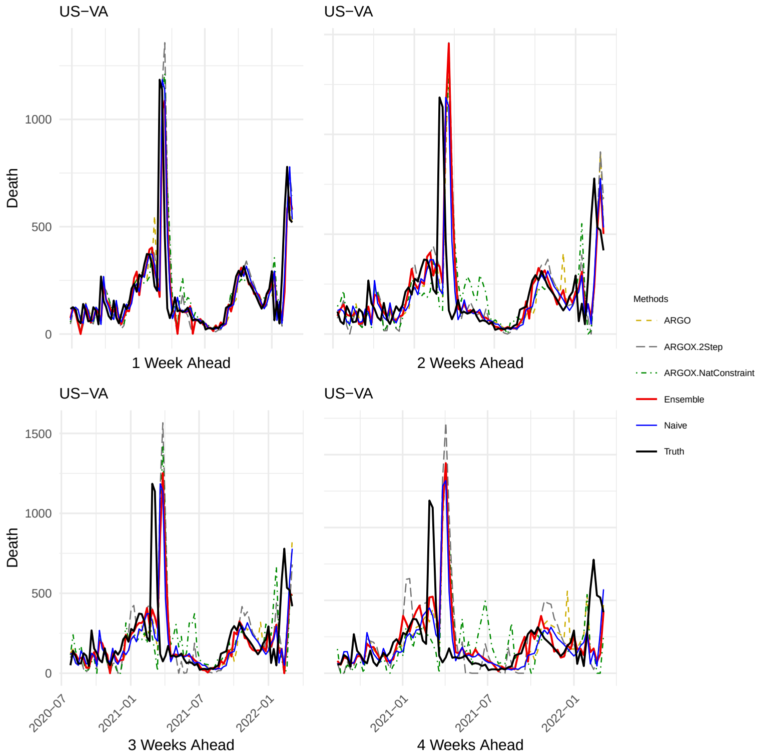

Figure S61: Plots of the COVID-19 1 week (top left), 2 weeks (top right), 3 weeks (bottom left), and 4 weeks (bottom right) ahead estimates for Virginia (VA).

71

	1 Week Ahead	2 Weeks Ahead	3 Weeks Ahead	4 Weeks Ahead
RMSE				
ARGO	4.28	4.64	5.19	5.32
ARGOX 2Step	3.63	3.73	4.62	4.80
ARGOX NatConstraint	4.28	4.64	5.19	5.32
Ensemble	3.90	4.43	4.79	5.86
Naive	4.08	4.65	5.43	5.50
MAE				
ARGO	2.70	2.99	3.31	3.74
ARGOX 2Step	2.12	2.36	2.77	3.21
ARGOX NatConstraint	2.70	2.99	3.31	3.74
Ensemble	2.37	2.84	3.03	3.95
Naive	2.58	3.13	3.45	4.00
Correlation				
ARGO	0.79	0.74	0.66	0.71
ARGOX 2Step	0.86	0.85	0.77	0.77
ARGOX NatConstraint	0.79	0.74	0.66	0.71
Ensemble	0.84	0.80	0.73	0.76
Naive	0.78	0.72	0.61	0.63

Table S53: Comparison of different methods for state-level COVID-19 1 to 4 weeks ahead incremental death in Vermont (VT). The MSE, MAE, and correlation are reported.

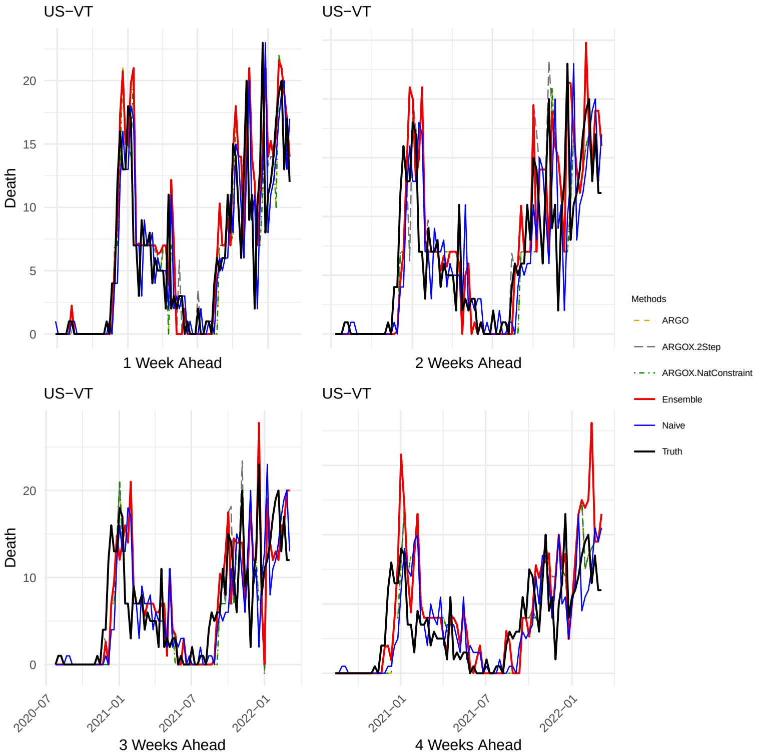

Figure S62: Plots of the COVID-19 1 week (top left), 2 weeks (top right), 3 weeks (bottom left), and 4 weeks (bottom right) ahead estimates for Vermont (VT).

72

	1 Week Ahead	2 Weeks Ahead	3 Weeks Ahead	4 Weeks Ahead
RMSE				
ARGO	53.03	53.47	58.71	81.49
ARGOX 2Step	47.86	55.27	71.59	111.67
ARGOX NatConstraint	48.46	58.04	83.42	83.10
Ensemble	41.20	35.13	47.86	58.73
Naive	43.48	49.71	66.14	74.20
MAE				
ARGO	33.78	38.91	46.38	64.44
ARGOX 2Step	32.59	40.43	52.54	87.20
ARGOX NatConstraint	35.58	41.95	61.35	65.85
Ensemble	26.94	26.33	34.64	44.27
Naive	29.94	37.93	50.25	57.90
Correlation				
ARGO	0.82	0.81	0.74	0.59
ARGOX 2Step	0.85	0.83	0.71	0.62
ARGOX NatConstraint	0.82	0.72	0.45	0.56
Ensemble	0.88	0.91	0.83	0.82
Naive	0.86	0.81	0.66	0.57

Table S54: Comparison of different methods for state-level COVID-19 1 to 4 weeks ahead incremental death in Washington (WA). The MSE, MAE, and correlation are reported.

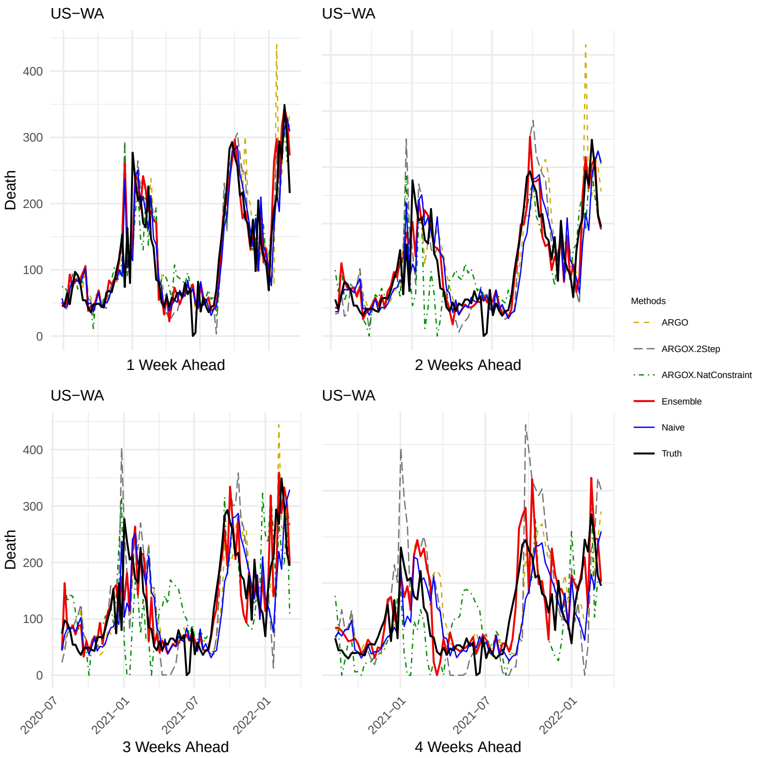

Figure S63: Plots of the COVID-19 1 week (top left), 2 weeks (top right), 3 weeks (bottom left), and 4 weeks (bottom right) ahead estimates for Washington (WA).

73

	1 Week Ahead	2 Weeks Ahead	3 Weeks Ahead	4 Weeks Ahead
RMSE				
ARGO	60.87	79.32	73.81	94.60
ARGOX 2Step	59.14	74.62	97.63	159.20
ARGOX NatConstraint	58.67	77.55	108.74	150.12
Ensemble	56.20	63.04	67.65	87.01
Naive	51.99	65.62	77.28	97.34
MAE				
ARGO	41.70	52.91	53.26	66.95
ARGOX 2Step	39.78	52.73	67.45	96.76
ARGOX NatConstraint	42.01	60.29	84.65	117.55
Ensemble	34.74	44.81	46.48	57.63
Naive	36.77	48.54	57.07	69.26
Correlation				
ARGO	0.86	0.78	0.79	0.70
ARGOX 2Step	0.89	0.86	0.84	0.73
ARGOX NatConstraint	0.87	0.78	0.68	0.56
Ensemble	0.89	0.86	0.84	0.76
Naive	0.90	0.83	0.77	0.69

Table S55: Comparison of different methods for state-level COVID-19 1 to 4 weeks ahead incremental death in Wisconsin (WI). The MSE, MAE, and correlation are reported.

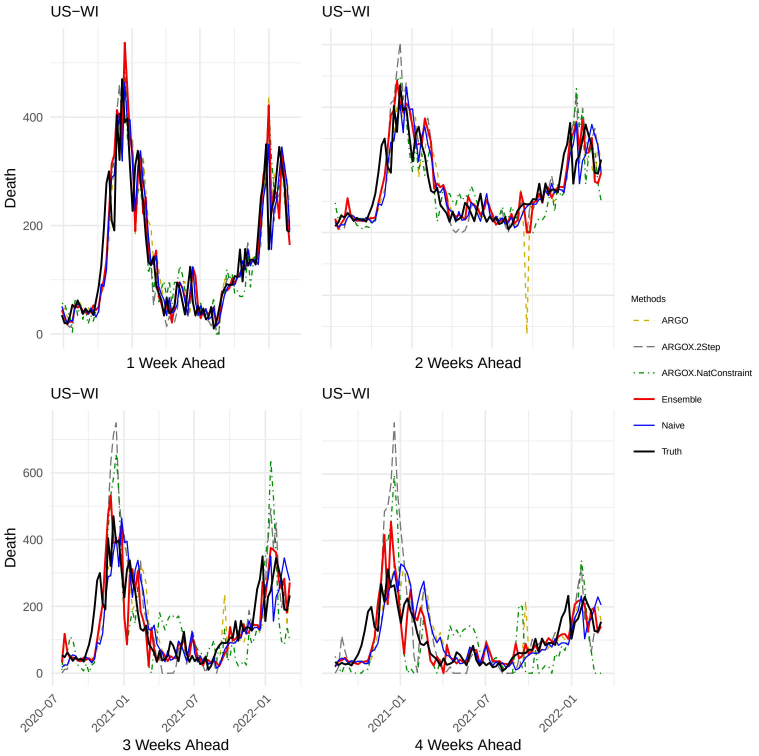

Figure S64: Plots of the COVID-19 1 week (top left), 2 weeks (top right), 3 weeks (bottom left), and 4 weeks (bottom right) ahead estimates for Wisconsin (WI).

	1 Week Ahead	2 Weeks Ahead	3 Weeks Ahead	4 Weeks Ahead
RMSE				
ARGO	36.46	46.81	43.54	56.27
ARGOX 2Step	37.65	45.15	49.59	89.61
ARGOX NatConstraint	46.05	63.93	80.79	102.68
Ensemble	31.95	38.76	32.49	54.27
Naive	34.37	42.08	45.64	54.95
MAE				
ARGO	24.24	30.77	31.88	40.65
ARGOX 2Step	24.61	32.40	34.78	65.38
ARGOX NatConstraint	31.94	48.40	60.37	82.74
Ensemble	20.63	27.61	22.68	38.48
Naive	22.41	29.10	32.74	40.23
Correlation				
ARGO	0.80	0.70	0.70	0.56
ARGOX 2Step	0.81	0.77	0.76	0.56
ARGOX NatConstraint	0.66	0.39	0.25	0.02
Ensemble	0.85	0.80	0.84	0.63
Naive	0.83	0.74	0.69	0.56

Table S56: Comparison of different methods for state-level COVID-19 1 to 4 weeks ahead incremental death in West Virginia (WV). The MSE, MAE, and correlation are reported.

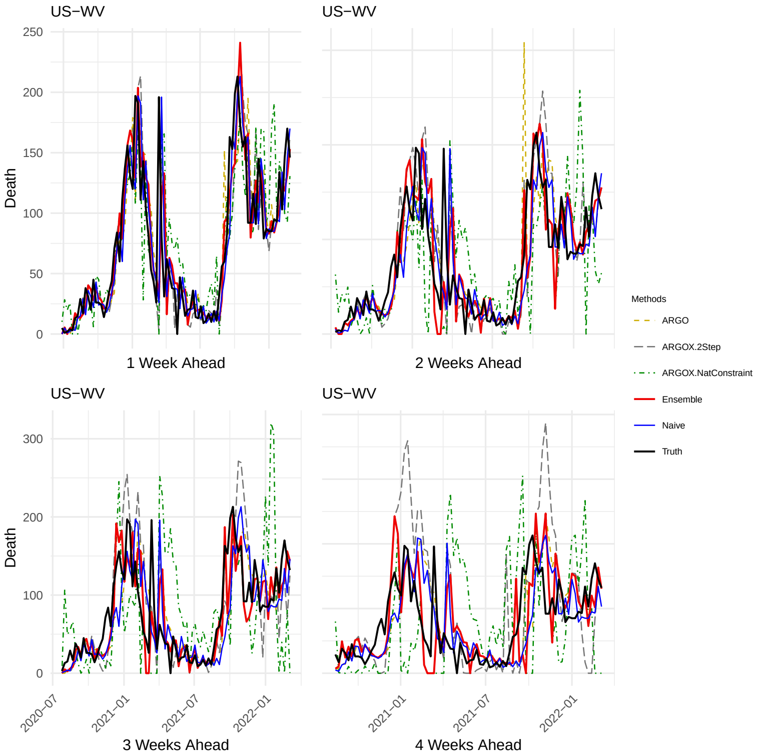

Figure S65: Plots of the COVID-19 1 week (top left), 2 weeks (top right), 3 weeks (bottom left), and 4 weeks (bottom right) ahead estimates for West Virginia (WV).

	1 Week Ahead	2 Weeks Ahead	3 Weeks Ahead	4 Weeks Ahead
RMSE				
ARGO	17.16	20.81	21.91	25.75
ARGOX 2Step	14.63	14.86	18.84	21.66
ARGOX NatConstraint	28.98	36.25	58.94	71.20
Ensemble	14.52	15.32	19.69	16.41
Naive	17.12	16.27	16.57	17.59
MAE				
ARGO	10.10	13.39	13.16	16.89
ARGOX 2Step	8.02	8.72	10.39	12.54
ARGOX NatConstraint	20.45	27.88	40.10	47.87
Ensemble	8.07	9.72	10.09	9.90
Naive	10.03	10.48	11.22	12.43
Correlation				
ARGO	0.62	0.65	0.60	0.60
ARGOX 2Step	0.72	0.80	0.73	0.70
ARGOX NatConstraint	0.20	0.02	0.15	0.14
Ensemble	0.72	0.75	0.69	0.79
Naive	0.62	0.65	0.64	0.61

Table S57: Comparison of different methods for state-level COVID-19 1 to 4 weeks ahead incremental death in Wyoming (WY). The RMSE, MAE, and correlation are reported.

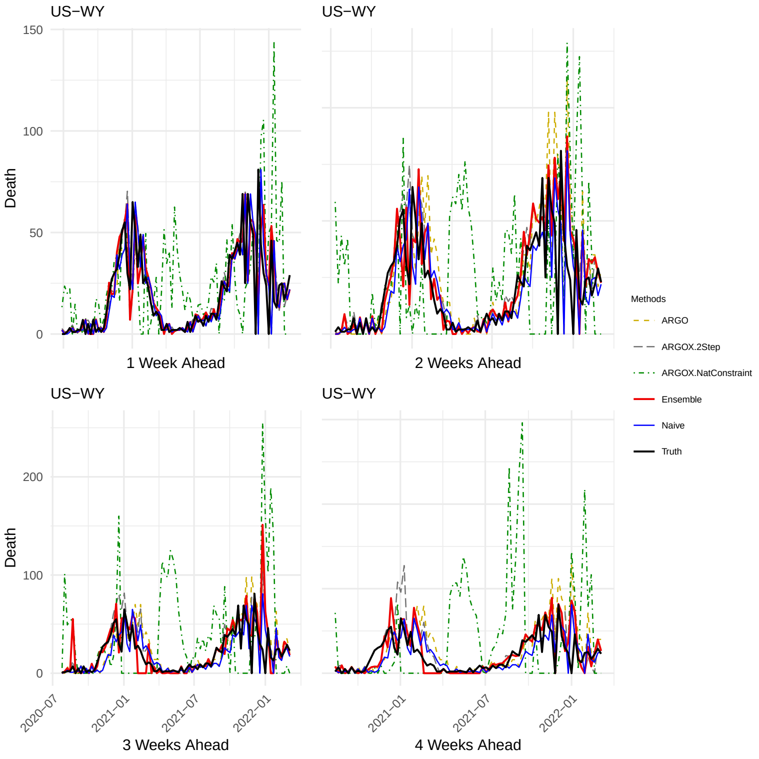

Figure S66: Plots of the COVID-19 1 week (top left), 2 weeks (top right), 3 weeks (bottom left), and 4 weeks (bottom right) ahead estimates for Wyoming (WY).
